# Preparedness and Preventive Behaviors for a Pandemic Disaster Caused by COVID-19 in Serbia

**DOI:** 10.3390/ijerph17114124

**Published:** 2020-06-09

**Authors:** Vladimir M. Cvetković, Neda Nikolić, Una Radovanović Nenadić, Adem Öcal, Eric K. Noji, Miodrag Zečević

**Affiliations:** 1 Faculty of Security Studies, University of Belgrade, Gospodara Vučića 50, 11040 Belgrade, Serbia; 2 Faculty of Tehnical Science in Čačak, University of Kragujevac, 32000 Čačak, Serbia; neda.nikolic@ftn.kg.ac.rs; 3 Scientific-Professional Society for Disaster Risk Management, Dimitrija Tucovića 121, 11040 Belgrade, Serbia; una.radovanovic@gmail.com; 4 Independent Researcher, Ankara 06500, Turkey; ocadem@gmail.com; 5 King Saud University Hospitals and College of Medicine, Riyadh 11564, Saudi Arabia; nojie@alumni.stanford.edu; 6 Faculty of International Engineering Management Belgrade, European University, 11000 Belgrade, Serbia; misko.zecevic@gmail.com

**Keywords:** disaster, epidemic risk, pandemic, citizen preparedness, coronavirus, COVID-19, Serbia

## Abstract

Coronavirus disease 2019 (COVID-19) is an infectious disease caused by severe acute respiratory syndrome coronavirus 2. The disease was first detected in Wuhan, the capital of China’s Hubei province, in December 2019 and has since spread globally, especially to Europe and North America, resulting in the ongoing global coronavirus pandemic disaster of 2019–2020. Although most cases have mild symptoms, there is some progression to viral pneumonia and multi-organ failure and death. More than 4.6 million cases have been registered across 216 countries and territories as of 19 April 2020, resulting in more than 311,000 deaths. Risk to communities with continued widespread disease transmission depends on characteristics of the virus, including how well it spreads between people; the severity of resulting illness; and the medical or other measures available to control the impact of the virus (for example, vaccines or medications that can treat the illness) and the relative success of these. In the absence of vaccines or medications, non-pharmaceutical interventions were the most important response strategy based on community interventions such as person-to-person distancing, mask-wearing, isolation and good personal hygiene (hand-washing)—all of which have been demonstrated can reduce the impact of this seemingly unstoppable globally spreading natural disaster. This paper presents the results of quantitative research regarding the level of citizen preparedness for disasters caused by coronavirus disease (COVID-19) in Serbia. The survey was conducted using a questionnaire that was requested and then collected online among 975 respondents during disaster in April 2020. The questionnaire examined citizens’ basic socio-economic and demographic characteristics, their knowledge, preparedness, risk perception and preventive measures taken individually and as a community to prevent the death and widespread transmission of novel coronavirus disease 2019 in the Republic of Serbia. Based on the findings that there are major differences in the public’s perception of risks posed by communicable disease threats such as presented by COVID-19, emergency management agencies should use these differences to develop targeted strategies to enhance community and national preparedness by promoting behavioral change and improving risk management decision-making.

## 1. Introduction

COVID-19 is a new strain of coronavirus that has not been previously identified in humans. Coronaviruses are zoonotic and are a large family of viruses that cause illness ranging from the common colds to more serious diseases, such as MERS and SARS [1]. Compared with SARS and MERS, COVID-19 has spread more rapidly, mainly due to increased globalization and the focus of the epidemic [2]. COVID-19 may have acted differently from previous coronavirus epidemics. Firstly, the lack of organized, systematic and scientific knowledge about COVID-19 caused anxieties on the part of individuals and governments as they were facing an unseen enemy around the world [3]. In addition, the main phenotype of this outbreak is the high rate of spread, age and increased vulnerability of low immune individuals and the differential rate of recovery [4].

On 7 January 2020, the Chinese CDC isolated a novel coronavirus from the throat swab of a patient with pneumonia of unknown etiology. The new virus and disease were unknown before the outbreak began in Wuhan, China, in December 2019, but the novel virus was identified by the World Health Organization (WHO) as the 2019-novel Coronavirus (nCoV). On 11 February 2020, WHO formally named the disease caused by SARS-CoV-2 virus as “COVID-19” [5]. Two pathogenic coronaviruses were mainly known before December 2019. One is severe acute respiratory coronavirus syndrome (SARS-CoV) and the other is Middle East respiratory coronavirus syndrome (MERS-CoV). SARS was first identified in China in 2002, with 32 countries reporting cases worldwide and 8442 people infected with SARS, of whom 916 died, for a case–fatality rate (CFT) of 11% worldwide, but the CFT was 27% in China [1]. MERS was identified in the Middle East (Saudi Arabia) region in 2012. By the end of November 2019, a total of 2494 laboratory-confirmed cases of MERS, including 858 related deaths (case–fatality rate: 34.4%) were reported in 27 countries; the majority of these cases were reported from Saudi Arabia [1]. Other known coronaviruses (HCoV-OC43, HCoV-229E, HCoV-NL63, HCoV-HKU1) induce mild or asymptomatic upper respiratory tract disease like the common cold [6].

The outbreak of COVID-19 spread far faster than before around the world. As of 11 February 2020, there have been 43,118 laboratory-confirmed cases of novel coronavirus (2019-nCoV) infected pneumonia (NCIP) worldwide, with 1,018 deaths [7]. Cases have been reported from 14 Asian countries, nine European countries, two North American countries and one country in Oceania. There have been 1017 deaths in China and 1 in the Philippines outside of China [7]. All reported cases have been confirmed by real-time reverse transcriptase-polymerase chain reaction [8,9].

The total global number of COVID-19 cases has exceeded 4.6 million, and total number of death is 311,847 [10]. Since the declaration of COVID-19 as a public health emergency of international concern, the number of countries implementing additional health measures that significantly interfere with international traffic has increased. COVID-19 is a new disease that is distinct from other SARS, MERS, and influenza diseases. Although coronavirus and influenza infections may have similar symptoms, the virus responsible for COVID-19 differs in terms of community spread and severity. Much remains to be discovered about the disease and its impact in different contexts. Preparedness, readiness, and response actions will continue to be driven by the rapid accumulation of scientific and public health knowledge. 

Every country should urgently take all necessary measures to slow further spread and prevent its health systems from being overwhelmed by seriously ill patients with COVID-19. Although the government was convinced that Serbia would not be largely affected by the COVID-19, the state of emergency was declared on 15 March, not long after the first case had been registered. The first officially registered case on 6 March is most likely to be imported from Budapest, as the male patient in question visited his ill sister (with “respiratory problems, fever and other COVID-19 symptoms”), as stated by the Minister of Health Zlatibor Lončar [11]. Subsequently, a significant number of provisions and orders were made, among the most important ones [12]: compulsory 28-day self-isolation was imposed for anyone entering the country on 14 March 2020 and later.

Social media and networks monitoring and addressing’s of the government representatives clearly indicated difficulties in complying with the envisaged measures, particularly in the first weeks leading up to the state of emergency. This could probably have been the result of changes of point of view in this less than a month long period, which could lead to the confusion among the general public [12]. Although Serbian officials followed WHO Guidelines and collaborated with the experts from the China following the declaration of the state of emergency, and have urged Serbian citizens to do the same, the public was not prepared for an epidemic. Even though history testifies to a small number of serious epidemics and researchers have found evidences of poor knowledge and preparedness of Serbian public about epidemics [13,14] and infectious agents used as biological weapons [15], little has been done to prevent epidemics and pandemics. Given that Serbia suffered great losses in the May 2014 floods, the most of the government attention was focused to emergency management in general. However, the right to education in emergency situations was not affirmed and recognized in the past [16]. The need to conduct a survey on the preparedness of citizens for outbreaks of a major epidemiological significance (declared on 19 March) on the territory of the Republic of Serbia has therefore been recognized. 

### 1.1. Literature Review

Understanding individuals’ behavior and their relationship to their perceived risk is important in terms of effective control of an outbreak of infectious diseases [17]. Some studies have examined risk perceptions and precautionary behaviors during the early stages of the 2009 (H1N1) influenza pandemic in Hong Kong [18,19], Australia [20], France [21,22], and the England, Scotland, and Wales [20]. These studies found that precautionary behaviors were associated with anxiety about H1N1 influenza, risk perceptions, perceived efficacy of the precautionary behaviors [19,23].

There are some scientific studies related to perceptions of people about pandemic diseases, their precautions and preparations. Balicer et al. [24] conducted a study to understand local public health workers’ perceptions of pandemic influenza response. They surveyed 308 staff at three health departments in Maryland from March–July 2005, about factors that could influence their ability and willingness to report to duty in this event. The study demonstrated that almost half of the local health department workers were likely not to report to duty during a pandemic. The perceived risk among public health workers has been shown to be associated with several factors that are peripheral to the actual hazard of this event. These changes in the risk perception and the knowledge gaps identified serve as barriers to the response to a influenza pandemic and must be specifically addressed to enable effective local public health response to this significant threat. Barr et al. [25], investigated how a population perceives the threat of influenza pandemic, and what was their preparedness to comply with specific public health behaviours in the event of pandemic influenza. 

The survey was completed on a representative sample of 2081 adults. In the event of a flu pandemic, the majority of the population was willing to be vaccinated (75.4%), be isolated (70.2%), and wear a face mask (59.9%). People with higher levels of risk perception are significantly more likely to be willing to comply with specific public health behaviours. While only 14.9% of the New South Wales (Australia) population thought that a pandemic influenza was very or extremely likely to occur, a significantly higher proportion of the population and the family were concerned if a pandemic actually occurred. Ibuka et al. [26] assessed temporal changes and geographical differences in risk perceptions and precautionary behaviors in response to H1N1 influenza. An online survey of risk perceptions, interests in pharmaceutical interventions (preventive intervention and curative intervention), and involvement in precautionary activities (information seeking activities and taking quarantine measures) in response to H1N1 influenza was conducted in 2009. Geographical differences in risk perception and precautionary behaviors have been evaluated. Predictors of willingness to take part in a pharmaceutical intervention were analyzed. The perceived likelihood of influenza infection, willingness to take drugs, and involvement in information seeking activities was higher for women than men. The perceived risk of infection and precautionary behavior can be dynamic over time, and differ by demographic characteristics and geographical locations. These patterns are likely to influence the effectiveness of disease control measures. Park et al. [27] conducted a study to assess the perceptions, motivational factors and behaviors associated with the use of hand washing to prevent H1N1 influenza transmission during the peak pandemic period in Korea. They reached the point that Korean students increased their frequency of hand hygiene practices during the pandemic, with significant gender differences existing in the attitudes and behaviors related to the use of hand hygiene as a means of disease prevention. The factors that affected hand washing behavior were similar to those identified at the beginning of the H1N1 or SARS pandemics, suggesting that public education campaigns regarding hand hygiene are effective in altering individual hand hygiene habits during the peak periods of influenza transmission. 

Kwok et al. [28] examined the community’s psychological and behavioral responses during the early phase of the Hong Kong epidemic of COVID-19. Analysis of 1715 complete responses indicated high perceived susceptibility (89%) and high perceived severity (97%). Most of the respondents were worried about COVID-19 (97%), and had their daily routines disrupted (slightly/greatly: 98%). The level of anxiety, measured by the Hospital Anxiety and Depression Scale, was borderline abnormal (9.01). Almost all respondents were alert to the disease progression (99.5%). The most reliable sources of information were doctors (84%), followed by broadcasts (57%) and newspapers (54%), but they were not common sources of information (doctor: 5%, broadcast: 34%, newspaper: 40%). Only 16% respondents found official websites to be reliable. Enhanced personal hygiene and travel-avoidance practices to China have frequently been adopted (>77%) and considered effective (>90%). The adoption of social-distancing measures was lower (39–88%), and their drivers for increased adoption include: being female (adjusted odds ratio [aOR]:1.27), living in the New Territories (aOR:1.32–1.55), perceived as having good understanding of COVID-19 (aOR:1.84) and being more anxious (aOR:1.07).

Gilbert et al. [29] assessed the preparedness and vulnerability of African countries to their risk of importation of COVID-19. The risk of importation from China of cases of COVID-19 to Africa was estimated on the basis of the origin–destination air travel flows from January 2019; number of cases in Chinese provinces; and the population in each of the Chinese provinces that reported transmission. They identified the country’s capacity to detect and respond to cases with two indicators: preparedness, and vulnerability. Countries were clustered according to the Chinese regions that contributed most to their risk. The authors identified three clusters of countries (i.e., Egypt, Algeria, and South Africa) that share the same risk exposure from the provinces of Guangdong, Fujian and Beijing. Countries at moderate risk (i.e., Nigeria, Ethiopia, Sudan, Angola, Tanzania, Ghana, and Kenya) have variable capacity and high vulnerability. 

As the classic Health Belief Model (HBM) and other models for health psychology indicate, risk perceptions have been seen as one of key drivers of health behaviors [30]. In the HBM, perceived susceptibility, perceived severity, perceived benefits, perceived barriers, cues to action, and self-efficacy (individual beliefs in disaster preparedness) predict individual behavior [31].

### 1.2. Epidemic Hazard in Serbia

During the Crusades (1096–1291), the territory of the Republic of Serbia was affected by a leprosy epidemic that claimed several dozen human lives in the Balkans. Then, in the Middle Ages, the plague occurred on several occasions (1348, 1362, 1428, 1430 and 1438), with the most recent cases being recorded between 1836 and 1838 [32]. Although the borders in Balkans have often been moved and outbreaks of infectious diseases are not uncommon, over the last century three major outbreaks have been linked to the geographical area of the Republic of Serbia. Firstly, during the First World War, the Serbian army and public were greatly affected by typhus. The second was a smallpox epidemic of in Socialistic Federative Republic of Yugoslavia (SFRY) in 1972, “the biggest post-war outbreak in Europe”; and, lastly, the tularemia outbreaks during the 1990s [33].

The lack of time and resources to recover from the losses caused by the Balkan wars (1912–1913), the epidemics of typhoid, recurrent and especially freckled typhus (besides dysentery, measles, diphtheria, scarlet fever, smallpox, etc.), have consequently spread all over Serbia and caused losses that no other people and army suffered during the First World War [34]. World Health Organization (WHO) developed a major eradication plan for *Variola major* in 1967, and 10 years later the last case of smallpox was registered in Somalia. However, the last large outbreak in Europe occurred in 1972 in Yugoslavia, with 175 cases and 35 deaths [35,36]. Before eradication, the last case of smallpox in Serbia was recorded in 1930, with the last lethal consequence in 1926. Although 38,963 people lost their lives in smallpox epidemics between 1896 and 1910 for many years, medical personnel were not trained, prepared and experienced at the time of the outbreak of smallpox [33]. Nevertheless, despite organizational, technical and other weaknesses, the epidemic was quickly brought under control within four weeks after the detection of the first case by vaccination of whole SFRY population (about 18 million citizens) which began on 16 March, surveillance and movement restrictions [33].

Although the first human infection in SFRY was reported in 1947 and the disease occurred sporadically in Serbia, the first outbreak of tularemia in Serbia was recorded in late 1998 in the Sokobanja region. The epidemic lasted until 1999 and 2000, with oropharyngeal tularemia as the dominant form of the disease. Subsequently, minor tularemia outbreaks occurred in the district of Pčinja in 2010, Gazdin Han in 2014 and in 2015 in the natural foci of the disease—Stara planina, Suva planina, and Rtanj and Kopaonik mountains [33]. The recent history of the Republic of Serbia also records the flu epidemic in 2009/2010, at the time of pandemic. The population surveillance reported 190,563 (2.5%) of patients with clinically manifest flu symptoms between 26th week of 2009 and the 13th week of 2010. 6021 patients were hospitalized for influenza-like illnesses, among which influenza was laboratory confirmed at 553. Among those hospitalized, 137 deaths were reported (mortality rate—2.3%), 122 of which had influenza as the cause of death, and 15 died as a result of complications of chronic illness following influenza [37]. Although Ristanović [33] argued that smallpox epidemics, as well as other epidemics mentioned above, should have provided sufficient warning and improved preparedness for response, and rare studies have shown that this is not the case. In the context of general publics’ preparedness for epidemics, Cvetković et al. [13] have shown that “less than half of respondents know what epidemics are and how to protect themselves against epidemics”. Another interesting testimony about Serbia’s poor public knowledge of infectious diseases was found. 

The aim of this paper was to establish the level and impact of certain demographic characteristics of preparedness and preventive behavior in the event of a pandemic disaster caused by COVID-19 in Serbia.

## 2. Methods

The research was designed to identify adults’ preparedness behaviours during the epidemic of COVID-19 in Serbia, with the result that all participants were Serbian residents over 18 years of age. Due to the extraordinary circumstances of the COVID-19 pandemic, in particular the restriction of movement and the need for social distance, the data collection for this study had to be conducted via the on-line survey platform (google.doc). Namely, the study was conducted in April, 2020, only two weeks after the state of emergency was declared, ensuring that the researchers complied with movement restrictions and the need for social distancing. The participants were invited to complete the on-line questionnaire in their native language by using the snowball sampling method. 

### 2.1. Study Area

The Republic of Serbia is located at the crossroads of Central and South-Eastern Europe in the Southern Pannonian Plain and the central Balkans and covers an area of 88.499 km^2^. It borders Hungary to the north, Romania to the northeast, Bulgaria to the southeast, North Macedonia to the south, Montenegro to the southwest and Croatia and Bosnia and Herzegovina to the west. Also, the disputed territory of Kosovo in the south of the country, borders with Albania to the south-southwest. According to the 2011 census, there were 7,186,862 inhabitants in the Republic of Serbia, of whom 3,499,176 were male and 3,687,686 were female. Almost one third of population is concentrated in just 5% of the territory (the metropolitan area of Belgrade and regional centers as Novi Sad, Niš and Kragujevac), while more than 35% of the territory is well below the average population density (up to 50 inhabitants/km^2^). Over 1.7 million of Serbian residents were retired, 1.2 million were 65 years of age or older, more than 800,000 were officially unemployed, and more than 570,000 people had some kind of disability [38]. It is also important to note that Serbia is a country in transition, with the official position of the government being to become part of the European Union. 

### 2.2. Socio-Economic and Demographic Characteristics

As an initial invitation to participate in an online questionnaire was posted on social media and sent to the authors’ contacts and their contacts, the sample was convenient and not necessarily representative of the Serbian population. Of 975 respondents total, 76.6% were women and 23.4% men (women 51.3% and men 48.7% of the total country population). The average age of respondents was 27 years old and the most represented category was 18–28 years old (708; 72.6%) while the least elderly were 49–58 (51; 5.2%) (average age of the population 42.6 years (men 41.2 and women 43.9). From the sample, it appears that the majority (57.2%) had undergraduate educational degrees, and very few only completed primary school (0.3%) (with primary sch. 20.76%, high sch. 48.93%, junior college 4.51%, and undergraduate 10.59% of the population). In the household sample in a relationship people account for 39.4%, widow/widower 0.9%, and married rate of 13.8%. The interviewees selected also reflected different status of disability, with 7.7% of the individuals reporting some type of disability or chronically illness and 92.3% reporting lack of disability. In addition, the majority (44%) of respondents had monthly household income of more than 741$ (Table 1).

### 2.3. Questionnaire Design

The structured questionnaire was developed using close-ended and 5-point Likert scale questions (1 strongly disagree to 5 strongly agree). The first part of the questionnaire included the socio-demographic characteristics of the interviewees in order to assess the social background and gender of the respondents. Subsequent sections included issues questions related to *knowledge, preparedness, risk perception and information sources, and preventive measures for coronavirus disease* (Appendix A). Several published survey approaches were consulted [13,14,17,19,27,28,39] and adapted according to the circumstances of the COVID-19 pandemics in Serbia. A pilot pre-test of the questionnaire was conducted in Belgrade (central Serbia) in April 2020 with 45 people to test the comprehensibility and performance of the questionnaire via online systems. Our research was consistent with the Helsinki Declaration outlining the principles for socio-medical research involving human subjects. Participants provided informed consent to participate in the study. The research protocol was approved by the committees of scientific research group review board of Scientific-Professional Society for Disaster Risk Management, ID-04052020.

### 2.4. Analyses

Descriptive statistics were calculated for the demographic characteristics of the participants in this study. The analysis of variance (one-way ANOVA with Post hoc Tests-Supplementary), Chi-square test and the regression analysis were used to examine the relationship between the variables and preparedness scores of the participants. All tests were two-tailed, with a significance level of *p* < 0.05. Statistical analysis was performed using SPSS Statistics 17.0 (IBM SPSS Statistics, New York, NY, USA). The internal consistency of Likert scales for Preparedness Subscale (24 items) is good with a Cronbach’s alpha of 0.89, for Preventive Measurement Subscale (20) 0.87, Risk Perception Subscale (13 items) 0.83. 

## 3. Results

The results were based on knowledge, preparedness, risk perception and preventive measures for coronavirus disease. Starting from the abovementioned methodological framework, the results were handled into four categories: 

Firstly, we tested the central hypothesis that gender, educational level and age were predictive variables of citizen preparedness for a pandemic disaster. Multivariate regression analysis was used to determine the extent to which four scores of the subscales (knowledge, preparedness, risk perception, preventive measures) were associated with six socio-economic variables: gender, age, marital status, education level, disability, and monthly income. Analyses showed that the assumptions of normality, linearity, multicollinearity and homogeneity of variance had not been violated. 

The results of the multivariate regressions of preparedness subscale showed that the most important predictor is disability (β = 0.091), and explains 9.1% variance in preparedness [40]. The remaining variables (e.g., gender, age, marital status, income level) were not significantly affected by preparedness. This model (*R*^2^ = 0.17, Adj. *R*^2^ = 0.11, *F* = 2.73, *t* = 4.95, *p* < 0.01) with all mentioned independent variables explains the 11% variance of preparation (Table 2). 

Further analyses showed that the most important predictor of knowledge subscale was the level of education (β = −0.107), explaining 10.7% of the variance, followed by age (β = −0.081, 8.1%) (*R*^2^ = 0.27, Adj. *R*^2^ = 0.21, *F* = 4.43, *t* = 10.37, *p* < 0.01). This model (with all mentioned independent variables) explains the 21% variance of knowledge. The remaining variables did not have a significant impact on knowledge (Table 2).

The analyses showed that the most important predictor of risk perception was age (β = 0.134) with 13.4% of variance followed by education level (β = −0.060, 6%). This model with all of the defined independent variables explains the 19% variance of risk perception (*R*^2^ = 0.25, Adj. *R*^2^ = 0.19, *F* = 4.16, *t* = 16.47, *p* < 0.01). The remaining variables did not have significant effects on the perception of risk (Table 2).

The most important predictor in preventive measures was age (β = 0.196) which explains 19.6% of variance followed by the gender (β = 0.167, 16.7%). This model with all independent variables mentioned explains the 63% variance of preventive measures (*R*^2^ = 0.69, Adj. *R*^2^ = 0.63, *F* = 11.9, *t* = 10.07, *p* < 0.01). The remaining variables did not have significant effects on knowledge (Table 2).

### 3.1. Knowledge of Coronavirus Disease

In terms of basic knowledge, the respondents were offered statements and the possibility to “agree” or “disagree” (yes or no), when required, coded as 1 or 2, respectively. According to the results, 84.6% of respondents indicated that they had knowledge of epidemics, although 65.8% of them, when it comes to assessing their knowledge, confirmed zoonotic nature of coronavirus, which means that virus was transferred from animals to humans. Then, 71.4% of respondents said that at the end of 2019, COVID-19 virus occurred for the first time in human population in China. Most of the respondents (95.4%) thought coronavirus-related disease was infectious and 16.3% of the respondents stated that symptoms occur only after 14 days, while 91.7% of the respondents stated that symptoms occur within 0 to 28 days of infection/exposure to virus. When asked about symptoms occurring in infected persons, the majority of the respondents (98.2%) noted that common symptoms include fever, severe fatigue and dry cough. On the other hand, 68.3% of respondents believe that other symptoms include difficulty breathing, pain, diarrhoea, nausea and runny nose. 

The results of Chi-square test showed that there was no relationship between self-perceived knowledge of COVID-19 and the variables examined. Contrarily, the relationship between COVID-19 was found to be zoonotic disease and educational level. Specifically, the respondents with completed masters/doctoral studies (75.7%) were more likely to have the above assertion compared to the respondents who graduated from junior college (37%). In addition, in relation to the argument that the coronavirus appeared first in China, a statistically significant association with education was found in such a way that university level respondents (75.8%) agreed with the above-mention statement, mainly in relation to high-school level respondents (56.6%). A significant statistical correlation was found between this belief that COVID-19 was contagious and gender that women (96.4%) were more likely to agree with this statement than men (89.7%). A statistically significant correlation was observed between the opinion that the symptoms of COVID-19 would occur only after 14 days and the majority of respondents aged 49 to 58 years of age (50%) support this statement. On the other hand, the statement that symptoms may occur between 0 and 28 days after exposure to COVID-19 showed significant statistical association with the level of education and age of respondents. Elderly (55%) and high-school respondents (18.3%) agree to a greater extent than younger respondents (16%) and university graduates (15%). Finally, it was also found that agreement with the statement that other symptoms include difficulty breathing, pain, diarrhoea, nausea and runny nose were associated with educational level and age. Respondents with a university degree (83.8%) and elderly respondents (73.3%) support this statement to greater extent than those with high-school degree (71.4%) and younger respondents (65%) (Table 3).

### 3.2. Preparedness for Coronavirus Disease

Considering the importance of citizens’ preparedness for responding to epidemic-induced disasters, the respondents were asked to rate the level of preparedness for the coronavirus epidemic on a scale of 1 to 5. On this occasion, the following descriptive results were obtained: individual preparedness (X = 3.56); household preparedness (X = 3.58); local government preparedness (X = 2.76); state preparedness (X = 3.12); individual knowledge for responding (X = 3.41); enough training for responding (X = 3.11); food supplies (X = 3.42); protective equipment (X = 3.09); response plans (X = 3.14); household knowledge (X = 3.49), training (X = 3.26), plans (X = 2.56); local community knowledge (X = 2.72), plans (X = 2.56), training (X = 3.34) and supplies (X = 2.79); state knowledge (X = 3.28), trainings (X = 3.27), supplies (X = 3.58), plans (X = 3.18); first responders preparedness (X = 3.79); healthcare institutions—knowledge (X = 3.54) and their supplies for responding (X = 2.53) (Table 4). 

When the association between gender and different variables of preparedness to respond to disasters caused by COVID-19 was analyzed, it was found that women rate local communities and states at a higher level of preparedness, while level of training and food supply is ranked lower. Significant statistical association was found between the educational level and different variables of preparedness, with respondents who received a high-school degree noting highest scores for household, local community, members of the emergency services preparedness. Furthermore, respondents with completed masters or doctoral studies expressed the highest rates of self-assesed individual preparedness, training for adequate response, household-level plans. Besides that, junior college respondents noted the highest scores for state preparedness and the possession of sufficient food supplies. Finally, it was confirmed that university-level respondents reported the highest level of knowledge for adequate response and the possession of sufficient protective equipment (Table 4).

On the other hand, when the age of respondents was analyzed, it was determined that respondents aged 29 to 38 years have reported the highest rates of ownership of sufficient food supplies. Also, it is found that respondents aged 39 to 48 report highest scores for individual preparedness, the level of knowledge required for adequate response, and the level of household response knowledge. Preparedness of first responders was the best evaluated by the respondents aged 18 to 29. On the other hand, respondents aged 58 or older years assessed the level of disaster response training in question and having plans (Table 4).

### 3.3. Risk Perception and Informing of Coronavirus Disease

Starting with the importance of informing citizens on a timely basis of current risks regarding the coronavirus epidemic, respondents were asked to rate between 1 and 5 different ways of informing on a Likert scale from. The results of the survey showed that citizens get the most information online (X = 4.23), then TV (X = 3.88), family members (X = 3.19), friends (X = 3.10), members of first respondent services (X = 2.45), scientific journals (X = 2.33), local community (X = 2.22), newspapers (X = 2.02), chosen physician (X = 1.98), government organizations (X = 1.86), radio (X = 1.74). As a result, in order to obtain needed information about the current status of the coronavirus epidemic, citizens the most frequently use the internet and radio were the least used. In relation to the source of information, the most reliable information was obtained by experts (epidemiologists, other physicians and experts in the field of disaster risk management) (X = 4.35), websites of international health organizations (WHO, Centers for Disease Control and Prevention, etc.) (X = 3.48), addresses of official state governance structures (X = 3.87), social networks (X = 3.10), communications from educational institutions (X = 2.74), national Institute of Public Health website (X = 2.70), websites of local medical institutions (X = 2.47) (Table 5).

By examining the relationship between gender and various variables of information sources, it was found that men are mostly informed via television. On the other hand, women are mostly informed through the websites of local medical institutions, and most believe the information provided through the addresses of the officials and experts and educational institutions (Table 5). In addition, it was found that the majority of men reported that infected individuals develop mild to moderate respiratory symptoms, from which they will recover quickly without special treatment. Women, on the other hand, were mostly afraid of the health of family members and fear of the major economic consequences of the disaster (Table 5).

As regards the association between education and various information variables, it was found that respondents with high school graduation are mostly informed by television, radio, websites of local health institutions, addresses of officials, friends, local communities, chosen physicians, members of first-time respondents services (Table 5). Respondents with a university degree were found to be mostly informed through non-governmental organizations, educational institutions. Then, respondents with a masters/doctoral degree are mostly informed through the newspaper, scientific journals, family members (Table 5).

In the context of respondents age, those aged 18 to 29 are more informed through television to the greater extent, have the highest level of trust in information obtained through addressing of the officials. Additionally, respondents aged 29 to 38 are mostly be informed by friends. On the contrary, respondents aged 39 to 48 are mostly informed through the internet, scientific journals, websites of local health institutions, members of first responders services, educational institutions. In addition, respondents aged 58 years and older are most frequently informed via the radio and state that they are in the higher risk of epidemics due to their place of residence (Table 5).

In the following, examining of the different dimensions of risk perception we obtained the following attitude ratings: (a) The likelihood that I will be infected with the virus is low (X = 3.00); (b) The majority of people who become infected will develop mild to moderate respiratory symptoms, from which they will recover without the need for special treatment (X = 3.43); (c) Elderly people and those with chronic illnesses (e.g., respiratory, cardiovascular, diabetes, immunity, etc.) are at the highest risk of developing severely treatable diseases and are at high risk of death (X = 4.52); (d) I believe that even if I do get infected I will not develop serious health problems (X = 3.66); (e) Although they are not at risk of developing severe illnesses, I find it good that children do not go to kindergartens and schools (X = 4.71); (f) I think that I am in a higher risk of being infected with the virus because of where I live (X = 2.90); (g) I believe that because of the epidemic and the state of emergency declared, I am at risk of losing my job and source of income (X = 2.72); (h) I believe that through my behavior (taking preventive measures) I can prevent the spread of virus (X = 4.51); (i) I feel that I have a responsibility to do what I can to protect people at risk (X = 4.58); (j) we will not be able to stop the spread of the virus by following the introduced measures (X = 2.30); (k) I think I have all the information I need to critically review the situation in our country caused by epidemic and decide what to do (X = 3.60); (l) I fear for the health of family members (X = 4.11); (m) I fear that the economic consequences of the epidemic will be great for society (X = 4.28); (n) I’m afraid the restriction on movement will prevent me from meeting my needs (take medication, taking money, buy groceries) (X = 2.62).

In relation to the association between education and various risk assessment factors, respondents with a high school diploma rate the highest statement that protective actions will avoid the spread of the virus, statement of obligations for taking measures to protect people at-risk, and the statement that they are afraid of limitations. Respondents with a university degree were found to have mostly assessed the level of risk of being unemployed, and stated that they needed information for critical evaluation of the situation (Table 6).

In the context of respondents’ age, those aged 18 to 29 stated that their behaviour, which was taking preventive measures, could stop spreading of the COVID-19, and that even if they were infected, they wouldn’t develop serious health problems. Respondents aged 18 to 29 showed the highest scores for assessment of first respondents services preparedness, stated that the majority of infected persons would develop mild to moderate respiratory symptoms and had a responsibility to do what they could to protect others (Table 6).

### 3.4. Preventive Measures for Coronavirus Disease

Despite the high importance of preventive measures to prevent the further spread of the epidemic, different claims on the Likert scale were made to the respondents, and the following results were obtained. Wash hands with soap and water for 20 s (X = 4.78), use disinfectants to maintain the hygiene of the room (X = 4.47), wear protective mask (X = 3.25), wear protective gloves (X = 3.16), do not touch the face (eyes, nose, mouth) (X = 3.95), do not shake hands with acquaintances (X = 4.65), do not hug with family members, friends, acquaintances (X = 4.29), do not kiss on the cheek with family members, friends, acquaintances (X = 4.31), keep a recommended distance of 2 m from other people (X = 4.15), respect restrictions of movement in public places (X = 4.88); avoid contacts with people over 65 (X = 4.63), do not see family members with whom they do not live in the same household, friends, acquaintances (X = 4.23), using a disinfectant to clean my shoes and clothes in which I went to make my purchases, to the workplace (X = 3.66), disinfect the pet’s paws upon returning from the walk (X = 3.30), move pets out of the living space and no longer have contact with them (X = 2.71), to make a plan with the members of my household about the modes of isolation if necessary (X = 2.85), Preparing a diet plan and necessary food with household members (X = 3.36), having supplies of groceries needed for 3 days of isolation (X = 3.36), having supplies of groceries needed for 3–7 days of isolation (X = 4.27), having supplies of groceries for a month and longer (X = 3.63). Respondents were also asked whether they had been engaged in any form of assistance prior to the epidemic of the coronavirus, and it was found that 68.6% of respondents were not engaged before the epidemic, while 29.2% of respondents were involved during the epidemic before or at the time of research.

Examining the relationship between gender and different preventive measures variables, it was found that women use soaps to a greater extent, disinfect clothing and footwear, wear protective mask, do not touch their face, do not shake hand with acquaintances, avoid contact with the elderly, do not see family members, disinfect pet’s paws. On the other hand, males only use disinfectants more (Table 7).

In relation to the association between educational level and various variables of risk perception, it was found that respondents with high school respondents were most likely commonly made isolation plans for their households. Furthermore, respondents with junior college degrees avoid hugging, and kissing with family members, friends and acquaintances and use disinfectants for clothes and shoes to the highest extent. Respondents with a university degree avoid contacts with pets to the highest degree. Finally, respondents with completed masters/doctoral studies to the highest degree avoid shaking hands, maintaining recommended distance, having food supplies for months or more (Table 7).

When age of respondents was analyzed, it is found that respondents aged 29 to 38 years to the highest extent respect movement restrictions, have food supplies for a month or more, and avoid contact with the elderly. Respondents aged 39 to 48, on the other hand, most commonly avoid hugging, and kissing with family members, friends and acquaintances, then carry out disinfection, and avoid contacts in general. Additionally, respondents aged 49 to 58 to the highest degree, avoid shaking hands, they maintain recommended distance, disinfect their pets’ paws and make dietary plans (Table 7).

## 4. Discussion

The results of this study showed that the respondents had knowledge gaps three weeks after the state of emergency was declared. It was not expected that 84.6% of respondents would state that they had knowledge of epidemics and how to prevent them, since 43.1% of respondents agreed, 26.6% were not sure and 24.7% disagreed with the same statement in 2015 [13]. This finding is even more confusing when we look at the results of perceived sufficient knowledge and training to respond properly to epidemic emergencies. Respondents expressed the highest degree of understanding that COVID-19 is a contagious disease and that most commonly manifests symptoms such as fever, fatigue and dry cough. In addition, most of them even recognized that symptoms could manifest within 28 days of exposure to virus at a time when the possibility of incubation period longer than 14 days was newly introduced to the public. In contrast to health information, respondents were less receptive to “technical” information on the novel coronavirus, its nature and first occurrence. These findings could be the result of greater monitoring of health experts broadcasted on television and online every day, focusing on health information. Additionally, at the time of this research respondents could find many “conspiracy theories” online, even in national media and social networks, which could led to theirs suspicion and lower level of agreement with these statements. Nevertheless, this general publics’ more responsiveness to health information in the event of an epidemic should be considered when informational campaigns are launched, since this finding is congruent with some of identified publics’ need for information in the case of bioterrorism—infection, transmission prevention and exposure detection [41].

The strongest predictor of knowledge was education level, that is, the more educated a person was, the more knowledge they have of effective measures to prevent infection and disease with COVID-19. Previous studies in Serbia have also shown that the educational level (university and high school graduated) is correlated with higher level of epidemiological knowledge and a proper respond [13]. It is interesting to note that respondents with high school degree and younger respondents have been more likely to recognize that COVID-19 symptoms could occur within 28 days period and other symptoms possible symptoms. These categories have reported that they mostly inform trough television broadcasts and experts’ addressing’s, thus leading to the conclusion that informing of the Serbian public about the novel coronavirus had its strengths.

Gender has also been found to be predictive of knowledge, with women being more likely to agree with the fact that COVID-19 is a contagious disease. Bearing in mind that women in Serbia have already demonstrated a higher level of knowledge about epidemics [13,42] the question arises as to whether this finding is biased due to the sampling method. However, previous studies have found conflicting results. Some of them discovered males [39,43] and elderly respondents to be more knowledgeable [43] while others found females to be more knowledgeable [44].

Our respondents consider household and individual preparedness to be higher than the state and local community preparedness. This is not surprising since social, economic, functional, institutional and political factors have had an impact on successful emergency management [45] and Serbian citizens have been protesting for months before epidemic claiming that they had enough of problems in affected areas. The fact that government has changed its opinion about epidemic severity for its population, as well as for other officials, dealing with catastrophic scenarios and equipment shortages (of respirators especially), has most certainly reduced the perception of preparedness. Additionally, the perceived knowledge of healthcare institutions and response supplies are consistent with the idea that coronaviruses are not new, although COVID-19 is, to our healthcare professionals and lack of protective equipment and disinfectants in the country at the time of research. 

Multivariate regression results demonstrated that some type of disability (invalidity and/or chronically disease) is the most important predictor of individual preparedness, although those with higher educational levels and between 38 and 49 years of age were most certain of their preparedness. In previous epidemic preparedness study in Serbia, males with higher educational level and non-disability perceived their individual preparedness higher than other respondents [13]. On the other hand, both male and younger respondents had higher scores on household and community preparedness for earthquakes in Serbia [46]. In the case of floods and fires, males in Serbia were more confident in their ability to cope and perceived greater individual and household preparedness [42,47]. Males from Belgrade were most confident in individual, household and community preparedness for terrorist attacks, and respondents with high school degree in household and community preparedness. Various age categories demonstrated different terrorism preparedness perception: younger respondents perceived household preparedness greatly, middle aged individual, while elderly believed most in community preparedness [48,49]. Given that perception of preparedness is significantly different in the event of various disasters, this study finding could be explained by proactive approach of more educated and middle aged respondents towards information gathering, since they have mostly reported internet, websites of medical institutions, scientific journals, etc. as sources of information and believed that they have information needed to critically evaluate the situation they are in. The higher preparedness level of local community and state was perceived by those with high school and junior college degrees, respectively, and women for both. These findings could be the result of frequent television monitoring for respondents with high school degree and higher levels of trust among women towards experts which were addressing to the public on a daily basis. 

Although television and continuous broadcasting of experts’ addresses were among the most frequently selected information sources, the results of our study demonstrated that most citizens received most of their information about novel coronavirus online via the internet and used radio only infrequently. Women were more engaged in information seeking activities than men, as was also found in the case of the A (H1N1) epidemic [26]. The internet preference over television as information source was previously identified in the research on Serbian publics’ opinion about bioterrorism [15]. Obtained results could be explained by the fact that since all respondents were recruited online, they represent preselected internet users. Nevertheless, Serbian public expressed an increasing tendency to turn away from television and, especially, from radio, and to the internet and social networks with only 9.4% of participants, with media confidence on average ranging from 23.3% among the elderly to 5.3% among the youngest participants [50]. Another one research showed that 61% of Serbian citizens do not trust the media [51] and that 65% of Serbian citizen use the internet in 2015 [52]. This explanation should be extended to the context of Serbian media environment, which was found to be neglect progress in the area of freedom of expression, threats, intimidation, and to show violence against journalists, nonexistent transparency of media ownership, partial implementation of media regulations and insufficient European Commission Regulatory Body for Electronic Media (REM), 2016. Additionally, the public broadcaster Radio Television of Serbia (RTS) tends to be a service of the ruling political party [53], N1 television lost its national frequency prior the epidemics since they were one of few who opposed the ruling party, and while a few remaining national broadcasters are predominately turned towards entertainment and lifestyle programming. It is important to note that the independent journalist Ana Lalić was arrested on the night of 1 April 2020 for releasing the story about significant shortage of equipment in the Vojvodina Clinical Center [54] that continues to claim to have originated from reliable sources and in line with the principles of investigative journalism.

In relation to the source of information, the most trusted information was obtained from experts such as epidemiologists, other physicians and experts in the field of disaster risk management), websites of international health organizations, internet links to official state governance structures, communications from educational institutions, websites of the Institute of Public Health and local medical institutions such as schools of medicine. These results are further consistent with Serbian publics’ trust in institutions, with educational (61%) and healthcare (57%) institution viewed as trustworthy [51]. Kwok et al. [28] also reported that doctors (84%), thus the experts, are the most trusted sources of information, followed by television (57%) and newspapers (54%). Hong Kong citizens reported social platforms (94%) and official or non-official websites (90%) as the most common sources of information, although they were considered as “only 16–23% of the respondents to be reliable or very reliable”.

Respondents were worried about the health of their family members and thought that kindergartens and schools should be closed even if children are not at-risk. Although they have not recognized their personal risk as high, they have understood that elderly citizens and those with chronic diseases are in great risk and that they have the responsibility to take actions in their power to protect them. The fact that males expressed a lower perception of their personal risk for becoming ill is consistent with understanding that females in Serbia are the ones most likely to fear because they have been taught that they should be protected [49]. In case of H1N1 influenza epidemic, females were also identified as having higher personal susceptibility higher [26,27].

Younger respondents were aware that probably they likely to develop serious health problems but that their behavior could stop spreading of the virus. This was the focal thesis of all the officials and experts addressed by the broadcasted, thus our results without a doubt testify to their effectiveness. Even the lowest level of agreement among elderly respondents that they are at-risk because of their place of residence is consistent with this claim. Place of residence, in terms of higher number of cases was found to be significant during A (H1N1) epidemic in the US [26]. Citizens of Hong Kong, on the other hand, perceived their susceptibility (89%) and severity (97%) as high and 97% of survey respondents were worried about COVID-19. Namely, most of the respondents thought that were very likely to likely to get infected (89%) and that symptoms of COVID-19 are very serious or serious (97%), while only 15% of them thought that they are likely to be cured in infected and 18% that they would survive. Since the respondents in this sample were mostly young (80% of them were between 18 and 44 years of age [28] it could be concluded that Serbian sample had better understanding of COVID-19 health implications. 

Among respondents, fear about economic impact of the epidemic was common, although they were not as much worried about losing their jobs. This finding is somewhat contrasting until we consider that most of the respondents were younger citizens and students, and that fear of job losses was the highest among university-level respondents, thus employed subsample. It also points out the fact that younger respondents are aware of the potential impact of the epidemic on society, especially since the unemployment and economic problems were identified as the biggest problems (41%) of Serbian youth [55].

Respondents reported significant behavioral changes in hand hygiene, social distancing (recommended distance, movement restriction, avoiding elderly and family members). On the other hand, significant lack of protective masks and gloves, as well as disinfectants, could be considered as a cause of poorer adherence to these measures. This conclusion is further supported by the respondents’ perception of having enough of required protective equipment since lack of it could have an impact on their usage. Additionally, the efficacy of protective masks for healthy people was advocated to be low by WHO and national health experts at the time of this research. However, the respondents have perceived that preventive measures were effective. Hong Kong citizens also reported significant behavioral changes when it comes to hygiene practices, but lower adoption of social distancing measures, although they have considered them useful in preventing COVID-19 [28]. The H1N1 [19,23] and SARS [17] epidemic in Hong Kong were also seen prevalent adoption of hygiene practices. On the other hand, UK residents have reported low levels of precautionary measures during H1N1 epidemic, with 62% of respondents reporting no change in the frequency of hand washing (72%), cleaning or disinfecting (83%) or discussing plans with people who should take care of them if they get ill (83%) [17]. Finally, all countries should urgently take all necessary measures to slow further spread and prevent their health systems from becoming overwhelmed by seriously ill patients with COVID-19. Also, respondents have reported significant attention to nutrition plans and food supplies, despite frequent assurances from officials that food supplies are not a problem, or because of them? Respondents with masters or doctoral degree were most likely to report stockpiling more than a month’s worth of food supplies in their homes even though it has previously been shown that educational levels indicate a wider range of information sources and perceived individual and household preparedness. Until the declaration of the state of emergency, it should be remembered that the products were “disappearing” from the raffles, in particular wheat, yeast, oil and toilet paper. While this may be a legacy of the past (inflation, civil war and NATO bombing), it should be further examined whether more educated citizens acted on the basis of information they’ve gathered (and critical evaluation of them) or on general distrust of statements made by officials. 

Additionally, age was demonstrated to be the most important predictor of preventive measures, although gender and educational level were also significantly predictive to adopting precautionary measures. It is interesting that younger respondents reported the highest level of restriction on movement, since it could be the result of high reception of messages from television and experts, but could also be the result of the penalties imposed. In the sense of Serbian economy, it is important to note that the average net salary in February 2020 was 58,132.00 RSD ($535.26) while the amount of the misdemeanor sentence for non-compliance with the travel ban was set at 50,000.00 to 150,000.00 dinars or between $460.38 and $1381.15 [38]. Thus, perception of risk shouldn’t be seen as motivator for the adoption of respect for the prohibition of movement among respondents, given that personal risk was not perceived as high and that the penalties mentioned were determined after the motion restriction recommendation was not followed. Additionally, for the past few weeks political opposition to ruling party and civil activist movements accused the government of dictatorship and contempt of the Constitution of the Republic of Serbia. 

Gender differences in adoption of preventive measures could be explained by their traditional roles in Serbia, with women more concentrated on family and/or household care, as can be seen in the study of earthquake preparedness study [46]. Females in our sample are the most likely to disinfect their home, clothing, and adopt social distancing to a greater extent than men, who only used disinfectants more for hand hygiene. Household care and behaviours were also demonstrated in the flood preparedness study in Serbia, as well as willingness to help flood victims [55]. Female gender was also found to be predictive of better adoption of precautionary measures in Hong Kong during COVID-19 epidemic as a public health emergency of international concern [28] and Korean study of influenza epidemic [27]. This was the case with females in Hong Kong during the SARS epidemic, but old age and higher levels of education were also more likely to take comprehensive precautionary measures to prevent infection [17]. On the other hand, being male and of old age was predictive of better preventive practice in the case of Dengue epidemic study in Wah Cantt [43].

As regards the association between educational level and various variables of preventive measures, it was found that high school respondents most commonly planned for their homes to be insulated, respondents with a junior college education avoided gestures of affection such as hugging and kissing family members, friends and acquaintances and were more likely to use disinfectants when cleaning their clothes. It should also be remembered that for weeks prior the epidemics Serbian citizens bought a large quantity of Asepsol (0.1%, 1% and/or 5% solution of ADBAC/BKC (C_12_–C_16_) and gel-based hand sanitizers only to be told during the state of emergency that only products containing 70% alcohol and those based on chlorine had a protective effect. Respondents with a university degree were most likely to avoid contacts with pets. Finally, respondents with masters or doctoral degrees were most likely to avoid shaking hands, maintain recommended social distances, and store more than a month’s worth of food supplies in their homes. On the other hand, Ramzan et al. [42] have found that the level of educational had no association with preventive behavior of their respondents. The proportion of volunteers in the survey sample is slightly higher than the findings of previous research on youth volunteerism (2007—20%; 2009—15%; 2011—20%; and 2012: 21%) with the most significant motivational factor helping those in need [55].

The limitations of our study include: (1) potential bias in selecting study subjects to complete questionnaires, (2) no study participants had any experience with a “real life” global pandemic emergencies, (3) insufficiently representative sample of respondents, (4) the timing of the study (the research was conducted in first phase, but the COVID-19 pandemic is still ongoing, and this may also have an impact on the study), (5) in particular the mental state of the respondents due to the overall situation etc.

## 5. Conclusions

The risk to communities with continued widespread transmission depends on the characteristics of the virus, including how well it spreads among people; the severity of resulting illness; and the medical or other measures available to control the impact of the virus (for example, vaccines or drugs that can treat the illness) and the relative success of these. In the absence of vaccines or medications, non-pharmaceutical interventions become the most important response strategy based on citizen preparedness measures such as person-to-person distancing, mask-wearing, isolation and good personal hygiene (hand-washing)—all of which have been demonstrated to reduce the impact of this seemingly unstoppable globally spreading natural disaster. The possibility that the COVID-19 virus could behave differently from previous coronavirus outbreaks in 2003 and 2015 requires new research studies, assessments, and plans to address a novel virus with high transmission in the community and severe morbidity and mortality. 

The study described in this paper deals with the critical initial steps in the process of preparing for a pandemic disease disaster, i.e., determining the general knowledge of citizens in Serbia regarding their level of knowledge regarding the general threat that novel coronavirus 2019 poses to both themselves and the wider community, risk perception, risk management and the recommended preventive approach. The results of this study demonstrated the importance of knowledge, preparedness, risk perception and preventive measures to effectively respond to a COVID-19 outbreak. We tested the central hypothesis that gender, educational level and age are predictive variables for stronger citizen preparedness for a pandemic disaster. 

Results of our survey indicate that there are major differences in the Serbian public’s perception of risks presented by pandemic Novel Coronavirus 2019 particularly their general knowledge *vis-à-vis* the general threat that novel coronavirus 2019 presents to both themselves and the community at large, risk perception, risk management and recommended preventive measures to take to decrease the possibility of becoming ill or dying from COVID-19. The study described in this paper addresses critical initial steps in the process of local and national preparation for a pandemic disease disaster. We encourage emergency management agencies in Serbia to use the differences in public perception of risks identified in our study to develop enhanced anti-pandemic disease preparedness measures by promotion of behavioural changes that go hand in hand with the of adoption of improved risk management decision-making procedures. 

All cities and towns in Serbia need to have disaster plans that are tailored to specific scenarios and locations, not preconceived generalized plans. Airport plane crashes, stadium catastrophes, and remote mass transit accidents are all very different from those caused by deadly infectious microorganisms such as COVID-19 and require different responses. Communications need to be standardized and supported. Triage needs to be thought through more clearly. Future research would benefit from a more epidemiological approach (e.g., case-control and cohort studies) to identify risk factors for poor community responses to communicable disease disasters such as epidemics, “before and after” studies looking at a population in Serbia that has been affected by infectious disease disasters such as coronavirus pandemics, and studies using currently validated modelling and simulation methods.

## Figures and Tables

**Table 1:** Basic socio-economic and demographic information of respondents (n = 975).

Variable	Category	Frequency (%)	Variable	Category	Frequency (%)
Gender	Male	228 (23.4)	Disability	Yes	75 (7.7)
	Female	747 (76.6)		No	900 (92.3)
Age	18–28	708 (72.6)	Monthly income	Up to 275$	96 (9.8)
	29–38	126 (12.9)		275 to 465$	210 (21.5)
	39–48	90 (9.2)		466 to 740$	240 (24.6)
	49–58	51 (5.2)		Over 741$	429 (44)
Marital status	Single	423 (43.4)	Education	Primary sch.	3 (0.3)
	In a relationship	384 (39.4)		High-school	240 (24.6)
	Married	135 (13.8)		Junior college	63 (6.5)
	Divorced	21 (2.2)		Undergraduate	558 (57.2)
	Widow	9 (0.9)		Master/doctorate	108 (11.0)
		TOTAL			975 (100)

**Table 2:** Results of a multivariate regression analysis concerning subscales (preparedness, knowledge, risk perception, preventive measures) for coronavirus disease (n = 975).

Predictor Variable	Preparedness			Knowledge			Risk Perception			Preventive Measures		
	*B*	*SE*	*β*	*B*	*SE*	*β*	*B*	*SE*	*β*	*B*	*SE*	*β*
Gender	1.35	0.71	0.063	0.136	0.128	0.035	0.260	0.397	0.022	4.88	0.949	0.167 **
Age	0.434	0.707	0.021	−0.297	0.127	−0.081 *	1.52	0.395	0.134 **	5.46	0.944	0.196 **
Marital status	−0.706	0.622	−0.039	−0.200	0.112	−0.061	−0.032	0.348	−0.003	0.507	0.830	0.020
Education level	−0.728	0.674	−0.035	−0.405	0.121	−0.107 **	−0.705	0.377	−0.060 *	1.15	0.899	0.040
Disability	14.9	5.29	0.091 *	−0.543	0.953	−0.018	−1.69	2.95	−0.018	−6.13	7.05	−0.027
Monthly income	1.26	0.990	0.042	0.212	0.178	0.039	0.886	0.553	0.052	2.74	1.32	0.066
Adjusted R^2^	0.11			0.21			0.19			0.63		

* *p* ≤ 0.05; ** *p* ≤ 0.01; B: unstandardized (B) coefficients; SE: std. error; β: standardized (β) coefficients. Note: males, young, single-headed households, secondary-school respondents, low income and disabled people have been coded as 0; 1 has been assigned otherwise.

**Table 3:** Chi-square test results between gender, education level, age variables and knowledge of COVID-19.

Variable	Gender		Education Level		Age	
	Sig.(2-Tailed)	*X* ^2^	Sig. (2-Tailed)	*X* ^2^	Sig. (2-Tailed)	*X* ^2^
1. Self-perceived knowledge	0.169	0.287	2.62	0.134	2.59	0.065
2. The corona is zoonotic	2.01	0.157	17.28	0.002 *	5.32	0.256
3. First time in China	0.396	0.529	26.16	0.000 **	9.01	0.064
4. The disease is contagious	7.27	0.007 *	6.33	0.176	8.02	0.054
5. Symp. manifest after 14 days	0.733	0.392	8.62	0.071	13.57	0.009 *
6. Symp. manifest up to 28 days	3.62	0.067	38.05	0.000 **	25.40	0.000 **
7. Symp.: fever, fatigue and etc.	1.01	0.314	5.85	0.210	4.71	0.318
8. Other symptoms	3.97	0.054	18.01	0.001 **	29.33	0.000 **

* *p* ≤ 0.05; ** *p* ≤ 0.01.

**Table 4:** One-way ANOVA results between gender, education level, and age variables and preparedness for coronavirus disease.

Variable	Mean	Std. Deviation	Gender		Education Level		Age	
			*F*	*p*	*F*	*p*	*F*	*p*
9. Individual preparedness	3.56	0.89	0.179	0.673	6.64	0.000 **	6.89	0.000 **
10. Household preparedness	3.58	0.93	1.31	0.252	3.44	0.008 *	2.14	0.094
11. Community preparedness	2.76	1.03	16.87	0.000 **	5.11	0.000 **	4.73	0.003 *
12. State preparedness	3.12	1.04	12.78	0.000 **	4.60	0.001 **	2.62	0.049 *
13. Enough personal knowledge	3.41	0.96	1.31	0.252	7.64	0.000 **	10.651	0.000 **
14. Enough personal training	3.11	1.07	3.98	0.047 *	6.98	0.000 **	10.66	0.000 **
15. Enough food supplies	3.42	1.13	7.06	0.008 *	8.95	0.000 **	14. 663	0.000 **
16. Enough of req. prot. equipment	3.09	1.24	2.70	0.100	3.69	0.005 *	1.87	0.132
17. Personal response plans	3.14	1.07	0.716	0.398	10.95	0.000 **	5.66	0.001 **
18. Enough household knowledge	3.49	0.96	0.971	0.325	8.22	0.000 **	5.96	0.001 **
30. First responders preparedness	3.79	1.02	3.41	0.065	4.87	0.001 **	9.22	0.000 **

* *p* ≤ 0.05; ** *p* ≤ 0.01.

**Table 5:** One-way ANOVA results of information sources of citizens and gender, education level, and age the variables on the risk perception of coronavirus disease.

Variable	Mean	Std. Deviation	Gender		Education Level		Age	
			*F*	*p*	*F*	*p*	*F*	*p*
33. Television	3.88	1.17	39.09	0.000 **	3.76	0.005 *	8.93	0.000 **
34. Radio	1.74	1.08	0.055	0.814	7.31	0.000 **	6.46	0.000 **
35. Newspaper	2.02	1.31	2.07	0.064	6.69	0.000 **	1.13	0.045
36. Internet	4.23	0.98	0.090	0.764	1.34	0.251	7.68	0.000 **
37. Scientific journal	2.33	1.44	0.036	0.849	4.18	0.002 *	5.11	0.001 **
38. Local medical website	2.47	1.37	23.26	0.000 **	7.55	0.000 **	5.25	0.001 **
41. Addressing of a statesman	3.87	1.23	103.23	0.000 **	5.04	0.011 *	17.20	0.000 **
42. Addressing of an expert	4.35	0.84	53.10	0.000 **	1.83	0.052	3.49	0.015
43. The social network	3.10	1.41	2.45	0.118	1.55	0.185	1.57	0.195
44. Family members	3.19	1.24	0.249	0.618	7.46	0.000 **	2.26	0.079
45. Friends	3.10	1.22	1.20	0.272	5.72	0.000 **	11.23	0.000 **
46. Local community	2.22	1.23	0.560	0.454	4.65	0.001 **	0.218	0.884
47. Chosen physician	1.98	1.30	814	0.314	8.27	0.000 **	2.61	0.050
48. First responders	2.45	1.38	0.184	668	6.37	0.000 **	14.75	0.000 **
49. Non-government org.	1.86	1.10	0.853	0.356	8.96	0.000 **	3.43	0.017
50. Educational institutions	2.74	1.39	6.47	0.11 *	14.33	0.000 **	19.00	0.000 **

* *p* ≤ 0.05; ** *p* ≤ 0.01.

**Table 6:** One-way ANOVA results of psychological behaviours of citizens and the variables of gender, education level, and age on the risk perception of coronavirus disease.

Variable	Mean	Std. Dev.	Gender		Education Level		Age	
			*F*	*p*	*F*	*p*	*F*	*p*
51. The likelihood of infect.	3.00	1.12	0.024	0.877	2.52	0.055	6.32	0.000 **
52. Respiratory problems	3.43	1.09	13.01	0.000 **	5.59	0.068	5.23	0.001 **
53. Most severe symptoms	4.52	0.73	3.08	0.079	1.63	0.164	2.44	0.062
54. Serious health	3.66	1.03	4.79	0.029	5.06	0.000 **	1.86	0.134
55. Kindergarten or school	4.71	0.73	0.004	0.947	2.31	0.056	2.13	0.057
56. A place of greater risk	2.90	1.49	0.518	0.475	2.84	0.051	37.2	0.000 **
57. Losing my job	2.72	1.48	2.10	0.147	2.78	0.026 *	1.86	0.067
58. Prevent behavior	4.51	0.77	0.121	0.728	4.24	0.002 *	9.41	0.000 **
59. The responsibility	4.58	0.75	1.05	0.305	4.34	0.002 *	7.33	0.000 **
60. Respecting measures	2.30	1.33	0.484	0.487	9.02	0.000 **	1.43	0.231
61. Information critically	3.60	1.03	0.925	0.336	2.71	0.029 *	1.53	0.205
62. I’m afraid of health	4.11	1.02	20.24	0.000 **	2.21	0.065	1.51	0.208
63. Econ. consequences	4.28	0.94	24.05	0.000 **	1.80	0.127	1.90	0.064
64. Fear of restrictions	2.62	1.31	0.147	0.701	3.17	0.013 *	2.28	0.078

* *p* ≤ 0.05; ** *p* ≤ 0.01.

**Table 7:** One-way ANOVA results of different groups of independent variables and variables on the preventive measures for coronavirus diseases.

Variable	Mean	SD	Gender		Educ. Level		Age	
			*F*	*p*	*F*	*p*	*F*	*p*
64. I wash my hands with soap	4.78	0.54	40.14	0.000 **	0.31	0.868	1.00	0.392
65. I’m disinfecting my hands	4.47	0.87	39.95	0.000 **	2.41	0.057	0.416	0.742
66. I wear a protective mask	3.25	1.6	13.92	0.000 **	1.97	0.097	1.00	0.389
67. I wear protective gloves	3.16	1.67	18.16	0.000 **	1.72	0.142	2.25	0.081
68. I don’t touch my face	3.95	1.17	26.58	0.000 **	2.16	0.071	1.29	0.275
69. I don’t shake hands with acquaintances	4.65	0.85	14.78	0.000 **	4.44	0.001 *	7.13	0.000 **
70. I’m not hugging others	4.29	1.17	1.97	0.160	6.07	0.000 **	4.40	0.004 *
71. I do not kiss others	4.31	1.22	2.86	0.091	7.83	0.000 **	8.06	0.000 **
72. Maintaining recommended distance	4.15	1.12	0.009	0.926	7.92	0.000 **	18.57	0.000 **
73. I respect movement restrictions	4.88	0.44	0.107	0.743	2.08	0.081	4.31	0.005 *
74. I avoid contacts with the elderly	4.63	0.84	12.33	0.000 **	2.06	0.084	15.46	0.000 **
75. I don’t meet with family members	4.23	1.21	37.97	0.000 **	1.44	0.227	2.27	0.078
76. I use disinfectants for clothes and shoes	3.66	1.41	10.48	0.001 **	2.21	0.066	4.97	0.002 *
77. Disinfection of pets paws	3.30	1.78	28.79	0.000 **	4.08	0.003 *	5.29	0.001 **
78. I have no contacts with pets	2.71	1.79	2.37	0.123	5.07	0.000 **	17.63	0.000 **
79. Plan isolation household members	2.85	1.53	0.013	0.909	4.70	0.001 **	5.73	0.001 **
80. Household nutrition plan	3.36	1.46	2.70	0.057	1.80	0.126	15.64	0.000 **
81. I have groceries	4.27	1.20	1.97	0.161	8.88	0.000 **	3.88	0.000 **
82. I have enough supplies	3.63	1.48	0.012	0.915	11.00	0.000 **	3.90	0.009 *

* *p* ≤ 0.05; ** *p* ≤ 0.01.

## Supplementary Materials

The following are available online at https://www.mdpi.com/1660-4601/17/11/4124/s1, Data: Anova results with Post hoc Tests with gender, age and education variables.

## Appendix A

1. Gender: (a) Male; (b) Female
2. I’m _________ (insert number) years old.
3. Educational level:
  - Primary school;
  - High-school;
  - Junior college;
  - Undergraduate/University;
  - Masters/doctoral studies.
4. Average monthly income for household per month:
  - Up to $275;
  - $275 to $465;
  - $466 to $740;
  - Over $741.
5. Marital status:
  - Single;
  - In a relationship;
  - Married;
  - Divorced;
  - Widow.
6. Do you have any invalidity or chronical illness? (a) Yes; (b) No.

### Appendix A.1. Knowledge about Coronavirus Caused Emergency

Please note if you “agree” (Yes) or “disagree” (No) with following statements.

**No.**	**Statements**	**Yes**	**No**
1.	I know what epidemic is.		
2.	Coronavirus is zoonotic, which means it was transferred from animals to humans.		
3.	The first COVID-19 outbreak in human population was in China at the end of 2019.		
4.	The disease novel coronavirus causes is contagious.		
5.	Symptoms manifest exclusively after 14 days.		
6.	Symptoms could manifest in the period between 0 and 28 day after the exposure to virus.		
7.	Common symptoms are fever, fatigue and dry cough.		
8.	Other symptoms include difficulty breathing, pain, diarrhea, nausea and runny nose.		

### Appendix A.2. Preparedness for Coronavirus Caused Emergency

Please indicate the degree of agreement with the statements made, where 1 stands for “I strongly disagree” and 5 for “I strongly agree”.

**No.**	**Statements**	**1**	**2**	**3**	**4**	**5**
9.	I find myself individually prepared to respond to emergencies caused by novel coronavirus epidemic.	**1**	**2**	**3**	**4**	**5**
10.	I find that my household is prepared for emergency response caused by novel coronavirus epidemic.	**1**	**2**	**3**	**4**	**5**
11.	I consider my local government prepared to respond to emergencies caused by novel coronavirus epidemic.	**1**	**2**	**3**	**4**	**5**
12.	I consider that my country is prepared to respond to emergencies caused by novel coronavirus epidemic.	**1**	**2**	**3**	**4**	**5**
13.	I find that I have sufficient knowledge to respond properly to emergencies caused by epidemics.	**1**	**2**	**3**	**4**	**5**
14.	I find that I am trained enough to respond properly to emergencies caused by epidemics.	**1**	**2**	**3**	**4**	**5**
15.	I find that I have enough supplies of food, medicine, etc.	**1**	**2**	**3**	**4**	**5**
16.	I find that I have the necessary protective equipment.	**1**	**2**	**3**	**4**	**5**
17.	I consider that I have prepared adequate response plans during the outbreak.	**1**	**2**	**3**	**4**	**5**
18.	I think we have enough knowledge within the household to respond during an epidemic.	**1**	**2**	**3**	**4**	**5**
19.	I think we have enough training within the household to respond during an epidemic.	**1**	**2**	**3**	**4**	**5**
20.	I think we have enough supplies within the household to respond during an epidemic.	**1**	**2**	**3**	**4**	**5**
21.	I think we have effective plans within the household to respond during an epidemic.	**1**	**2**	**3**	**4**	**5**
22.	I believe that my local community has enough knowledge to respond during an epidemic.	**1**	**2**	**3**	**4**	**5**
23.	I believe that my local community has enough training to respond during an epidemic.	**1**	**2**	**3**	**4**	**5**
24.	I believe that my local community has enough supplies to respond during an epidemic.	**1**	**2**	**3**	**4**	**5**
25.	I believe that my local community has effective plans to respond during an epidemic.	**1**	**2**	**3**	**4**	**5**
26.	I believe that my state has enough knowledge within the household to respond during an epidemic.	**1**	**2**	**3**	**4**	**5**
27.	I believe that my state has enough training within the household to respond during an epidemic.	**1**	**2**	**3**	**4**	**5**
28.	I believe that my state has enough supplies within the household to respond during an epidemic.	**1**	**2**	**3**	**4**	**5**
29.	I believe that my state has effective plans within the household to respond during an epidemic.	**1**	**2**	**3**	**4**	**5**
30.	I believe that first responders (police, fire and rescue units, ambulance) are ready to respond to emergencies caused by epidemics.	**1**	**2**	**3**	**4**	**5**
31.	I believe that health care institutions have enough knowledge to respond to epidemic of novel coronavirus.	**1**	**2**	**3**	**4**	**5**
32.	I believe that health care institutions have enough supplies to respond to epidemic of novel coronavirus.	**1**	**2**	**3**	**4**	**5**

### Appendix A.3. Risk Perception and Information Sources

What are the sources of information about novel coronavirus epidemic and pandemic?

Please indicate the degree of agreement with the statements made, where 1 stands for “I strongly disagree” and 5 for “I strongly agree”.

**No.**	**Statements**	**1**	**2**	**3**	**4**	**5**
33.	Television	**1**	**2**	**3**	**4**	**5**
34.	Radio	**1**	**2**	**3**	**4**	**5**
35.	Newspapers	**1**	**2**	**3**	**4**	**5**
36.	Internet	**1**	**2**	**3**	**4**	**5**
37.	Websites of international medical organizations	**1**	**2**	**3**	**4**	**5**
38.	Website of the Institute for Public Health „Dr Milan Jovanović Batut“ *	**1**	**2**	**3**	**4**	**5**
39.	Scientific journals	**1**	**2**	**3**	**4**	**5**
40.	Website of local medical institutions	**1**	**2**	**3**	**4**	**5**
41.	Addressing of statesmen and, in general, governing structures	**1**	**2**	**3**	**4**	**5**
42.	Addressing of experts (epidemiologists, medical doctors of other specialties, experts in civil protection, etc.)	**1**	**2**	**3**	**4**	**5**
43.	Social networks	**1**	**2**	**3**	**4**	**5**
44.	Family members	**1**	**2**	**3**	**4**	**5**
45.	Friends	**1**	**2**	**3**	**4**	**5**
46.	Local community	**1**	**2**	**3**	**4**	**5**
47.	Selected doctors	**1**	**2**	**3**	**4**	**5**
48.	First responders (police, fire and rescue, army units etc.)	**1**	**2**	**3**	**4**	**5**
49.	Non-governmental organizations	**1**	**2**	**3**	**4**	**5**
50.	Educational institutions	**1**	**2**	**3**	**4**	**5**

Please indicate the degree of agreement with the statements made, where 1 stands for “I strongly disagree” and 5 for “I strongly agree”.

**No.**	**Statements**	**1**	**2**	**3**	**4**	**5**
51.	The likelihood that I will be infected with the virus is low.	**1**	**2**	**3**	**4**	**5**
52.	Most people who become infected will develop mild to moderate respiratory symptoms, from which they will recover without the need for special treatment.	**1**	**2**	**3**	**4**	**5**
53.	The elderly and those with chronic illnesses (e.g., respiratory, cardiovascular, diabetes, immunity, etc.) are at the highest risk of developing severely treatable illnesses and putting them at great risk of death.	**1**	**2**	**3**	**4**	**5**
54.	I believe that even if I do get infected I will not develop serious health problems.	**1**	**2**	**3**	**4**	**5**
55.	Although they are not at risk of developing severe illnesses, I find it good that children do not go to kindergartens and schools.	**1**	**2**	**3**	**4**	**5**
56.	I think that I am in a higher risk of being infected with the virus because of the place where I live.	**1**	**2**	**3**	**4**	**5**
57.	I believe that because of the epidemic and the declared state of emergency, I am at risk of losing my job and source of income.	**1**	**2**	**3**	**4**	**5**
58.	I believe that through my behavior (taking preventative measures) I can prevent the virus from spreading.	**1**	**2**	**3**	**4**	**5**
59.	I feel that I have a responsibility to do what I can to protect people at risk.	**1**	**2**	**3**	**4**	**5**
60.	We will not be able to stop the spread of the virus by following the introduced measures.	**1**	**2**	**3**	**4**	**5**
61.	I think I have all the information I need to critically review the situation in our country caused by epidemic and decide what to do.	**1**	**2**	**3**	**4**	**5**
62.	I fear that the economic consequences of the epidemic will be great for society.	**1**	**2**	**3**	**4**	**5**
63.	I’m afraid the restriction on movement will prevent me from fulfilling my needs (take medication, make money withdrawal, buy groceries).	**1**	**2**	**3**	**4**	**5**

### Appendix A.4. Preventive Measures for Coronavirus Disease

Please indicate the degree of agreement with the statements made, where 1 stands for “I strongly disagree” and 5 for “I strongly agree”.

**No.**	**Statements**	**1**	**2**	**3**	**4**	**5**
64.	I’m washing my hands with soap and water for 20 s.	**1**	**2**	**3**	**4**	**5**
65.	I’m using disinfectants to maintain the hygiene of the room.	**1**	**2**	**3**	**4**	**5**
66.	I’m wearing protective mask.	**1**	**2**	**3**	**4**	**5**
67.	I’m wearing protective gloves.	**1**	**2**	**3**	**4**	**5**
68.	I do not touch my face (eyes, nose, mouth).	**1**	**2**	**3**	**4**	**5**
69.	I do not shake hands with acquaintances.	**1**	**2**	**3**	**4**	**5**
70.	I do not hug with family members, friends, acquaintances.	**1**	**2**	**3**	**4**	**5**
71.	I do not kiss in cheek with family members, friends, acquaintances.	**1**	**2**	**3**	**4**	**5**
72.	I maintain a recommended distance of 2 m in relation to the people.	**1**	**2**	**3**	**4**	**5**
73.	I respect the restriction of movement in public places.	**1**	**2**	**3**	**4**	**5**
74.	I avoid contacts with people over 65.	**1**	**2**	**3**	**4**	**5**
75.	I do not meet with family members with who do not live in the same household, friends, acquaintances.	**1**	**2**	**3**	**4**	**5**
76.	I use a disinfectant to clean my shoes and clothing in which I went to make my purchases, to the workplace.	**1**	**2**	**3**	**4**	**5**
77.	I am disinfecting the paws of pet upon returning from the walk.	**1**	**2**	**3**	**4**	**5**
78.	I’ve moved pets out of the living place and no longer have contact with them.	**1**	**2**	**3**	**4**	**5**
79.	I’ve made a plan with the members of my household about the modes of isolation if needed.	**1**	**2**	**3**	**4**	**5**
80.	I’ve made a plan for the diet and necessary food with household members.	**1**	**2**	**3**	**4**	**5**
81.	I have enough supplies of groceries needed for 3 days of isolation.	**1**	**2**	**3**	**4**	**5**
82.	I have enough supplies of groceries needed for 3–7 days of isolation.	**1**	**2**	**3**	**4**	**5**
83.	I have enough supplies of groceries for a month and longer.	**1**	**2**	**3**	**4**	**5**

84. Have you been engaged in any form of assistance?

1. Before epidemic: (a) Yes; (b) No.
2. During epidemic: (a) Yes; (b) No.
