# Supplementary material for: Preparedness and Preventive Behaviors for a Pandemic Disaster Caused by COVID-19 in Serbia
Source: Int J Environ Res Public Health. 2020 Jun 9;17(11):4124. doi: 10.3390/ijerph17114124 (PMC7313005; doi:10.3390/ijerph17114124)
Supplement: Supplementary file 1 [file ijerph-17-04124-s001.pdf]

Supplementary Material

Data: Anova results with Post hoc Tests with gender, age and education variable

ANOVA						
		Sum of Squares	df	Mean Square	F	Sig.
Individual preparedness	Between Groups	20.969	4	5.242	6.642	.000
	Within Groups	765.621	970	.789		
	Total	786.591	974			
Household preparedness	Between Groups	11.988	4	2.997	3.444	.008
	Within Groups	844.221	970	.870		
	Total	856.209	974			
Local government preparedness	Between Groups	21.409	4	5.352	5.110	.000
	Within Groups	1015.982	970	1.047		
	Total	1037.391	974			
State preparedness	Between Groups	19.861	4	4.965	4.607	.001
	Within Groups	1045.370	970	1.078		
	Total	1065.231	974			
Individual knowledge for responding	Between Groups	27.719	4	6.930	7.643	.000
	Within Groups	879.444	970	.907		
	Total	907.163	974			
Enough training for responding	Between Groups	31.597	4	7.899	6.988	.000
	Within Groups	1096.440	970	1.130		
	Total	1128.037	974			
Food supplies	Between Groups	44.808	4	11.202	8.955	.000
	Within Groups	1213.401	970	1.251		
	Total	1258.209	974			

Protective equipment	Between Groups	22.648	4	5.662	3.693	.005
	Within Groups	1487.044	970	1.533		
	Total	1509.692	974			
Response plans	Between Groups	48.250	4	12.063	10.952	.000
	Within Groups	1068.359	970	1.101		
	Total	1116.609	974			
Household knowledge	Between Groups	29.639	4	7.410	8.224	.000
	Within Groups	873.998	970	.901		
	Total	903.637	974			
First responders preparedness	Between Groups	20.184	4	5.046	4.878	.001
	Within Groups	1003.380	970	1.034		
	Total	1023.563	974			

Multiple Comparisons							
Tukey HSD							
Dependent Variable	(I) Education	(J) Education	Mean Difference (I-J)	Std. Error	Sig.	95% Confidence Interval	
						Lower Bound	Upper Bound
Individual preparedness	Primary sch.	Srednja	1.513*	.516	.029	.10	2.92
		Visa	1.524*	.525	.031	.09	2.96
		Fakultet	1.478*	.514	.034	.07	2.88
		Master/doktorske	1.108	.520	.207	-.31	2.53
	High-school	Primary sch.	-1.513*	.516	.029	-2.92	-.10
		Visa	.011	.126	1.000	-.33	.36
		Fakultet	-.034	.069	.988	-.22	.15
		Master/doktorske	-.404*	.102	.001	-.68	-.13
	Junior college	Primary sch.	-1.524*	.525	.031	-2.96	-.09
		High-school	-.011	.126	1.000	-.36	.33
		Fakultet	-.045	.118	.995	-.37	.28
		Master/doktorske	-.416*	.140	.026	-.80	-.03
	Undergraduate	Primary sch.	-1.478*	.514	.034	-2.88	-.07
		High-school	.034	.069	.988	-.15	.22

		Junior college	.045	.118	.995	-.28	.37
		Master/doktorske	-.370*	.092	.001	-.62	-.12
	Master/doctorate	Primary sch.	-1.108	.520	.207	-2.53	.31
		High-school	.404*	.102	.001	.13	.68
		Junior college	.416*	.140	.026	.03	.80
		Undergraduate	.370*	.092	.001	.12	.62
Household preparedness	Primary sch.	High-school	1.438	.542	.062	-.04	2.92
		Junior college	1.429	.551	.073	-.08	2.94
		Undergraduate	1.468	.540	.052	-.01	2.94
		Master/doctorate	1.216	.546	.170	-.28	2.71
	High-school	Primary sch.	-1.438	.542	.062	-2.92	.04
		Junior college	-.009	.132	1.000	-.37	.35
		Undergraduate	.030	.072	.993	-.17	.23
		Master/doctorate	-.221	.107	.236	-.51	.07
	Junior college	Primary sch.	-1.429	.551	.073	-2.94	.08
		High-school	.009	.132	1.000	-.35	.37
		Undergraduate	.039	.124	.998	-.30	.38
		Master/doctorate	-.212	.147	.600	-.61	.19
	Undergraduate	Primary sch.	-1.468	.540	.052	-2.94	.01
		High-school	-.030	.072	.993	-.23	.17
		Junior college	-.039	.124	.998	-.38	.30
		Master/doctorate	-.252	.097	.072	-.52	.01
	Master/doctorate	Primary sch.	-1.216	.546	.170	-2.71	.28
		High-school	.221	.107	.236	-.07	.51
		Junior college	.212	.147	.600	-.19	.61
		Undergraduate	.252	.097	.072	-.01	.52
Local government preparedness	Primary sch.	High-school	2.138*	.595	.003	.51	3.76
		Junior college	2.190*	.605	.003	.54	3.84
		Undergraduate	2.274*	.592	.001	.66	3.89
		Master/doctorate	2.405*	.599	.001	.77	4.04
	High-school	Primary sch.	-2.138*	.595	.003	-3.76	-.51
		Junior college	.053	.145	.996	-.34	.45
		Undergraduate	.137	.079	.416	-.08	.35
		Master/doctorate	.268	.117	.152	-.05	.59
	Junior college	Primary sch.	-2.190*	.605	.003	-3.84	-.54
		High-school	-.053	.145	.996	-.45	.34
		Undergraduate	.084	.136	.973	-.29	.46
		Master/doctorate	.215	.161	.672	-.23	.66
	Undergraduate	Primary sch.	-2.274*	.592	.001	-3.89	-.66
		High-school	-.137	.079	.416	-.35	.08
		Junior college	-.084	.136	.973	-.46	.29
		Master/doctorate	.131	.106	.732	-.16	.42

	Master/doctorate	Primary sch.	-2.405*	.599	.001	-4.04	-.77
		High-school	-.268	.117	.152	-.59	.05
		Junior college	-.215	.161	.672	-.66	.23
		Undergraduate	-.131	.106	.732	-.42	.16
State preparedness	Primary sch.	High-school	1.888*	.603	.015	.24	3.54
		Junior college	1.524	.613	.095	-.15	3.20
		Undergraduate	1.903*	.601	.014	.26	3.55
		Master/doctorate	1.973*	.607	.011	.31	3.63
	High-school	Primary sch.	-1.888*	.603	.015	-3.54	-.24
		Junior college	-.364	.147	.097	-.77	.04
		Undergraduate	.016	.080	1.000	-.20	.23
		Master/doctorate	.085	.119	.953	-.24	.41
	Junior college	Primary sch.	-1.524	.613	.095	-3.20	.15
		High-school	.364	.147	.097	-.04	.77
		Undergraduate	.379*	.138	.048	.00	.76
		Master/doctorate	.449*	.164	.049	.00	.90
	Undergraduate	Primary sch.	-1.903*	.601	.014	-3.55	-.26
		High-school	-.016	.080	1.000	-.23	.20
		Junior college	-.379*	.138	.048	-.76	.00
		Master/doctorate	.070	.108	.967	-.23	.36
	Master/doctorate	Primary sch.	-1.973*	.607	.011	-3.63	-.31
		High-school	-.085	.119	.953	-.41	.24
		Junior college	-.449*	.164	.049	-.90	.00
		Undergraduate	-.070	.108	.967	-.36	.23
Individual knowledge for responding	Primary sch.	High-school	1.638*	.553	.026	.13	3.15
		Junior college	1.286	.563	.151	-.25	2.82
		Undergraduate	1.677*	.551	.020	.17	3.18
		Master/doctorate	1.297	.557	.137	-.23	2.82
	High-school	Primary sch.	-1.638*	.553	.026	-3.15	-.13
		Junior college	-.352	.135	.069	-.72	.02
		Undergraduate	.040	.074	.983	-.16	.24
		Master/doctorate	-.340*	.109	.016	-.64	-.04
	Junior college	Primary sch.	-1.286	.563	.151	-2.82	.25
		High-school	.352	.135	.069	-.02	.72
		Undergraduate	.392*	.127	.017	.05	.74
		Master/doctorate	.012	.150	1.000	-.40	.42
	Undergraduate	Primary sch.	-1.677*	.551	.020	-3.18	-.17
		High-school	-.040	.074	.983	-.24	.16
		Junior college	-.392*	.127	.017	-.74	-.05
		Master/doctorate	-.380*	.099	.001	-.65	-.11
	Master/doctorate	Primary sch.	-1.297	.557	.137	-2.82	.23
		High-school	.340*	.109	.016	.04	.64

		Junior college	-.012	.150	1.000	-.42	.40
		Undergraduate	.380*	.099	.001	.11	.65
Enough training for responding	Primary sch.	High-school	1.963*	.618	.013	.27	3.65
		Junior college	1.667	.628	.062	-.05	3.38
		Undergraduate	1.962*	.615	.013	.28	3.64
		Master/doctorate	1.541	.622	.097	-.16	3.24
	High-school	Primary sch.	-1.963*	.618	.013	-3.65	-.27
		Junior college	-.296	.151	.284	-.71	.12
		Undergraduate	.000	.082	1.000	-.22	.22
		Master/doctorate	-.422*	.122	.005	-.76	-.09
	Junior college	Primary sch.	-1.667	.628	.062	-3.38	.05
		High-school	.296	.151	.284	-.12	.71
		Undergraduate	.296	.141	.224	-.09	.68
		Master/doctorate	-.126	.168	.944	-.58	.33
	Undergraduate	Primary sch.	-1.962*	.615	.013	-3.64	-.28
		High-school	.000	.082	1.000	-.22	.22
		Junior college	-.296	.141	.224	-.68	.09
		Master/doctorate	-.422*	.110	.001	-.72	-.12
	Master/doctorate	Primary sch.	-1.541	.622	.097	-3.24	.16
		High-school	.422*	.122	.005	.09	.76
		Junior college	.126	.168	.944	-.33	.58
		Undergraduate	.422*	.110	.001	.12	.72
Food supplies	Primary sch.	High-school	1.875*	.650	.033	.10	3.65
		Junior college	1.857*	.661	.040	.05	3.66
		Undergraduate	1.452	.647	.165	-.32	3.22
		Master/doctorate	1.432	.654	.185	-.36	3.22
	High-school	Primary sch.	-1.875*	.650	.033	-3.65	-.10
		Junior college	-.018	.158	1.000	-.45	.41
		Undergraduate	-.423*	.086	.000	-.66	-.19
		Master/doctorate	-.443*	.128	.005	-.79	-.09
	Junior college	Primary sch.	-1.857*	.661	.040	-3.66	-.05
		High-school	.018	.158	1.000	-.41	.45
		Undergraduate	-.406	.149	.051	-.81	.00
		Master/doctorate	-.425	.176	.114	-.91	.06
	Undergraduate	Primary sch.	-1.452	.647	.165	-3.22	.32
		High-school	.423*	.086	.000	.19	.66
		Junior college	.406	.149	.051	.00	.81
		Master/doctorate	-.019	.116	1.000	-.34	.30
	Master/doctorate	Primary sch.	-1.432	.654	.185	-3.22	.36
		High-school	.443*	.128	.005	.09	.79
		Junior college	.425	.176	.114	-.06	.91
		Undergraduate	.019	.116	1.000	-.30	.34

Protective equipment	Primary sch.	High-school	2.100[*]	.719	.029	.13	4.07
		Junior college	1.857	.732	.083	-.14	3.86
		Undergraduate	1.866	.717	.071	-.09	3.82
		Master/doctorate	1.784	.724	.100	-.20	3.76
	High-school	Primary sch.	-2.100[*]	.719	.029	-4.07	-.13
		Junior college	-.243	.175	.637	-.72	.24
		Undergraduate	-.234	.096	.103	-.50	.03
		Master/doctorate	-.316	.142	.171	-.70	.07
	Junior college	Primary sch.	-1.857	.732	.083	-3.86	.14
		High-school	.243	.175	.637	-.24	.72
		Undergraduate	.008	.165	1.000	-.44	.46
		Master/doctorate	-.073	.195	.996	-.61	.46
	Undergraduate	Primary sch.	-1.866	.717	.071	-3.82	.09
		High-school	.234	.096	.103	-.03	.50
		Junior college	-.008	.165	1.000	-.46	.44
		Master/doctorate	-.082	.129	.969	-.43	.27
	Master/doctorate	Primary sch.	-1.784	.724	.100	-3.76	.20
		High-school	.316	.142	.171	-.07	.70
		Junior college	.073	.195	.996	-.46	.61
		Undergraduate	.082	.129	.969	-.27	.43
Response plans	Primary sch.	High-school	1.862[*]	.610	.020	.20	3.53
		Junior college	1.714[*]	.620	.046	.02	3.41
		Undergraduate	1.978[*]	.608	.010	.32	3.64
		Master/doctorate	1.351	.614	.180	-.33	3.03
	High-school	Primary sch.	-1.862[*]	.610	.020	-3.53	-.20
		Junior college	-.148	.149	.856	-.55	.26
		Undergraduate	.116	.081	.607	-.11	.34
		Master/doctorate	-.511[*]	.120	.000	-.84	-.18
	Junior college	Primary sch.	-1.714[*]	.620	.046	-3.41	-.02
		High-school	.148	.149	.856	-.26	.55
		Undergraduate	.264	.139	.321	-.12	.65
		Master/doctorate	-.363	.166	.183	-.82	.09
	Undergraduate	Primary sch.	-1.978[*]	.608	.010	-3.64	-.32
		High-school	-.116	.081	.607	-.34	.11
		Junior college	-.264	.139	.321	-.65	.12
		Master/doctorate	-.627[*]	.109	.000	-.93	-.33
	Master/doctorate	Primary sch.	-1.351	.614	.180	-3.03	.33
		High-school	.511[*]	.120	.000	.18	.84
		Junior college	.363	.166	.183	-.09	.82
		Undergraduate	.627[*]	.109	.000	.33	.93
Household knowledge	Primary sch.	High-school	1.575[*]	.551	.035	.07	3.08
		Junior college	1.238	.561	.178	-.29	2.77

		Undergraduate	1.591*	.550	.032	.09	3.09
		Master/doctorate	1.162	.555	.224	-.36	2.68
	High-school	Primary sch.	-1.575*	.551	.035	-3.08	-.07
		Junior college	-.337	.134	.090	-.70	.03
		Undergraduate	.016	.073	.999	-.18	.22
		Master/doctorate	-.413*	.109	.002	-.71	-.12
	Junior college	Primary sch.	-1.238	.561	.178	-2.77	.29
		High-school	.337	.134	.090	-.03	.70
		Undergraduate	.353*	.126	.041	.01	.70
		Master/doctorate	-.076	.150	.987	-.49	.33
	Undergraduate	Primary sch.	-1.591*	.550	.032	-3.09	-.09
		High-school	-.016	.073	.999	-.22	.18
		Junior college	-.353*	.126	.041	-.70	-.01
		Master/doctorate	-.429*	.099	.000	-.70	-.16
	Master/doctorate	Primary sch.	-1.162	.555	.224	-2.68	.36
		High-school	.413*	.109	.002	.12	.71
		Junior college	.076	.150	.987	-.33	.49
		Undergraduate	.429*	.099	.000	.16	.70
First responders preparedness	Primary sch.	High-school	1.100	.591	.339	-.51	2.71
		Junior college	1.286	.601	.204	-.36	2.93
		Undergraduate	1.183	.589	.262	-.43	2.79
		Master/doctorate	1.541	.595	.073	-.09	3.17
	High-school	Primary sch.	-1.100	.591	.339	-2.71	.51
		Junior college	.186	.144	.698	-.21	.58
		Undergraduate	.083	.079	.830	-.13	.30
		Master/doctorate	.441*	.117	.002	.12	.76
	Junior college	Primary sch.	-1.286	.601	.204	-2.93	.36
		High-school	-.186	.144	.698	-.58	.21
		Undergraduate	-.103	.135	.942	-.47	.27
		Master/doctorate	.255	.160	.505	-.18	.69
	Undergraduate	Primary sch.	-1.183	.589	.262	-2.79	.43
		High-school	-.083	.079	.830	-.30	.13
		Junior college	.103	.135	.942	-.27	.47
		Master/doctorate	.358*	.106	.007	.07	.65
	Master/doctorate	Primary sch.	-1.541	.595	.073	-3.17	.09
		High-school	-.441*	.117	.002	-.76	-.12
		Junior college	-.255	.160	.505	-.69	.18
		Undergraduate	-.358*	.106	.007	-.65	-.07
*. The mean difference is significant at the 0.05 level.							

3.3. Risk perception and informing of coronavirus disease and education - Anova results with Post hoc

ANOVA						
		Sum of Squares	df	Mean Square	F	Sig.
Television	Between Groups	20.618	4	5.154	3.765	.005
	Within Groups	1328.053	970	1.369		
	Total	1348.671	974			
Radio	Between Groups	33.434	4	8.358	7.312	.000
	Within Groups	1108.874	970	1.143		
	Total	1142.308	974			
Newspaper	Between Groups	45.032	4	11.258	6.693	.000
	Within Groups	1631.516	970	1.682		
	Total	1676.548	974			
Internet	Between Groups	5.214	4	1.303	1.345	.251
	Within Groups	940.239	970	.969		
	Total	945.452	974			
Scientific journal	Between Groups	34.637	4	8.659	4.180	.002
	Within Groups	2009.646	970	2.072		
	Total	2044.283	974			
Local medical website	Between Groups	55.181	4	13.795	7.553	.000
	Within Groups	1771.736	970	1.827		
	Total	1826.917	974			
Addressing of a statesman	Between Groups	30.063	4	7.516	5.044	.001
	Within Groups	1445.420	970	1.490		
	Total	1475.483	974			

Addressing of an expert	Between Groups	8.114	4	2.028	2.864	.022
	Within Groups	687.019	970	.708		
	Total	695.132	974			
The social network	Between Groups	12.333	4	3.083	1.554	.185
	Within Groups	1924.614	970	1.984		
	Total	1936.948	974			
Family members	Between Groups	44.882	4	11.220	7.468	.000
	Within Groups	1457.481	970	1.503		
	Total	1502.363	974			
Friends	Between Groups	33.904	4	8.476	5.720	.000
	Within Groups	1437.426	970	1.482		
	Total	1471.329	974			
Local community	Between Groups	27.962	4	6.991	4.658	.001
	Within Groups	1455.847	970	1.501		
	Total	1483.809	974			
Chosen physician	Between Groups	54.340	4	13.585	8.275	.000
	Within Groups	1592.429	970	1.642		
	Total	1646.769	974			
First responders	Between Groups	47.992	4	11.998	6.375	.000
	Within Groups	1825.540	970	1.882		
	Total	1873.532	974			
Non-government org.	Between Groups	42.009	4	10.502	8.965	.000
	Within Groups	1136.299	970	1.171		
	Total	1178.308	974			
Educational institutions	Between Groups	105.789	4	26.447	14.335	.000

	Within Groups	1789.620	970	1.845		
	Total	1895.409	974			

Multiple Comparisons							
Tukey HSD							
Dependent Variable	(I) Education	(J) Education	Mean Difference (I-J)	Std. Error	Sig.	95% Confidence Interval	
						Lower Bound	Upper Bound
Television	Primary sch.	High-school	.950	.680	.629	-.91	2.81
		Junior college	1.238	.691	.380	-.65	3.13
		Undergraduate	1.124	.677	.460	-.73	2.97
		Master/doctorate	1.405	.685	.242	-.47	3.28
	High-school	Primary sch.	-.950	.680	.629	-2.81	.91
		Junior college	.288	.166	.410	-.16	.74
		Undergraduate	.174	.090	.306	-.07	.42
		Master/doctorate	.455*	.134	.006	.09	.82
	Junior college	Primary sch.	-1.238	.691	.380	-3.13	.65
		High-school	-.288	.166	.410	-.74	.16
		Undergraduate	-.114	.156	.948	-.54	.31
		Master/doctorate	.167	.185	.894	-.34	.67
	Undergraduate	Primary sch.	-1.124	.677	.460	-2.97	.73
		High-school	-.174	.090	.306	-.42	.07
		Junior college	.114	.156	.948	-.31	.54
		Master/doctorate	.282	.122	.140	-.05	.61
	Master/doctorate	Primary sch.	-1.405	.685	.242	-3.28	.47
		High-school	-.455*	.134	.006	-.82	-.09
		Junior college	-.167	.185	.894	-.67	.34
		Undergraduate	-.282	.122	.140	-.61	.05
Radio	Primary sch.	High-school	3.213*	.621	.000	1.51	4.91
		Junior college	3.333*	.632	.000	1.61	5.06
		Undergraduate	3.280*	.619	.000	1.59	4.97
		Master/doctorate	3.324*	.626	.000	1.61	5.03
	High-school	Primary sch.	-3.213*	.621	.000	-4.91	-1.51
		Junior college	.121	.151	.931	-.29	.53
		Undergraduate	.067	.083	.927	-.16	.29
		Master/doctorate	.112	.123	.893	-.22	.45
	Junior college	Primary sch.	-3.333*	.632	.000	-5.06	-1.61
		High-school	-.121	.151	.931	-.53	.29
		Undergraduate	-.054	.142	.996	-.44	.33

		Master/doctorate	-.009	.169	1.000	-.47	.45
	Undergraduate	Primary sch.	-3.280*	.619	.000	-4.97	-1.59
		High-school	-.067	.083	.927	-.29	.16
		Junior college	.054	.142	.996	-.33	.44
		Master/doctorate	.045	.111	.994	-.26	.35
	Master/doctorate	Primary sch.	-3.324*	.626	.000	-5.03	-1.61
		High-school	-.112	.123	.893	-.45	.22
		Junior college	.009	.169	1.000	-.45	.47
		Undergraduate	-.045	.111	.994	-.35	.26
Newspaper	Primary sch.	High-school	2.800*	.753	.002	.74	4.86
		Junior college	2.952*	.766	.001	.86	5.05
		Undergraduate	3.102*	.751	.000	1.05	5.15
		Master/doctorate	2.838*	.759	.002	.76	4.91
	High-school	Primary sch.	-2.800*	.753	.002	-4.86	-.74
		Junior college	.152	.184	.921	-.35	.65
		Undergraduate	.302*	.100	.022	.03	.58
		Master/doctorate	.038	.149	.999	-.37	.44
	Junior college	Primary sch.	-2.952*	.766	.001	-5.05	-.86
		High-school	-.152	.184	.921	-.65	.35
		Undergraduate	.150	.172	.908	-.32	.62
		Master/doctorate	-.115	.205	.981	-.67	.44
	Undergraduate	Primary sch.	-3.102*	.751	.000	-5.15	-1.05
		High-school	-.302*	.100	.022	-.58	-.03
		Junior college	-.150	.172	.908	-.62	.32
		Master/doctorate	-.264	.135	.286	-.63	.10
	Master/doctorate	Primary sch.	-2.838*	.759	.002	-4.91	-.76
		High-school	-.038	.149	.999	-.44	.37
		Junior college	.115	.205	.981	-.44	.67
		Undergraduate	.264	.135	.286	-.10	.63
Internet	Primary sch.	High-school	.875	.572	.543	-.69	2.44
		Junior college	.762	.582	.685	-.83	2.35
		Undergraduate	.747	.570	.684	-.81	2.30
		Master/doctorate	.703	.576	.740	-.87	2.28
	High-school	Primary sch.	-.875	.572	.543	-2.44	.69
		Junior college	-.113	.139	.927	-.49	.27
		Undergraduate	-.128	.076	.447	-.34	.08
		Master/doctorate	-.172	.113	.547	-.48	.14
	Junior college	Primary sch.	-.762	.582	.685	-2.35	.83
		High-school	.113	.139	.927	-.27	.49
		Undergraduate	-.015	.131	1.000	-.37	.34
		Master/doctorate	-.059	.155	.996	-.48	.37
	Undergraduate	Primary sch.	-.747	.570	.684	-2.30	.81

		High-school	.128	.076	.447	-.08	.34
		Junior college	.015	.131	1.000	-.34	.37
		Master/doctorate	-.045	.102	.992	-.32	.24
	Master/doctorate	Primary sch.	-.703	.576	.740	-2.28	.87
		High-school	.172	.113	.547	-.14	.48
		Junior college	.059	.155	.996	-.37	.48
		Undergraduate	.045	.102	.992	-.24	.32
Scientific journal	Primary sch.	High-school	2.675*	.836	.012	.39	4.96
		Junior college	3.095*	.851	.003	.77	5.42
		Undergraduate	2.667*	.833	.012	.39	4.94
		Master/doctorate	2.541*	.842	.022	.24	4.84
	High-school	Primary sch.	-2.675*	.836	.012	-4.96	-.39
		Junior college	.420	.204	.237	-.14	.98
		Undergraduate	-.008	.111	1.000	-.31	.30
		Master/doctorate	-.134	.165	.926	-.59	.32
	Junior college	Primary sch.	-3.095*	.851	.003	-5.42	-.77
		High-school	-.420	.204	.237	-.98	.14
		Undergraduate	-.429	.191	.166	-.95	.09
		Master/doctorate	-.555	.227	.105	-1.18	.07
	Undergraduate	Primary sch.	-2.667*	.833	.012	-4.94	-.39
		High-school	.008	.111	1.000	-.30	.31
		Junior college	.429	.191	.166	-.09	.95
		Master/doctorate	-.126	.150	.917	-.53	.28
	Master/doctorate	Primary sch.	-2.541*	.842	.022	-4.84	-.24
		High-school	.134	.165	.926	-.32	.59
		Junior college	.555	.227	.105	-.07	1.18
		Undergraduate	.126	.150	.917	-.28	.53
Local medical website	Primary sch.	High-school	2.538*	.785	.011	.39	4.68
		Junior college	3.000*	.799	.002	.82	5.18
		Undergraduate	2.414*	.782	.018	.28	4.55
		Master/doctorate	2.892*	.791	.002	.73	5.05
	High-school	Primary sch.	-2.538*	.785	.011	-4.68	-.39
		Junior college	.462	.191	.111	-.06	.99
		Undergraduate	-.124	.104	.761	-.41	.16
		Master/doctorate	.354	.155	.151	-.07	.78
	Junior college	Primary sch.	-3.000*	.799	.002	-5.18	-.82
		High-school	-.462	.191	.111	-.99	.06
		Undergraduate	-.586*	.180	.010	-1.08	-.10
		Master/doctorate	-.108	.213	.987	-.69	.47
	Undergraduate	Primary sch.	-2.414*	.782	.018	-4.55	-.28
		High-school	.124	.104	.761	-.16	.41
		Junior college	.586*	.180	.010	.10	1.08

		Master/doctorate	.478*	.140	.006	.09	.86
	Master/doctorate	Primary sch.	-2.892*	.791	.002	-5.05	-.73
		High-school	-.354	.155	.151	-.78	.07
		Junior college	.108	.213	.987	-.47	.69
		Undergraduate	-.478*	.140	.006	-.86	-.09
Addressing of a statesman	Primary sch.	High-school	.987	.709	.633	-.95	2.93
		Junior college	1.381	.721	.310	-.59	3.35
		Undergraduate	1.086	.707	.539	-.85	3.02
		Master/doctorate	1.514	.714	.213	-.44	3.47
	High-school	Primary sch.	-.987	.709	.633	-2.93	.95
		Junior college	.393	.173	.153	-.08	.87
		Undergraduate	.099	.094	.834	-.16	.36
		Master/doctorate	.526*	.140	.002	.14	.91
	Junior college	Primary sch.	-1.381	.721	.310	-3.35	.59
		High-school	-.393	.173	.153	-.87	.08
		Undergraduate	-.295	.162	.364	-.74	.15
		Master/doctorate	.133	.193	.959	-.39	.66
	Undergraduate	Primary sch.	-1.086	.707	.539	-3.02	.85
		High-school	-.099	.094	.834	-.36	.16
		Junior college	.295	.162	.364	-.15	.74
		Master/doctorate	.427*	.127	.007	.08	.77
	Master/doctorate	Primary sch.	-1.514	.714	.213	-3.47	.44
		High-school	-.526*	.140	.002	-.91	-.14
		Junior college	-.133	.193	.959	-.66	.39
		Undergraduate	-.427*	.127	.007	-.77	-.08
Addressing of an expert	Primary sch.	High-school	.650	.489	.673	-.69	1.99
		Junior college	.857	.497	.420	-.50	2.22
		Undergraduate	.602	.487	.730	-.73	1.93
		Master/doctorate	.811	.492	.468	-.53	2.16
	High-school	Primary sch.	-.650	.489	.673	-1.99	.69
		Junior college	.207	.119	.411	-.12	.53
		Undergraduate	-.048	.065	.948	-.23	.13
		Master/doctorate	.161	.097	.456	-.10	.42
	Junior college	Primary sch.	-.857	.497	.420	-2.22	.50
		High-school	-.207	.119	.411	-.53	.12
		Undergraduate	-.255	.112	.152	-.56	.05
		Master/doctorate	-.046	.133	.997	-.41	.32
	Undergraduate	Primary sch.	-.602	.487	.730	-1.93	.73
		High-school	.048	.065	.948	-.13	.23
		Junior college	.255	.112	.152	-.05	.56
		Master/doctorate	.209	.087	.120	-.03	.45
	Master/doctorate	Primary sch.	-.811	.492	.468	-2.16	.53

		High-school	-.161	.097	.456	-.42	.10
		Junior college	.046	.133	.997	-.32	.41
		Undergraduate	-.209	.087	.120	-.45	.03
The social network	Primary sch.	High-school	1.850	.818	.159	-.39	4.09
		Junior college	2.000	.832	.115	-.27	4.27
		Undergraduate	1.909	.815	.133	-.32	4.14
		Master/doctorate	1.946	.824	.127	-.31	4.20
	High-school	Primary sch.	-1.850	.818	.159	-4.09	.39
		Junior college	.150	.199	.944	-.39	.69
		Undergraduate	.059	.109	.983	-.24	.36
		Master/doctorate	.096	.162	.976	-.35	.54
	Junior college	Primary sch.	-2.000	.832	.115	-4.27	.27
		High-school	-.150	.199	.944	-.69	.39
		Undergraduate	-.091	.187	.988	-.60	.42
		Master/doctorate	-.054	.222	.999	-.66	.55
	Undergraduate	Primary sch.	-1.909	.815	.133	-4.14	.32
		High-school	-.059	.109	.983	-.36	.24
		Junior college	.091	.187	.988	-.42	.60
		Master/doctorate	.037	.146	.999	-.36	.44
	Master/doctorate	Primary sch.	-1.946	.824	.127	-4.20	.31
		High-school	-.096	.162	.976	-.54	.35
		Junior college	.054	.222	.999	-.55	.66
		Undergraduate	-.037	.146	.999	-.44	.36
Family members	Primary sch.	High-school	1.650	.712	.140	-.30	3.60
		Junior college	1.762	.724	.108	-.22	3.74
		Undergraduate	1.962*	.710	.046	.02	3.90
		Master/doctorate	1.432	.717	.268	-.53	3.39
	High-school	Primary sch.	-1.650	.712	.140	-3.60	.30
		Junior college	.112	.174	.968	-.36	.59
		Undergraduate	.312*	.095	.009	.05	.57
		Master/doctorate	-.218	.141	.533	-.60	.17
	Junior college	Primary sch.	-1.762	.724	.108	-3.74	.22
		High-school	-.112	.174	.968	-.59	.36
		Undergraduate	.200	.163	.734	-.24	.65
		Master/doctorate	-.329	.193	.432	-.86	.20
	Undergraduate	Primary sch.	-1.962*	.710	.046	-3.90	-.02
		High-school	-.312*	.095	.009	-.57	-.05
		Junior college	-.200	.163	.734	-.65	.24
		Master/doctorate	-.530*	.127	.000	-.88	-.18
	Master/doctorate	Primary sch.	-1.432	.717	.268	-3.39	.53
		High-school	.218	.141	.533	-.17	.60
		Junior college	.329	.193	.432	-.20	.86

		Undergraduate	.530*	.127	.000	.18	.88
Friends	Primary sch.	High-school	1.700	.707	.115	-.23	3.63
		Junior college	1.810	.719	.088	-.16	3.78
		Undergraduate	2.032*	.705	.033	.11	3.96
		Master/doctorate	1.730	.712	.109	-.22	3.68
	High-school	Primary sch.	-1.700	.707	.115	-3.63	.23
		Junior college	.110	.172	.969	-.36	.58
		Undergraduate	.332*	.094	.004	.08	.59
		Master/doctorate	.030	.140	1.000	-.35	.41
	Junior college	Primary sch.	-1.810	.719	.088	-3.78	.16
		High-school	-.110	.172	.969	-.58	.36
		Undergraduate	.223	.162	.643	-.22	.66
		Master/doctorate	-.080	.192	.994	-.60	.44
	Undergraduate	Primary sch.	-2.032*	.705	.033	-3.96	-.11
		High-school	-.332*	.094	.004	-.59	-.08
		Junior college	-.223	.162	.643	-.66	.22
		Master/doctorate	-.303	.127	.119	-.65	.04
	Master/doctorate	Primary sch.	-1.730	.712	.109	-3.68	.22
		High-school	-.030	.140	1.000	-.41	.35
		Junior college	.080	.192	.994	-.44	.60
		Undergraduate	.303	.127	.119	-.04	.65
Local community	Primary sch.	High-school	2.700*	.712	.001	.75	4.65
		Junior college	2.952*	.724	.000	.97	4.93
		Undergraduate	2.780*	.709	.001	.84	4.72
		Master/doctorate	2.892*	.717	.001	.93	4.85
	High-school	Primary sch.	-2.700*	.712	.001	-4.65	-.75
		Junior college	.252	.173	.592	-.22	.73
		Undergraduate	.080	.095	.918	-.18	.34
		Master/doctorate	.192	.141	.651	-.19	.58
	Junior college	Primary sch.	-2.952*	.724	.000	-4.93	-.97
		High-school	-.252	.173	.592	-.73	.22
		Undergraduate	-.173	.163	.826	-.62	.27
		Master/doctorate	-.060	.193	.998	-.59	.47
	Undergraduate	Primary sch.	-2.780*	.709	.001	-4.72	-.84
		High-school	-.080	.095	.918	-.34	.18
		Junior college	.173	.163	.826	-.27	.62
		Master/doctorate	.112	.127	.903	-.24	.46
	Master/doctorate	Primary sch.	-2.892*	.717	.001	-4.85	-.93
		High-school	-.192	.141	.651	-.58	.19
		Junior college	.060	.193	.998	-.47	.59
		Undergraduate	-.112	.127	.903	-.46	.24
Chosen physician	Primary sch.	High-school	2.763*	.744	.002	.73	4.80

		Junior college	3.286*	.757	.000	1.22	5.35
		Undergraduate	3.065*	.742	.000	1.04	5.09
		Master/doctorate	3.243*	.750	.000	1.19	5.29
	High-school	Primary sch.	-2.763*	.744	.002	-4.80	-.73
		Junior college	.523*	.181	.033	.03	1.02
		Undergraduate	.302*	.099	.020	.03	.57
		Master/doctorate	.481*	.147	.010	.08	.88
	Junior college	Primary sch.	-3.286*	.757	.000	-5.35	-1.22
		High-school	-.523*	.181	.033	-1.02	-.03
		Undergraduate	-.221	.170	.692	-.69	.24
		Master/doctorate	-.042	.202	1.000	-.59	.51
	Undergraduate	Primary sch.	-3.065*	.742	.000	-5.09	-1.04
		High-school	-.302*	.099	.020	-.57	-.03
		Junior college	.221	.170	.692	-.24	.69
		Master/doctorate	.179	.133	.665	-.19	.54
	Master/doctorate	Primary sch.	-3.243*	.750	.000	-5.29	-1.19
		High-school	-.481*	.147	.010	-.88	-.08
		Junior college	.042	.202	1.000	-.51	.59
		Undergraduate	-.179	.133	.665	-.54	.19
First responders	Primary sch.	High-school	2.425*	.797	.020	.25	4.60
		Junior college	2.857*	.811	.004	.64	5.07
		Undergraduate	2.500*	.794	.015	.33	4.67
		Master/doctorate	2.946*	.803	.002	.75	5.14
	High-school	Primary sch.	-2.425*	.797	.020	-4.60	-.25
		Junior college	.432	.194	.171	-.10	.96
		Undergraduate	.075	.106	.955	-.21	.36
		Master/doctorate	.521*	.157	.009	.09	.95
	Junior college	Primary sch.	-2.857*	.811	.004	-5.07	-.64
		High-school	-.432	.194	.171	-.96	.10
		Undergraduate	-.357	.182	.287	-.86	.14
		Master/doctorate	.089	.216	.994	-.50	.68
	Undergraduate	Primary sch.	-2.500*	.794	.015	-4.67	-.33
		High-school	-.075	.106	.955	-.36	.21
		Junior college	.357	.182	.287	-.14	.86
		Master/doctorate	.446*	.143	.016	.06	.84
	Master/doctorate	Primary sch.	-2.946*	.803	.002	-5.14	-.75
		High-school	-.521*	.157	.009	-.95	-.09
		Junior college	-.089	.216	.994	-.68	.50
		Undergraduate	-.446*	.143	.016	-.84	-.06
Non-government org.	Primary sch.	High-school	3.075*	.629	.000	1.36	4.79
		Junior college	3.286*	.640	.000	1.54	5.03
		Undergraduate	3.108*	.627	.000	1.40	4.82

		Master/doctorate	3.432*	.633	.000	1.70	5.16
	High-school	Primary sch.	-3.075*	.629	.000	-4.79	-1.36
		Junior college	.211	.153	.644	-.21	.63
		Undergraduate	.033	.084	.995	-.20	.26
		Master/doctorate	.357*	.124	.033	.02	.70
	Junior college	Primary sch.	-3.286*	.640	.000	-5.03	-1.54
		High-school	-.211	.153	.644	-.63	.21
		Undergraduate	-.178	.144	.729	-.57	.21
		Master/doctorate	.147	.171	.912	-.32	.61
	Undergraduate	Primary sch.	-3.108*	.627	.000	-4.82	-1.40
		High-school	-.033	.084	.995	-.26	.20
		Junior college	.178	.144	.729	-.21	.57
		Master/doctorate	.325*	.112	.032	.02	.63
	Master/doctorate	Primary sch.	-3.432*	.633	.000	-5.16	-1.70
		High-school	-.357*	.124	.033	-.70	-.02
		Junior college	-.147	.171	.912	-.61	.32
		Undergraduate	-.325*	.112	.032	-.63	-.02
Educational institutions	Primary sch.	High-school	2.288*	.789	.031	.13	4.44
		Junior college	3.286*	.803	.000	1.09	5.48
		Undergraduate	2.086	.786	.062	-.06	4.23
		Master/doctorate	2.514*	.795	.014	.34	4.69
	High-school	Primary sch.	-2.288*	.789	.031	-4.44	-.13
		Junior college	.998*	.192	.000	.47	1.52
		Undergraduate	-.201	.105	.306	-.49	.09
		Master/doctorate	.226	.156	.596	-.20	.65
	Junior college	Primary sch.	-3.286*	.803	.000	-5.48	-1.09
		High-school	-.998*	.192	.000	-1.52	-.47
		Undergraduate	-1.200*	.181	.000	-1.69	-.71
		Master/doctorate	-.772*	.214	.003	-1.36	-.19
	Undergraduate	Primary sch.	-2.086	.786	.062	-4.23	.06
		High-school	.201	.105	.306	-.09	.49
		Junior college	1.200*	.181	.000	.71	1.69
		Master/doctorate	.427*	.141	.021	.04	.81
	Master/doctorate	Primary sch.	-2.514*	.795	.014	-4.69	-.34
		High-school	-.226	.156	.596	-.65	.20
		Junior college	.772*	.214	.003	.19	1.36
		Undergraduate	-.427*	.141	.021	-.81	-.04

*. The mean difference is significant at the 0.05 level.

Table 6. One-way ANOVA results of psychological behaviours of citizens and gender, education level, and age the variables on the risk perception of coronavirus disease.

ANOVA						
		Sum of Squares	df	Mean Square	F	Sig.
The likelihood of infect.	Between Groups	12.697	4	3.174	2.523	.040
	Within Groups	1220.294	970	1.258		
	Total	1232.991	974			
Respiratory problems	Between Groups	26.237	4	6.559	5.597	.000
	Within Groups	1136.840	970	1.172		
	Total	1163.077	974			
Most severe symptoms	Between Groups	3.555	4	.889	1.633	.164
	Within Groups	527.916	970	.544		
	Total	531.471	974			
Serious health	Between Groups	21.300	4	5.325	5.064	.000
	Within Groups	1019.968	970	1.052		
	Total	1041.268	974			
Kindergarten or school	Between Groups	5.019	4	1.255	2.311	.056
	Within Groups	526.673	970	.543		
	Total	531.692	974			
A place of greater risk	Between Groups	42.891	4	10.723	4.857	.001
	Within Groups	2141.238	970	2.207		
	Total	2184.129	974			
Losing my job	Between Groups	24.498	4	6.124	2.782	.026
	Within Groups	2135.373	970	2.201		
	Total	2159.871	974			

Prevent behavior	Between Groups	9.960	4	2.490	4.240	.002
	Within Groups	569.677	970	.587		
	Total	579.637	974			
The responsibility	Between Groups	9.872	4	2.468	4.346	.002
	Within Groups	550.897	970	.568		
	Total	560.769	974			
Respecting measures	Between Groups	62.022	4	15.505	9.021	.000
	Within Groups	1667.326	970	1.719		
	Total	1729.348	974			
Information critically	Between Groups	11.614	4	2.904	2.712	.029
	Within Groups	1038.386	970	1.071		
	Total	1050.000	974			
I'm afraid of health	Between Groups	9.338	4	2.334	2.219	.065
	Within Groups	1020.354	970	1.052		
	Total	1029.692	974			
Econ. consequences	Between Groups	6.355	4	1.589	1.800	.127
	Within Groups	856.205	970	.883		
	Total	862.560	974			
Fear of restrictions	Between Groups	21.920	4	5.480	3.178	.013
	Within Groups	1672.850	970	1.725		
	Total	1694.769	974			

Multiple Comparisons							
Tukey HSD							
Dependent Variable	(I) Education	(J) Education	Mean Difference (I-J)	Std. Error	Sig.	95% Confidence Interval	
						Lower Bound	Upper Bound

The likelihood of infect.	Primary sch.	High-school	2.013*	.652	.018	.23	3.79
		Junior college	2.095*	.663	.014	.28	3.91
		Undergraduate	1.995*	.649	.019	.22	3.77
		Master/doctorate	1.973*	.656	.023	.18	3.77
	High-school	Primary sch.	-2.013*	.652	.018	-3.79	-.23
		Junior college	.083	.159	.985	-.35	.52
		Undergraduate	-.018	.087	1.000	-.25	.22
		Master/doctorate	-.040	.129	.998	-.39	.31
	Junior college	Primary sch.	-2.095*	.663	.014	-3.91	-.28
		High-school	-.083	.159	.985	-.52	.35
		Undergraduate	-.101	.149	.962	-.51	.31
		Master/doctorate	-.122	.177	.958	-.61	.36
	Undergraduate	Primary sch.	-1.995*	.649	.019	-3.77	-.22
		High-school	.018	.087	1.000	-.22	.25
		Junior college	.101	.149	.962	-.31	.51
		Master/doctorate	-.022	.117	1.000	-.34	.30
	Master/doctorate	Primary sch.	-1.973*	.656	.023	-3.77	-.18
		High-school	.040	.129	.998	-.31	.39
		Junior college	.122	.177	.958	-.36	.61
		Undergraduate	.022	.117	1.000	-.30	.34
Respiratory problems	Primary sch.	High-school	1.413	.629	.164	-.31	3.13
		Junior college	1.857*	.640	.031	.11	3.61
		Undergraduate	1.651	.627	.065	-.06	3.36
		Master/doctorate	1.378	.633	.190	-.35	3.11
	High-school	Primary sch.	-1.413	.629	.164	-3.13	.31
		Junior college	.445*	.153	.031	.03	.86
		Undergraduate	.238*	.084	.036	.01	.47
		Master/doctorate	-.034	.124	.999	-.37	.31
	Junior college	Primary sch.	-1.857*	.640	.031	-3.61	-.11
		High-school	-.445*	.153	.031	-.86	-.03
		Undergraduate	-.207	.144	.605	-.60	.19
		Master/doctorate	-.479*	.171	.041	-.95	-.01
	Undergraduate	Primary sch.	-1.651	.627	.065	-3.36	.06
		High-school	-.238*	.084	.036	-.47	-.01
		Junior college	.207	.144	.605	-.19	.60
		Master/doctorate	-.272	.113	.111	-.58	.04
	Master/doctorate	Primary sch.	-1.378	.633	.190	-3.11	.35
		High-school	.034	.124	.999	-.31	.37
		Junior college	.479*	.171	.041	.01	.95
		Undergraduate	.272	.113	.111	-.04	.58
Most severe symptoms	Primary sch.	High-school	.463	.429	.817	-.71	1.63
		Junior college	.476	.436	.811	-.72	1.67

		Undergraduate	.522	.427	.739	-.65	1.69
		Master/doctorate	.351	.432	.926	-.83	1.53
	High-school	Primary sch.	-.463	.429	.817	-1.63	.71
		Junior college	.014	.104	1.000	-.27	.30
		Undergraduate	.059	.057	.839	-.10	.21
		Master/doctorate	-.111	.085	.683	-.34	.12
	Junior college	Primary sch.	-.476	.436	.811	-1.67	.72
		High-school	-.014	.104	1.000	-.30	.27
		Undergraduate	.045	.098	.991	-.22	.31
		Master/doctorate	-.125	.116	.821	-.44	.19
	Undergraduate	Primary sch.	-.522	.427	.739	-1.69	.65
		High-school	-.059	.057	.839	-.21	.10
		Junior college	-.045	.098	.991	-.31	.22
		Master/doctorate	-.170	.077	.173	-.38	.04
	Master/doctorate	Primary sch.	-.351	.432	.926	-1.53	.83
		High-school	.111	.085	.683	-.12	.34
		Junior college	.125	.116	.821	-.19	.44
		Undergraduate	.170	.077	.173	-.04	.38
Serious health	Primary sch.	High-school	1.200	.596	.260	-.43	2.83
		Junior college	1.667*	.606	.048	.01	3.32
		Undergraduate	1.333	.594	.164	-.29	2.96
		Master/doctorate	1.541	.600	.077	-.10	3.18
	High-school	Primary sch.	-1.200	.596	.260	-2.83	.43
		Junior college	.467*	.145	.012	.07	.86
		Undergraduate	.133	.079	.444	-.08	.35
		Master/doctorate	.341*	.118	.032	.02	.66
	Junior college	Primary sch.	-1.667*	.606	.048	-3.32	-.01
		High-school	-.467*	.145	.012	-.86	-.07
		Undergraduate	-.333	.136	.104	-.71	.04
		Master/doctorate	-.126	.162	.936	-.57	.32
	Undergraduate	Primary sch.	-1.333	.594	.164	-2.96	.29
		High-school	-.133	.079	.444	-.35	.08
		Junior college	.333	.136	.104	-.04	.71
		Master/doctorate	.207	.107	.295	-.08	.50
	Master/doctorate	Primary sch.	-1.541	.600	.077	-3.18	.10
		High-school	-.341*	.118	.032	-.66	-.02
		Junior college	.126	.162	.936	-.32	.57
		Undergraduate	-.207	.107	.295	-.50	.08
Kindergarten or school	Primary sch.	High-school	.300	.428	.956	-.87	1.47
		Junior college	.048	.435	1.000	-1.14	1.24
		Undergraduate	.328	.427	.939	-.84	1.49
		Master/doctorate	.243	.431	.980	-.94	1.42

	High-school	Primary sch.	-.300	.428	.956	-1.47	.87
		Junior college	-.252	.104	.111	-.54	.03
		Undergraduate	.028	.057	.988	-.13	.18
		Master/doctorate	-.057	.085	.963	-.29	.17
	Junior college	Primary sch.	-.048	.435	1.000	-1.24	1.14
		High-school	.252	.104	.111	-.03	.54
		Undergraduate	.280*	.098	.035	.01	.55
		Master/doctorate	.196	.116	.445	-.12	.51
	Undergraduate	Primary sch.	-.328	.427	.939	-1.49	.84
		High-school	-.028	.057	.988	-.18	.13
		Junior college	-.280*	.098	.035	-.55	-.01
		Master/doctorate	-.085	.077	.803	-.29	.12
	Master/doctorate	Primary sch.	-.243	.431	.980	-1.42	.94
		High-school	.057	.085	.963	-.17	.29
		Junior college	-.196	.116	.445	-.51	.12
		Undergraduate	.085	.077	.803	-.12	.29
A place of greater risk	Primary sch.	High-school	2.100	.863	.107	-.26	4.46
		Junior college	2.381	.878	.053	-.02	4.78
		Undergraduate	2.161	.860	.089	-.19	4.51
		Master/doctorate	1.649	.869	.320	-.73	4.02
	High-school	Primary sch.	-2.100	.863	.107	-4.46	.26
		Junior college	.281	.210	.669	-.29	.86
		Undergraduate	.061	.115	.984	-.25	.37
		Master/doctorate	-.451	.171	.063	-.92	.01
	Junior college	Primary sch.	-2.381	.878	.053	-4.78	.02
		High-school	-.281	.210	.669	-.86	.29
		Undergraduate	-.220	.197	.800	-.76	.32
		Master/doctorate	-.732*	.234	.016	-1.37	-.09
	Undergraduate	Primary sch.	-2.161	.860	.089	-4.51	.19
		High-school	-.061	.115	.984	-.37	.25
		Junior college	.220	.197	.800	-.32	.76
		Master/doctorate	-.513*	.154	.008	-.93	-.09
	Master/doctorate	Primary sch.	-1.649	.869	.320	-4.02	.73
		High-school	.451	.171	.063	-.01	.92
		Junior college	.732*	.234	.016	.09	1.37
		Undergraduate	.513*	.154	.008	.09	.93
Losing my job	Primary sch.	High-school	2.325	.862	.055	-.03	4.68
		Junior college	2.619*	.877	.024	.22	5.02
		Undergraduate	2.258	.859	.066	-.09	4.61
		Master/doctorate	2.189	.868	.087	-.18	4.56
	High-school	Primary sch.	-2.325	.862	.055	-4.68	.03
		Junior college	.294	.210	.628	-.28	.87

		Undergraduate	-.067	.115	.977	-.38	.25
		Master/doctorate	-.136	.170	.931	-.60	.33
	Junior college	Primary sch.	-2.619*	.877	.024	-5.02	-.22
		High-school	-.294	.210	.628	-.87	.28
		Undergraduate	-.361	.197	.356	-.90	.18
		Master/doctorate	-.430	.234	.353	-1.07	.21
	Undergraduate	Primary sch.	-2.258	.859	.066	-4.61	.09
		High-school	.067	.115	.977	-.25	.38
		Junior college	.361	.197	.356	-.18	.90
		Master/doctorate	-.069	.154	.992	-.49	.35
	Master/doctorate	Primary sch.	-2.189	.868	.087	-4.56	.18
		High-school	.136	.170	.931	-.33	.60
		Junior college	.430	.234	.353	-.21	1.07
		Undergraduate	.069	.154	.992	-.35	.49
Prevent behavior	Primary sch.	High-school	.625	.445	.625	-.59	1.84
		Junior college	.238	.453	.985	-1.00	1.48
		Undergraduate	.478	.444	.818	-.73	1.69
		Master/doctorate	.405	.448	.895	-.82	1.63
	High-school	Primary sch.	-.625	.445	.625	-1.84	.59
		Junior college	-.387*	.108	.003	-.68	-.09
		Undergraduate	-.147	.059	.097	-.31	.02
		Master/doctorate	-.220	.088	.092	-.46	.02
	Junior college	Primary sch.	-.238	.453	.985	-1.48	1.00
		High-school	.387*	.108	.003	.09	.68
		Undergraduate	.240	.102	.127	-.04	.52
		Master/doctorate	.167	.121	.638	-.16	.50
	Undergraduate	Primary sch.	-.478	.444	.818	-1.69	.73
		High-school	.147	.059	.097	-.02	.31
		Junior college	-.240	.102	.127	-.52	.04
		Master/doctorate	-.073	.080	.890	-.29	.14
	Master/doctorate	Primary sch.	-.405	.448	.895	-1.63	.82
		High-school	.220	.088	.092	-.02	.46
		Junior college	-.167	.121	.638	-.50	.16
		Undergraduate	.073	.080	.890	-.14	.29
The responsibility	Primary sch.	High-school	.463	.438	.829	-.73	1.66
		Junior college	.190	.445	.993	-1.03	1.41
		Undergraduate	.462	.436	.827	-.73	1.65
		Master/doctorate	.216	.441	.988	-.99	1.42
	High-school	Primary sch.	-.463	.438	.829	-1.66	.73
		Junior college	-.272	.107	.081	-.56	.02
		Undergraduate	.000	.058	1.000	-.16	.16
		Master/doctorate	-.246*	.087	.036	-.48	-.01

	Junior college	Primary sch.	-.190	.445	.993	-1.41	1.03
		High-school	.272	.107	.081	-.02	.56
		Undergraduate	.272	.100	.053	.00	.55
		Master/doctorate	.026	.119	1.000	-.30	.35
	Undergraduate	Primary sch.	-.462	.436	.827	-1.65	.73
		High-school	.000	.058	1.000	-.16	.16
		Junior college	-.272	.100	.053	-.55	.00
		Master/doctorate	-.246*	.078	.015	-.46	-.03
	Master/doctorate	Primary sch.	-.216	.441	.988	-1.42	.99
		High-school	.246*	.087	.036	.01	.48
		Junior college	-.026	.119	1.000	-.35	.30
		Undergraduate	.246*	.078	.015	.03	.46
Respecting measures	Primary sch.	High-school	2.425*	.762	.013	.34	4.51
		Junior college	2.762*	.775	.003	.64	4.88
		Undergraduate	2.737*	.759	.003	.66	4.81
		Master/doctorate	3.135*	.767	.000	1.04	5.23
	High-school	Primary sch.	-2.425*	.762	.013	-4.51	-.34
		Junior college	.337	.186	.365	-.17	.84
		Undergraduate	.312*	.101	.018	.03	.59
		Master/doctorate	.710*	.150	.000	.30	1.12
	Junior college	Primary sch.	-2.762*	.775	.003	-4.88	-.64
		High-school	-.337	.186	.365	-.84	.17
		Undergraduate	-.025	.174	1.000	-.50	.45
		Master/doctorate	.373	.207	.371	-.19	.94
	Undergraduate	Primary sch.	-2.737*	.759	.003	-4.81	-.66
		High-school	-.312*	.101	.018	-.59	-.03
		Junior college	.025	.174	1.000	-.45	.50
		Master/doctorate	.399*	.136	.029	.03	.77
	Master/doctorate	Primary sch.	-3.135*	.767	.000	-5.23	-1.04
		High-school	-.710*	.150	.000	-1.12	-.30
		Junior college	-.373	.207	.371	-.94	.19
		Undergraduate	-.399*	.136	.029	-.77	-.03
Information critically	Primary sch.	High-school	1.375	.601	.150	-.27	3.02
		Junior college	1.381	.611	.159	-.29	3.05
		Undergraduate	1.457	.599	.108	-.18	3.09
		Master/doctorate	1.216	.605	.262	-.44	2.87
	High-school	Primary sch.	-1.375	.601	.150	-3.02	.27
		Junior college	.006	.146	1.000	-.39	.41
		Undergraduate	.082	.080	.843	-.14	.30
		Master/doctorate	-.159	.119	.668	-.48	.17
	Junior college	Primary sch.	-1.381	.611	.159	-3.05	.29
		High-school	-.006	.146	1.000	-.41	.39

		Undergraduate	.076	.138	.982	-.30	.45
		Master/doctorate	-.165	.163	.851	-.61	.28
	Undergraduate	Primary sch.	-1.457	.599	.108	-3.09	.18
		High-school	-.082	.080	.843	-.30	.14
		Junior college	-.076	.138	.982	-.45	.30
		Master/doctorate	-.241	.108	.166	-.53	.05
	Master/doctorate	Primary sch.	-1.216	.605	.262	-2.87	.44
		High-school	.159	.119	.668	-.17	.48
		Junior college	.165	.163	.851	-.28	.61
		Undergraduate	.241	.108	.166	-.05	.53
I'm afraid of health	Primary sch.	High-school	1.000	.596	.448	-.63	2.63
		Junior college	1.000	.606	.466	-.66	2.66
		Undergraduate	.823	.594	.637	-.80	2.45
		Master/doctorate	.973	.600	.484	-.67	2.61
	High-school	Primary sch.	-1.000	.596	.448	-2.63	.63
		Junior college	.000	.145	1.000	-.40	.40
		Undergraduate	-.177	.079	.166	-.39	.04
		Master/doctorate	-.027	.118	.999	-.35	.29
	Junior college	Primary sch.	-1.000	.606	.466	-2.66	.66
		High-school	.000	.145	1.000	-.40	.40
		Undergraduate	-.177	.136	.690	-.55	.20
		Master/doctorate	-.027	.162	1.000	-.47	.42
	Undergraduate	Primary sch.	-.823	.594	.637	-2.45	.80
		High-school	.177	.079	.166	-.04	.39
		Junior college	.177	.136	.690	-.20	.55
		Master/doctorate	.150	.107	.621	-.14	.44
	Master/doctorate	Primary sch.	-.973	.600	.484	-2.61	.67
		High-school	.027	.118	.999	-.29	.35
		Junior college	.027	.162	1.000	-.42	.47
		Undergraduate	-.150	.107	.621	-.44	.14
Econ. consequences	Primary sch.	High-school	.688	.546	.716	-.80	2.18
		Junior college	.952	.555	.425	-.56	2.47
		Undergraduate	.731	.544	.663	-.76	2.22
		Master/doctorate	.622	.550	.790	-.88	2.12
	High-school	Primary sch.	-.688	.546	.716	-2.18	.80
		Junior college	.265	.133	.271	-.10	.63
		Undergraduate	.044	.073	.975	-.15	.24
		Master/doctorate	-.066	.108	.973	-.36	.23
	Junior college	Primary sch.	-.952	.555	.425	-2.47	.56
		High-school	-.265	.133	.271	-.63	.10
		Undergraduate	-.221	.125	.391	-.56	.12
		Master/doctorate	-.331	.148	.169	-.74	.07

	Undergraduate	Primary sch.	-.731	.544	.663	-2.22	.76
		High-school	-.044	.073	.975	-.24	.15
		Junior college	.221	.125	.391	-.12	.56
		Master/doctorate	-.110	.098	.795	-.38	.16
	Master/doctorate	Primary sch.	-.622	.550	.790	-2.12	.88
		High-school	.066	.108	.973	-.23	.36
		Junior college	.331	.148	.169	-.07	.74
		Undergraduate	.110	.098	.795	-.16	.38
Fear of restrictions	Primary sch.	High-school	2.425*	.763	.013	.34	4.51
		Junior college	2.238*	.776	.033	.12	4.36
		Undergraduate	2.425*	.760	.013	.35	4.50
		Master/doctorate	2.243*	.768	.029	.14	4.34
	High-school	Primary sch.	-2.425*	.763	.013	-4.51	-.34
		Junior college	-.187	.186	.853	-.69	.32
		Undergraduate	.000	.101	1.000	-.28	.28
		Master/doctorate	-.182	.151	.748	-.59	.23
	Junior college	Primary sch.	-2.238*	.776	.033	-4.36	-.12
		High-school	.187	.186	.853	-.32	.69
		Undergraduate	.187	.175	.822	-.29	.66
		Master/doctorate	.005	.207	1.000	-.56	.57
	Undergraduate	Primary sch.	-2.425*	.760	.013	-4.50	-.35
		High-school	.000	.101	1.000	-.28	.28
		Junior college	-.187	.175	.822	-.66	.29
		Master/doctorate	-.181	.136	.673	-.55	.19
	Master/doctorate	Primary sch.	-2.243*	.768	.029	-4.34	-.14
		High-school	.182	.151	.748	-.23	.59
		Junior college	-.005	.207	1.000	-.57	.56
		Undergraduate	.181	.136	.673	-.19	.55
*. The mean difference is significant at the 0.05 level.							

Table 7. One-way ANOVA results of different groups of independent variable and variables on the preventive measures for coronavirus diseases.

ANOVA						
		Sum of Squares	df	Mean Square	F	Sig.
I wash my hands with soap	Between Groups	.376	4	.094	.315	.868
	Within Groups	289.433	970	.298		

	Total	289.809	974			
I'm disinfecting my hands	Between Groups	7.428	4	1.857	2.416	.047
	Within Groups	745.655	970	.769		
	Total	753.083	974			
I wear a protective mask	Between Groups	22.609	4	5.652	1.972	.097
	Within Groups	2780.314	970	2.866		
	Total	2802.923	974			
I wear protective gloves	Between Groups	19.262	4	4.815	1.724	.142
	Within Groups	2709.778	970	2.794		
	Total	2729.040	974			
I don't touch my face	Between Groups	11.817	4	2.954	2.165	.071
	Within Groups	1323.820	970	1.365		
	Total	1335.637	974			
I don't shake hands with acquaintances	Between Groups	12.748	4	3.187	4.446	.001
	Within Groups	695.289	970	.717		
	Total	708.037	974			
I'm not hugging others	Between Groups	32.735	4	8.184	6.076	.000
	Within Groups	1306.428	970	1.347		
	Total	1339.163	974			
I do not kiss others	Between Groups	46.023	4	11.506	7.838	.000
	Within Groups	1423.940	970	1.468		
	Total	1469.963	974			
Maintaining recommended distance	Between Groups	39.065	4	9.766	7.922	.000
	Within Groups	1195.772	970	1.233		
	Total	1234.837	974			

I respect movement restrictions	Between Groups	1.662	4	.416	2.083	.081
	Within Groups	193.568	970	.200		
	Total	195.231	974			
I avoid contacts with the elderly	Between Groups	5.806	4	1.451	2.061	.084
	Within Groups	683.271	970	.704		
	Total	689.077	974			
I don't meet with family members	Between Groups	8.296	4	2.074	1.414	.227
	Within Groups	1423.156	970	1.467		
	Total	1431.452	974			
I use disinfectants for clothes and shoes	Between Groups	17.535	4	4.384	2.210	.066
	Within Groups	1923.733	970	1.983		
	Total	1941.268	974			
Disinfection of pets paws	Between Groups	51.544	4	12.886	4.081	.003
	Within Groups	3062.604	970	3.157		
	Total	3114.148	974			
I have no contacts with pets	Between Groups	64.611	4	16.153	5.072	.000
	Within Groups	3089.081	970	3.185		
	Total	3153.692	974			
Plan isolation household members	Between Groups	43.888	4	10.972	4.703	.001
	Within Groups	2262.844	970	2.333		
	Total	2306.732	974			
Household nutrition plan	Between Groups	15.392	4	3.848	1.804	.126
	Within Groups	2069.248	970	2.133		
	Total	2084.640	974			
I have groceries	Between Groups	49.635	4	12.409	8.884	.000

	Within Groups	1354.882	970	1.397		
	Total	1404.517	974			
I have enough supplies	Between Groups	93.721	4	23.430	11.000	.000
	Within Groups	2066.132	970	2.130		
	Total	2159.852	974			

Multiple Comparisons							
Tukey HSD							
Dependent Variable	(I) Education	(J) Eduction	Mean Difference (I-J)	Std. Error	Sig.	95% Confidence Interval	
						Lower Bound	Upper Bound
I wash my hands with soap	Primary sch.	Srednja	.250	.317	.934	-.62	1.12
		High-school	.238	.323	.948	-.64	1.12
		Undergraduate	.215	.316	.961	-.65	1.08
		Master/doctorate	.216	.320	.961	-.66	1.09
	Srednja	Primary sch.	-.250	.317	.934	-1.12	.62
		High-school	-.012	.077	1.000	-.22	.20
		Undergraduate	-.035	.042	.922	-.15	.08
		Master/doctorate	-.034	.063	.983	-.21	.14
	High-school	Primary sch.	-.238	.323	.948	-1.12	.64
		Srednja	.012	.077	1.000	-.20	.22
		Undergraduate	-.023	.073	.998	-.22	.18
		Master/doctorate	-.022	.086	.999	-.26	.21
	Undergraduate	Primary sch.	-.215	.316	.961	-1.08	.65
		Srednja	.035	.042	.922	-.08	.15
		High-school	.023	.073	.998	-.18	.22
		Master/doctorate	.001	.057	1.000	-.15	.16
	Master/doctorate	Primary sch.	-.216	.320	.961	-1.09	.66
		Srednja	.034	.063	.983	-.14	.21
		High-school	.022	.086	.999	-.21	.26
		Undergraduate	-.001	.057	1.000	-.16	.15
I'm disinfecting my hands	Primary sch.	Srednja	.638	.509	.721	-.75	2.03
		High-school	.667	.518	.700	-.75	2.08
		Undergraduate	.489	.508	.871	-.90	1.88
		Master/doctorate	.405	.513	.933	-1.00	1.81

	Srednja	Primary sch.	-.638	.509	.721	-2.03	.75
		High-school	.029	.124	.999	-.31	.37
		Undergraduate	-.148	.068	.184	-.33	.04
		Master/doctorate	-.232	.101	.144	-.51	.04
	High-school	Primary sch.	-.667	.518	.700	-2.08	.75
		Srednja	-.029	.124	.999	-.37	.31
		Undergraduate	-.177	.117	.548	-.50	.14
		Master/doctorate	-.261	.138	.324	-.64	.12
	Undergraduate	Primary sch.	-.489	.508	.871	-1.88	.90
		Srednja	.148	.068	.184	-.04	.33
		High-school	.177	.117	.548	-.14	.50
		Master/doctorate	-.084	.091	.889	-.33	.17
	Master/doctorate	Primary sch.	-.405	.513	.933	-1.81	1.00
		Srednja	.232	.101	.144	-.04	.51
		High-school	.261	.138	.324	-.12	.64
		Undergraduate	.084	.091	.889	-.17	.33
I wear a protective mask	Primary sch.	Srednja	1.850	.984	.328	-.84	4.54
		High-school	1.810	1.000	.369	-.92	4.54
		Undergraduate	1.667	.980	.434	-1.01	4.35
		Master/doctorate	2.000	.991	.258	-.71	4.71
	Srednja	Primary sch.	-1.850	.984	.328	-4.54	.84
		High-school	-.040	.240	1.000	-.70	.61
		Undergraduate	-.183	.131	.626	-.54	.17
		Master/doctorate	.150	.194	.939	-.38	.68
	High-school	Primary sch.	-1.810	1.000	.369	-4.54	.92
		Srednja	.040	.240	1.000	-.61	.70
		Undergraduate	-.143	.225	.969	-.76	.47
		Master/doctorate	.190	.267	.953	-.54	.92
	Undergraduate	Primary sch.	-1.667	.980	.434	-4.35	1.01
		Srednja	.183	.131	.626	-.17	.54
		High-school	.143	.225	.969	-.47	.76
		Master/doctorate	.333	.176	.321	-.15	.81
	Master/doctorate	Primary sch.	-2.000	.991	.258	-4.71	.71
		Srednja	-.150	.194	.939	-.68	.38
		High-school	-.190	.267	.953	-.92	.54
		Undergraduate	-.333	.176	.321	-.81	.15
I wear protective gloves	Primary sch.	Srednja	1.763	.971	.365	-.89	4.42
		High-school	2.143	.988	.192	-.56	4.84
		Undergraduate	1.871	.968	.300	-.77	4.52
		Master/doctorate	1.730	.978	.393	-.94	4.40
	Srednja	Primary sch.	-1.763	.971	.365	-4.42	.89
		High-school	.380	.237	.493	-.27	1.03

		Undergraduate	.108	.129	.918	-.24	.46
		Master/doctorate	-.033	.192	1.000	-.56	.49
	High-school	Primary sch.	-2.143	.988	.192	-4.84	.56
		Srednja	-.380	.237	.493	-1.03	.27
		Undergraduate	-.272	.222	.737	-.88	.34
		Master/doctorate	-.413	.264	.519	-1.13	.31
	Undergraduate	Primary sch.	-1.871	.968	.300	-4.52	.77
		Srednja	-.108	.129	.918	-.46	.24
		High-school	.272	.222	.737	-.34	.88
		Master/doctorate	-.141	.174	.927	-.62	.33
	Master/doctorate	Primary sch.	-1.730	.978	.393	-4.40	.94
		Srednja	.033	.192	1.000	-.49	.56
		High-school	.413	.264	.519	-.31	1.13
		Undergraduate	.141	.174	.927	-.33	.62
I don't touch my face	Primary sch.	Srednja	.938	.679	.640	-.92	2.79
		High-school	1.333	.690	.301	-.55	3.22
		Undergraduate	1.075	.676	.504	-.77	2.92
		Master/doctorate	1.027	.684	.561	-.84	2.90
	Srednja	Primary sch.	-.938	.679	.640	-2.79	.92
		High-school	.396	.165	.118	-.06	.85
		Undergraduate	.138	.090	.545	-.11	.38
		Master/doctorate	.090	.134	.963	-.28	.46
	High-school	Primary sch.	-1.333	.690	.301	-3.22	.55
		Srednja	-.396	.165	.118	-.85	.06
		Undergraduate	-.258	.155	.458	-.68	.17
		Master/doctorate	-.306	.184	.458	-.81	.20
	Undergraduate	Primary sch.	-1.075	.676	.504	-2.92	.77
		Srednja	-.138	.090	.545	-.38	.11
		High-school	.258	.155	.458	-.17	.68
		Master/doctorate	-.048	.121	.995	-.38	.28
	Master/doctorate	Primary sch.	-1.027	.684	.561	-2.90	.84
		Srednja	-.090	.134	.963	-.46	.28
		High-school	.306	.184	.458	-.20	.81
		Undergraduate	.048	.121	.995	-.28	.38
I don't shake hands with acquaintances	Primary sch.	Srednja	.438	.492	.901	-.91	1.78
		High-school	.286	.500	.979	-1.08	1.65
		Undergraduate	.382	.490	.937	-.96	1.72
		Master/doctorate	.054	.495	1.000	-1.30	1.41
	Srednja	Primary sch.	-.438	.492	.901	-1.78	.91
		High-school	-.152	.120	.712	-.48	.18
		Undergraduate	-.056	.065	.914	-.23	.12
		Master/doctorate	-.383*	.097	.001	-.65	-.12

	High-school	Primary sch.	-.286	.500	.979	-1.65	1.08
		Srednja	.152	.120	.712	-.18	.48
		Undergraduate	.096	.113	.914	-.21	.40
		Master/doctorate	-.232	.134	.413	-.60	.13
	Undergraduate	Primary sch.	-.382	.490	.937	-1.72	.96
		Srednja	.056	.065	.914	-.12	.23
		High-school	-.096	.113	.914	-.40	.21
		Master/doctorate	-.328*	.088	.002	-.57	-.09
	Master/doctorate	Primary sch.	-.054	.495	1.000	-1.41	1.30
		Srednja	.383*	.097	.001	.12	.65
		High-school	.232	.134	.413	-.13	.60
		Undergraduate	.328*	.088	.002	.09	.57
I'm not hugging others	Primary sch.	Srednja	.925	.674	.646	-.92	2.77
		High-school	.286	.686	.994	-1.59	2.16
		Undergraduate	.731	.672	.813	-1.10	2.57
		Master/doctorate	.432	.679	.969	-1.42	2.29
	Srednja	Primary sch.	-.925	.674	.646	-2.77	.92
		High-school	-.639*	.164	.001	-1.09	-.19
		Undergraduate	-.194	.090	.195	-.44	.05
		Master/doctorate	-.493*	.133	.002	-.86	-.13
	High-school	Primary sch.	-.286	.686	.994	-2.16	1.59
		Srednja	.639*	.164	.001	.19	1.09
		Undergraduate	.445*	.154	.032	.02	.87
		Master/doctorate	.147	.183	.930	-.35	.65
	Undergraduate	Primary sch.	-.731	.672	.813	-2.57	1.10
		Srednja	.194	.090	.195	-.05	.44
		High-school	-.445*	.154	.032	-.87	-.02
		Master/doctorate	-.299	.121	.097	-.63	.03
	Master/doctorate	Primary sch.	-.432	.679	.969	-2.29	1.42
		Srednja	.493*	.133	.002	.13	.86
		High-school	-.147	.183	.930	-.65	.35
		Undergraduate	.299	.121	.097	-.03	.63
I do not kiss others	Primary sch.	Srednja	.862	.704	.737	-1.06	2.79
		High-school	.190	.716	.999	-1.77	2.15
		Undergraduate	.753	.701	.820	-1.16	2.67
		Master/doctorate	.270	.709	.996	-1.67	2.21
	Srednja	Primary sch.	-.862	.704	.737	-2.79	1.06
		High-school	-.672*	.172	.001	-1.14	-.20
		Undergraduate	-.110	.094	.766	-.37	.15
		Master/doctorate	-.592*	.139	.000	-.97	-.21
	High-school	Primary sch.	-.190	.716	.999	-2.15	1.77
		Srednja	.672*	.172	.001	.20	1.14

		Undergraduate	.562*	.161	.005	.12	1.00
		Master/doctorate	.080	.191	.994	-.44	.60
	Undergraduate	Primary sch.	-.753	.701	.820	-2.67	1.16
		Srednja	.110	.094	.766	-.15	.37
		High-school	-.562*	.161	.005	-1.00	-.12
		Master/doctorate	-.482*	.126	.001	-.83	-.14
	Master/doctorate	Primary sch.	-.270	.709	.996	-2.21	1.67
		Srednja	.592*	.139	.000	.21	.97
		High-school	-.080	.191	.994	-.60	.44
		Undergraduate	.482*	.126	.001	.14	.83
Maintaining recommended distance	Primary sch.	Srednja	1.013	.645	.517	-.75	2.78
		High-school	.524	.656	.931	-1.27	2.32
		Undergraduate	.909	.643	.619	-.85	2.67
		Master/doctorate	.405	.650	.971	-1.37	2.18
	Srednja	Primary sch.	-1.013	.645	.517	-2.78	.75
		High-school	-.489*	.157	.017	-.92	-.06
		Undergraduate	-.104	.086	.744	-.34	.13
		Master/doctorate	-.607*	.127	.000	-.96	-.26
	High-school	Primary sch.	-.524	.656	.931	-2.32	1.27
		Srednja	.489*	.157	.017	.06	.92
		Undergraduate	.385	.148	.070	-.02	.79
		Master/doctorate	-.118	.175	.962	-.60	.36
	Undergraduate	Primary sch.	-.909	.643	.619	-2.67	.85
		Srednja	.104	.086	.744	-.13	.34
		High-school	-.385	.148	.070	-.79	.02
		Master/doctorate	-.503*	.115	.000	-.82	-.19
	Master/doctorate	Primary sch.	-.405	.650	.971	-2.18	1.37
		Srednja	.607*	.127	.000	.26	.96
		High-school	.118	.175	.962	-.36	.60
		Undergraduate	.503*	.115	.000	.19	.82
I respect movement restrictions	Primary sch.	Srednja	.100	.260	.995	-.61	.81
		High-school	.048	.264	1.000	-.67	.77
		Undergraduate	.156	.259	.975	-.55	.86
		Master/doctorate	.054	.261	1.000	-.66	.77
	Srednja	Primary sch.	-.100	.260	.995	-.81	.61
		High-school	-.052	.063	.922	-.23	.12
		Undergraduate	.056	.034	.484	-.04	.15
		Master/doctorate	-.046	.051	.898	-.19	.09
	High-school	Primary sch.	-.048	.264	1.000	-.77	.67
		Srednja	.052	.063	.922	-.12	.23
		Undergraduate	.108	.059	.360	-.05	.27
		Master/doctorate	.006	.070	1.000	-.19	.20

	Undergraduate	Primary sch.	-.156	.259	.975	-.86	.55
		Srednja	-.056	.034	.484	-.15	.04
		High-school	-.108	.059	.360	-.27	.05
		Master/doctorate	-.102	.046	.183	-.23	.03
	Master/doctorate	Primary sch.	-.054	.261	1.000	-.77	.66
		Srednja	.046	.051	.898	-.09	.19
		High-school	-.006	.070	1.000	-.20	.19
		Undergraduate	.102	.046	.183	-.03	.23
I avoid contacts with the elderly	Primary sch.	Srednja	.450	.488	.888	-.88	1.78
		High-school	.143	.496	.998	-1.21	1.50
		Undergraduate	.376	.486	.938	-.95	1.70
		Master/doctorate	.297	.491	.974	-1.04	1.64
	Srednja	Primary sch.	-.450	.488	.888	-1.78	.88
		High-school	-.307	.119	.074	-.63	.02
		Undergraduate	-.074	.065	.787	-.25	.10
		Master/doctorate	-.153	.096	.507	-.42	.11
	High-school	Primary sch.	-.143	.496	.998	-1.50	1.21
		Srednja	.307	.119	.074	-.02	.63
		Undergraduate	.233	.112	.224	-.07	.54
		Master/doctorate	.154	.132	.770	-.21	.52
	Undergraduate	Primary sch.	-.376	.486	.938	-1.70	.95
		Srednja	.074	.065	.787	-.10	.25
		High-school	-.233	.112	.224	-.54	.07
		Master/doctorate	-.079	.087	.895	-.32	.16
	Master/doctorate	Primary sch.	-.297	.491	.974	-1.64	1.04
		Srednja	.153	.096	.507	-.11	.42
		High-school	-.154	.132	.770	-.52	.21
		Undergraduate	.079	.087	.895	-.16	.32
I don't meet with family members	Primary sch.	Srednja	.888	.704	.715	-1.04	2.81
		High-school	.857	.716	.753	-1.10	2.81
		Undergraduate	.747	.701	.824	-1.17	2.66
		Master/doctorate	.622	.709	.905	-1.32	2.56
	Srednja	Primary sch.	-.888	.704	.715	-2.81	1.04
		High-school	-.030	.171	1.000	-.50	.44
		Undergraduate	-.140	.094	.563	-.40	.12
		Master/doctorate	-.266	.139	.311	-.65	.11
	High-school	Primary sch.	-.857	.716	.753	-2.81	1.10
		Srednja	.030	.171	1.000	-.44	.50
		Undergraduate	-.110	.161	.960	-.55	.33
		Master/doctorate	-.236	.191	.732	-.76	.29
	Undergraduate	Primary sch.	-.747	.701	.824	-2.66	1.17
		Srednja	.140	.094	.563	-.12	.40

		High-school	.110	.161	.960	-.33	.55
		Master/doctorate	-.126	.126	.856	-.47	.22
	Master/doctorate	Primary sch.	-.622	.709	.905	-2.56	1.32
		Srednja	.266	.139	.311	-.11	.65
		High-school	.236	.191	.732	-.29	.76
		Undergraduate	.126	.126	.856	-.22	.47
I use disinfectants for clothes and shoes	Primary sch.	Srednja	1.538	.818	.329	-.70	3.77
		High-school	1.238	.832	.571	-1.04	3.51
		Undergraduate	1.296	.815	.505	-.93	3.52
		Master/doctorate	1.243	.824	.557	-1.01	3.50
	Srednja	Primary sch.	-1.538	.818	.329	-3.77	.70
		High-school	-.299	.199	.561	-.84	.25
		Undergraduate	-.242	.109	.172	-.54	.06
		Master/doctorate	-.294	.162	.362	-.74	.15
	High-school	Primary sch.	-1.238	.832	.571	-3.51	1.04
		Srednja	.299	.199	.561	-.25	.84
		Undergraduate	.058	.187	.998	-.45	.57
		Master/doctorate	.005	.222	1.000	-.60	.61
	Undergraduate	Primary sch.	-1.296	.815	.505	-3.52	.93
		Srednja	.242	.109	.172	-.06	.54
		High-school	-.058	.187	.998	-.57	.45
		Master/doctorate	-.052	.146	.996	-.45	.35
	Master/doctorate	Primary sch.	-1.243	.824	.557	-3.50	1.01
		Srednja	.294	.162	.362	-.15	.74
		High-school	-.005	.222	1.000	-.61	.60
		Undergraduate	.052	.146	.996	-.35	.45
Disinfection of pets paws	Primary sch.	Srednja	1.563	1.032	.554	-1.26	4.38
		High-school	2.476	1.050	.128	-.39	5.35
		Undergraduate	1.677	1.029	.478	-1.13	4.49
		Master/doctorate	1.730	1.040	.457	-1.11	4.57
	Srednja	Primary sch.	-1.563	1.032	.554	-4.38	1.26
		High-school	.914*	.252	.003	.23	1.60
		Undergraduate	.115	.137	.919	-.26	.49
		Master/doctorate	.167	.204	.925	-.39	.72
	High-school	Primary sch.	-2.476	1.050	.128	-5.35	.39
		Srednja	-.914*	.252	.003	-1.60	-.23
		Undergraduate	-.799*	.236	.007	-1.44	-.15
		Master/doctorate	-.746	.280	.060	-1.51	.02
	Undergraduate	Primary sch.	-1.677	1.029	.478	-4.49	1.13
		Srednja	-.115	.137	.919	-.49	.26
		High-school	.799*	.236	.007	.15	1.44
		Master/doctorate	.052	.185	.999	-.45	.56

	Master/doctorate	Primary sch.	-1.730	1.040	.457	-4.57	1.11
		Srednja	-.167	.204	.925	-.72	.39
		High-school	.746	.280	.060	-.02	1.51
		Undergraduate	-.052	.185	.999	-.56	.45
I have no contacts with pets	Primary sch.	Srednja	2.288	1.037	.178	-.55	5.12
		High-school	3.143*	1.055	.025	.26	6.02
		Undergraduate	2.231	1.033	.196	-.59	5.05
		Master/doctorate	2.189	1.044	.222	-.66	5.04
	Srednja	Primary sch.	-2.288	1.037	.178	-5.12	.55
		High-school	.855*	.253	.007	.16	1.55
		Undergraduate	-.056	.138	.994	-.43	.32
		Master/doctorate	-.098	.205	.989	-.66	.46
	High-school	Primary sch.	-3.143*	1.055	.025	-6.02	-.26
		Srednja	-.855*	.253	.007	-1.55	-.16
		Undergraduate	-.912*	.237	.001	-1.56	-.26
		Master/doctorate	-.954*	.281	.007	-1.72	-.18
	Undergraduate	Primary sch.	-2.231	1.033	.196	-5.05	.59
		Srednja	.056	.138	.994	-.32	.43
		High-school	.912*	.237	.001	.26	1.56
		Master/doctorate	-.042	.185	.999	-.55	.46
	Master/doctorate	Primary sch.	-2.189	1.044	.222	-5.04	.66
		Srednja	.098	.205	.989	-.46	.66
		High-school	.954*	.281	.007	.18	1.72
		Undergraduate	.042	.185	.999	-.46	.55
Plan isolation household members	Primary sch.	Srednja	1.925	.887	.192	-.50	4.35
		High-school	2.190	.903	.109	-.28	4.66
		Undergraduate	2.296	.884	.072	-.12	4.71
		Master/doctorate	1.919	.894	.201	-.52	4.36
	Srednja	Primary sch.	-1.925	.887	.192	-4.35	.50
		High-school	.265	.216	.735	-.33	.86
		Undergraduate	.371*	.118	.015	.05	.69
		Master/doctorate	-.006	.175	1.000	-.49	.47
	High-school	Primary sch.	-2.190	.903	.109	-4.66	.28
		Srednja	-.265	.216	.735	-.86	.33
		Undergraduate	.105	.203	.986	-.45	.66
		Master/doctorate	-.272	.241	.792	-.93	.39
	Undergraduate	Primary sch.	-2.296	.884	.072	-4.71	.12
		Srednja	-.371*	.118	.015	-.69	-.05
		High-school	-.105	.203	.986	-.66	.45
		Master/doctorate	-.377	.159	.123	-.81	.06
	Master/doctorate	Primary sch.	-1.919	.894	.201	-4.36	.52
		Srednja	.006	.175	1.000	-.47	.49

		High-school	.272	.241	.792	-.39	.93
		Undergraduate	.377	.159	.123	-.06	.81
Household nutrition plan	Primary sch.	Srednja	1.675	.849	.279	-.64	3.99
		High-school	1.714	.863	.273	-.64	4.07
		Undergraduate	1.672	.846	.278	-.64	3.98
		Master/doctorate	1.405	.855	.469	-.93	3.74
	Srednja	Primary sch.	-1.675	.849	.279	-3.99	.64
		High-school	.039	.207	1.000	-.53	.60
		Undergraduate	-.003	.113	1.000	-.31	.31
		Master/doctorate	-.270	.168	.493	-.73	.19
	High-school	Primary sch.	-1.714	.863	.273	-4.07	.64
		Srednja	-.039	.207	1.000	-.60	.53
		Undergraduate	-.042	.194	1.000	-.57	.49
		Master/doctorate	-.309	.230	.666	-.94	.32
	Undergraduate	Primary sch.	-1.672	.846	.278	-3.98	.64
		Srednja	.003	.113	1.000	-.31	.31
		High-school	.042	.194	1.000	-.49	.57
		Master/doctorate	-.267	.152	.400	-.68	.15
	Master/doctorate	Primary sch.	-1.405	.855	.469	-3.74	.93
		Srednja	.270	.168	.493	-.19	.73
		High-school	.309	.230	.666	-.32	.94
		Undergraduate	.267	.152	.400	-.15	.68
I have groceries	Primary sch.	Srednja	.975	.687	.615	-.90	2.85
		High-school	1.048	.698	.563	-.86	2.96
		Undergraduate	.688	.684	.853	-1.18	2.56
		Master/doctorate	.243	.692	.997	-1.65	2.13
	Srednja	Primary sch.	-.975	.687	.615	-2.85	.90
		High-school	.073	.167	.993	-.38	.53
		Undergraduate	-.287*	.091	.015	-.54	-.04
		Master/doctorate	-.732*	.136	.000	-1.10	-.36
	High-school	Primary sch.	-1.048	.698	.563	-2.96	.86
		Srednja	-.073	.167	.993	-.53	.38
		Undergraduate	-.359	.157	.149	-.79	.07
		Master/doctorate	-.804*	.186	.000	-1.31	-.29
	Undergraduate	Primary sch.	-.688	.684	.853	-2.56	1.18
		Srednja	.287*	.091	.015	.04	.54
		High-school	.359	.157	.149	-.07	.79
		Master/doctorate	-.445*	.123	.003	-.78	-.11
	Master/doctorate	Primary sch.	-.243	.692	.997	-2.13	1.65
		Srednja	.732*	.136	.000	.36	1.10
		High-school	.804*	.186	.000	.29	1.31
		Undergraduate	.445*	.123	.003	.11	.78

I have enough supplies	Primary sch.	Srednja	1.775	.848	.224	-.54	4.09
		High-school	1.905	.862	.177	-.45	4.26
		Undergraduate	1.231	.845	.591	-1.08	3.54
		Master/doctorate	.946	.854	.803	-1.39	3.28
	Srednja	Primary sch.	-1.775	.848	.224	-4.09	.54
		High-school	.130	.207	.971	-.43	.69
		Undergraduate	-.544*	.113	.000	-.85	-.24
		Master/doctorate	-.829*	.168	.000	-1.29	-.37
	High-school	Primary sch.	-1.905	.862	.177	-4.26	.45
		Srednja	-.130	.207	.971	-.69	.43
		Undergraduate	-.674*	.194	.005	-1.20	-.14
		Master/doctorate	-.959*	.230	.000	-1.59	-.33
	Undergraduate	Primary sch.	-1.231	.845	.591	-3.54	1.08
		Srednja	.544*	.113	.000	.24	.85
		High-school	.674*	.194	.005	.14	1.20
		Master/doctorate	-.285	.152	.329	-.70	.13
	Master/doctorate	Primary sch.	-.946	.854	.803	-3.28	1.39
		Srednja	.829*	.168	.000	.37	1.29
		High-school	.959*	.230	.000	.33	1.59
		Undergraduate	.285	.152	.329	-.13	.70
*. The mean difference is significant at the 0.05 level.							

Anova test for age

ANOVA						
		Sum of Squares	df	Mean Square	F	Sig.
Individual preparedness	Between Groups	16.368	3	5.456	6.897	.000
	Within Groups	763.354	965	.791		
	Total	779.721	968			
Household preparedness	Between Groups	5.614	3	1.871	2.140	.094
	Within Groups	843.934	965	.875		
	Total	849.548	968			
Community preparedness	Between Groups	14.830	3	4.943	4.736	.003

	Within Groups	1007.232	965	1.044		
	Total	1022.062	968			
State preparedness	Between Groups	8.530	3	2.843	2.623	.049
	Within Groups	1046.058	965	1.084		
	Total	1054.588	968			
Enough personal knowledge	Between Groups	28.815	3	9.605	10.651	.000
	Within Groups	870.219	965	.902		
	Total	899.034	968			
Enough personal training	Between Groups	35.786	3	11.929	10.667	.000
	Within Groups	1079.100	965	1.118		
	Total	1114.885	968			
Enough food supplies	Between Groups	54.378	3	18.126	14.633	.000
	Within Groups	1195.349	965	1.239		
	Total	1249.728	968			
Enough of req. prot. equipment	Between Groups	8.663	3	2.888	1.873	.132
	Within Groups	1487.566	965	1.542		
	Total	1496.229	968			
Personal response plans	Between Groups	19.110	3	6.370	5.666	.001
	Within Groups	1084.909	965	1.124		
	Total	1104.019	968			
Enough household knowledge	Between Groups	16.301	3	5.434	5.961	.001
	Within Groups	879.668	965	.912		
	Total	895.969	968			

First responders preparedness	Between Groups	28.351	3	9.450	9.222	.000
	Within Groups	988.955	965	1.025		
	Total	1017.307	968			
Television	Between Groups	36.330	3	12.110	8.931	.000
	Within Groups	1308.543	965	1.356		
	Total	1344.873	968			
Radio	Between Groups	21.766	3	7.255	6.461	.000
	Within Groups	1083.664	965	1.123		
	Total	1105.430	968			
Newspaper	Between Groups	15.906	3	5.302	3.137	.025
	Within Groups	1631.011	965	1.690		
	Total	1646.916	968			
Internet	Between Groups	21.923	3	7.308	7.688	.000
	Within Groups	917.216	965	.950		
	Total	939.139	968			
Scientific journal	Between Groups	31.655	3	10.552	5.117	.002
	Within Groups	1989.713	965	2.062		
	Total	2021.368	968			
Local medical website	Between Groups	29.056	3	9.685	5.257	.001
	Within Groups	1777.742	965	1.842		
	Total	1806.799	968			
Addressing of a statesman	Between Groups	74.731	3	24.910	17.209	.000
	Within Groups	1396.885	965	1.448		

	Total	1471.616	968			
Addressing of an expert	Between Groups	7.451	3	2.484	3.498	.015
	Within Groups	685.112	965	.710		
	Total	692.563	968			
The social network	Between Groups	9.349	3	3.116	1.571	.195
	Within Groups	1914.292	965	1.984		
	Total	1923.641	968			
Family members	Between Groups	10.426	3	3.475	2.266	.079
	Within Groups	1480.138	965	1.534		
	Total	1490.563	968			
Friends	Between Groups	49.298	3	16.433	11.237	.000
	Within Groups	1411.191	965	1.462		
	Total	1460.489	968			
Local community	Between Groups	.987	3	.329	.218	.884
	Within Groups	1457.793	965	1.511		
	Total	1458.780	968			
Chosen physician	Between Groups	12.955	3	4.318	2.614	.050
	Within Groups	1594.116	965	1.652		
	Total	1607.071	968			
First responders	Between Groups	80.996	3	26.999	14.755	.000
	Within Groups	1765.723	965	1.830		
	Total	1846.718	968			
Non-government org.	Between Groups	11.972	3	3.991	3.430	.017

	Within Groups	1122.808	965	1.164		
	Total	1134.780	968			
Educational institutions	Between Groups	104.629	3	34.876	19.007	.000
	Within Groups	1770.678	965	1.835		
	Total	1875.307	968			
The likelihood of infect.	Between Groups	23.547	3	7.849	6.325	.000
	Within Groups	1197.444	965	1.241		
	Total	1220.991	968			
Respiratory problems	Between Groups	18.495	3	6.165	5.234	.001
	Within Groups	1136.625	965	1.178		
	Total	1155.121	968			
Most severe symptoms	Between Groups	3.958	3	1.319	2.449	.062
	Within Groups	519.900	965	.539		
	Total	523.858	968			
Serious health	Between Groups	5.914	3	1.971	1.862	.134
	Within Groups	1021.702	965	1.059		
	Total	1027.616	968			
Kindergarten or school	Between Groups	6.725	3	2.242	4.134	.006
	Within Groups	523.207	965	.542		
	Total	529.932	968			
A place of greater risk	Between Groups	225.051	3	75.017	37.273	.000
	Within Groups	1942.212	965	2.013		
	Total	2167.263	968			

Losing my job	Between Groups	18.934	3	6.311	2.868	.036
	Within Groups	2123.735	965	2.201		
	Total	2142.669	968			
Prevent behavior	Between Groups	16.433	3	5.478	9.411	.000
	Within Groups	561.703	965	.582		
	Total	578.136	968			
The responsibility	Between Groups	12.472	3	4.157	7.338	.000
	Within Groups	546.754	965	.567		
	Total	559.226	968			
Respecting measures	Between Groups	7.551	3	2.517	1.436	.231
	Within Groups	1691.118	965	1.752		
	Total	1698.669	968			
Information critically	Between Groups	4.942	3	1.647	1.530	.205
	Within Groups	1038.668	965	1.076		
	Total	1043.610	968			
I'm afraid of health	Between Groups	4.811	3	1.604	1.519	.208
	Within Groups	1018.812	965	1.056		
	Total	1023.622	968			
Econ. consequences	Between Groups	7.559	3	2.520	2.902	.034
	Within Groups	837.828	965	.868		
	Total	845.387	968			
Fear of restrictions	Between Groups	11.821	3	3.940	2.283	.078
	Within Groups	1665.374	965	1.726		

	Total	1677.195	968			
I wash my hands with soap	Between Groups	.898	3	.299	1.001	.392
	Within Groups	288.607	965	.299		
	Total	289.505	968			
I'm disinfecting my hands	Between Groups	.969	3	.323	.416	.742
	Within Groups	750.442	965	.778		
	Total	751.412	968			
I wear a protective mask	Between Groups	8.680	3	2.893	1.006	.389
	Within Groups	2775.673	965	2.876		
	Total	2784.353	968			
I wear protective gloves	Between Groups	18.841	3	6.280	2.253	.081
	Within Groups	2689.760	965	2.787		
	Total	2708.601	968			
I don't touch my face	Between Groups	5.341	3	1.780	1.295	.275
	Within Groups	1326.974	965	1.375		
	Total	1332.316	968			
I don't shake hands with acquaintances	Between Groups	15.339	3	5.113	7.131	.000
	Within Groups	691.955	965	.717		
	Total	707.294	968			
I'm not hugging others	Between Groups	18.067	3	6.022	4.409	.004
	Within Groups	1318.019	965	1.366		
	Total	1336.087	968			
I do not kiss others	Between Groups	35.890	3	11.963	8.066	.000

	Within Groups	1431.230	965	1.483		
	Total	1467.121	968			
Maintaining recommended distance	Between Groups	67.183	3	22.394	18.577	.000
	Within Groups	1163.300	965	1.205		
	Total	1230.483	968			
I respect movement restrictions	Between Groups	2.585	3	.862	4.319	.005
	Within Groups	192.554	965	.200		
	Total	195.139	968			
I avoid contacts with the elderly	Between Groups	31.571	3	10.524	15.465	.000
	Within Groups	656.683	965	.681		
	Total	688.254	968			
I don't meet with family members	Between Groups	10.047	3	3.349	2.279	.078
	Within Groups	1417.805	965	1.469		
	Total	1427.851	968			
I use disinfectants for clothes and shoes	Between Groups	29.383	3	9.794	4.972	.002
	Within Groups	1901.019	965	1.970		
	Total	1930.402	968			
Disinfection of pets paws	Between Groups	50.103	3	16.701	5.290	.001
	Within Groups	3046.566	965	3.157		
	Total	3096.669	968			
I have no contacts with pets	Between Groups	162.292	3	54.097	17.638	.000
	Within Groups	2959.677	965	3.067		
	Total	3121.969	968			

Plan isolation household members	Between Groups	39.920	3	13.307	5.735	.001
	Within Groups	2238.965	965	2.320		
	Total	2278.885	968			
Household nutrition plan	Between Groups	95.944	3	31.981	15.646	.000
	Within Groups	1972.458	965	2.044		
	Total	2068.402	968			
I have groceries	Between Groups	16.734	3	5.578	3.888	.009
	Within Groups	1384.573	965	1.435		
	Total	1401.307	968			
I have stock for a month	Between Groups	25.744	3	8.581	3.901	.009
	Within Groups	2122.739	965	2.200		
	Total	2148.483	968			

Explanations – 1 (18-28), 2 (29-38), 3 (39-48), 4 (49-58), 5 (over 59)

**Multiple Comparisons**							
Tukey HSD							
Dependent Variable	(I) Age	(J) Age	Mean Difference (I-J)	Std. Error	Sig.	95% Confidence Interval	
						Lower Bound	Upper Bound
Individual preparedness	1	2	-.279*	.086	.011	-.51	-.04
		3	-.350*	.099	.004	-.62	-.08
		4	.016	.137	1.000	-.36	.39
		5	-1.017*	.364	.042	-2.01	-.02
	2	1	.279*	.086	.011	.04	.51
		3	-.071	.123	.978	-.41	.26
		4	.295	.154	.310	-.13	.72
		5	-.738	.371	.272	-1.75	.28
	3	1	.350*	.099	.004	.08	.62
		2	.071	.123	.978	-.26	.41
		4	.367	.162	.158	-.08	.81
		5	-.667	.374	.385	-1.69	.36

	4	1	-.016	.137	1.000	-.39	.36
		2	-.295	.154	.310	-.72	.13
		3	-.367	.162	.158	-.81	.08
		5	-1.033	.386	.058	-2.09	.02
	5	1	1.017*	.364	.042	.02	2.01
		2	.738	.371	.272	-.28	1.75
		3	.667	.374	.385	-.36	1.69
		4	1.033	.386	.058	-.02	2.09
Household preparedness	1	2	-.208	.090	.143	-.46	.04
		3	-.137	.104	.684	-.42	.15
		4	-.004	.144	1.000	-.40	.39
		5	-.970	.383	.084	-2.02	.08
	2	1	.208	.090	.143	-.04	.46
		3	.071	.129	.981	-.28	.42
		4	.205	.162	.714	-.24	.65
		5	-.762	.390	.290	-1.83	.30
	3	1	.137	.104	.684	-.15	.42
		2	-.071	.129	.981	-.42	.28
		4	.133	.170	.936	-.33	.60
		5	-.833	.394	.214	-1.91	.24
	4	1	.004	.144	1.000	-.39	.40
		2	-.205	.162	.714	-.65	.24
		3	-.133	.170	.936	-.60	.33
		5	-.967	.406	.121	-2.08	.14
	5	1	.970	.383	.084	-.08	2.02
		2	.762	.390	.290	-.30	1.83
		3	.833	.394	.214	-.24	1.91
		4	.967	.406	.121	-.14	2.08
Community preparedness	1	2	.271*	.099	.048	.00	.54
		3	-.229	.114	.266	-.54	.08
		4	.171	.157	.812	-.26	.60
		5	-1.229*	.419	.028	-2.37	-.08
	2	1	-.271*	.099	.048	-.54	.00
		3	-.500*	.141	.004	-.89	-.11
		4	-.100	.177	.980	-.59	.39
		5	-1.500*	.427	.004	-2.67	-.33
	3	1	.229	.114	.266	-.08	.54
		2	.500*	.141	.004	.11	.89
		4	.400	.187	.203	-.11	.91
		5	-1.000	.431	.139	-2.18	.18
	4	1	-.171	.157	.812	-.60	.26
		2	.100	.177	.980	-.39	.59
		3	-.400	.187	.203	-.91	.11
		5	-1.400*	.444	.014	-2.61	-.19
	5	1	1.229*	.419	.028	.08	2.37
		2	1.500*	.427	.004	.33	2.67
		3	1.000	.431	.139	-.18	2.18
		4	1.400*	.444	.014	.19	2.61
State preparedness	1	2	.280*	.101	.044	.00	.56
		3	.028	.117	.999	-.29	.35
		4	.094	.160	.977	-.34	.53

		5	-.839	.427	.284	-2.01	.33
	2	1	-.280*	.101	.044	-.56	.00
		3	-.252	.144	.400	-.65	.14
		4	-.186	.181	.843	-.68	.31
		5	-1.119	.435	.076	-2.31	.07
	3	1	-.028	.117	.999	-.35	.29
		2	.252	.144	.400	-.14	.65
		4	.067	.190	.997	-.45	.59
		5	-.867	.439	.280	-2.07	.33
	4	1	-.094	.160	.977	-.53	.34
		2	.186	.181	.843	-.31	.68
		3	-.067	.190	.997	-.59	.45
		5	-.933	.453	.238	-2.17	.30
	5	1	.839	.427	.284	-.33	2.01
		2	1.119	.435	.076	-.07	2.31
		3	.867	.439	.280	-.33	2.07
		4	.933	.453	.238	-.30	2.17
Enough personal knowledge	1	2	-.366*	.092	.001	-.62	-.11
		3	-.466*	.106	.000	-.76	-.18
		4	-.232	.146	.503	-.63	.17
		5	-.699	.390	.377	-1.76	.37
	2	1	.366*	.092	.001	.11	.62
		3	-.100	.131	.941	-.46	.26
		4	.133	.165	.928	-.32	.58
		5	-.333	.397	.918	-1.42	.75
	3	1	.466*	.106	.000	.18	.76
		2	.100	.131	.941	-.26	.46
		4	.233	.174	.663	-.24	.71
		5	-.233	.401	.978	-1.33	.86
	4	1	.232	.146	.503	-.17	.63
		2	-.133	.165	.928	-.58	.32
		3	-.233	.174	.663	-.71	.24
		5	-.467	.413	.791	-1.60	.66
	5	1	.699	.390	.377	-.37	1.76
		2	.333	.397	.918	-.75	1.42
		3	.233	.401	.978	-.86	1.33
		4	.467	.413	.791	-.66	1.60
Enough personal training	1	2	-.394*	.102	.001	-.67	-.11
		3	-.513*	.118	.000	-.84	-.19
		4	-.346	.162	.207	-.79	.10
		5	-1.513*	.433	.004	-2.70	-.33
	2	1	.394*	.102	.001	.11	.67
		3	-.119	.146	.925	-.52	.28
		4	.048	.183	.999	-.45	.55
		5	-1.119	.441	.083	-2.32	.09
	3	1	.513*	.118	.000	.19	.84
		2	.119	.146	.925	-.28	.52
		4	.167	.193	.910	-.36	.69
		5	-1.000	.445	.163	-2.22	.22
	4	1	.346	.162	.207	-.10	.79
		2	-.048	.183	.999	-.55	.45

		3	-.167	.193	.910	-.69	.36
		5	-1.167	.459	.082	-2.42	.09
	5	1	1.513*	.433	.004	.33	2.70
		2	1.119	.441	.083	-.09	2.32
		3	1.000	.445	.163	-.22	2.22
		4	1.167	.459	.082	-.09	2.42
Enough food supplies	1	2	-.008	.107	1.000	-.30	.29
		3	.258	.124	.231	-.08	.60
		4	1.092*	.171	.000	.62	1.56
		5	-1.008	.455	.175	-2.25	.24
	2	1	.008	.107	1.000	-.29	.30
		3	.267	.153	.410	-.15	.69
		4	1.100*	.193	.000	.57	1.63
		5	-1.000	.464	.198	-2.27	.27
	3	1	-.258	.124	.231	-.60	.08
		2	-.267	.153	.410	-.69	.15
		4	.833*	.203	.000	.28	1.39
		5	-1.267	.468	.054	-2.55	.01
	4	1	-1.092*	.171	.000	-1.56	-.62
		2	-1.100*	.193	.000	-1.63	-.57
		3	-.833*	.203	.000	-1.39	-.28
		5	-2.100*	.483	.000	-3.42	-.78
	5	1	1.008	.455	.175	-.24	2.25
		2	1.000	.464	.198	-.27	2.27
		3	1.267	.468	.054	-.01	2.55
		4	2.100*	.483	.000	.78	3.42
Enough of req. prot. equipment	1	2	.028	.120	.999	-.30	.36
		3	.190	.139	.649	-.19	.57
		4	.390	.190	.245	-.13	.91
		5	-1.377	.508	.053	-2.77	.01
	2	1	-.028	.120	.999	-.36	.30
		3	.162	.171	.878	-.31	.63
		4	.362	.215	.446	-.23	.95
		5	-1.405	.518	.053	-2.82	.01
	3	1	-.190	.139	.649	-.57	.19
		2	-.162	.171	.878	-.63	.31
		4	.200	.226	.903	-.42	.82
		5	-1.567*	.522	.023	-2.99	-.14
	4	1	-.390	.190	.245	-.91	.13
		2	-.362	.215	.446	-.95	.23
		3	-.200	.226	.903	-.82	.42
		5	-1.767*	.538	.009	-3.24	-.30
	5	1	1.377	.508	.053	-.01	2.77
		2	1.405	.518	.053	-.01	2.82
		3	1.567*	.522	.023	.14	2.99
		4	1.767*	.538	.009	.30	3.24
Personal response plans	1	2	-.373*	.102	.003	-.65	-.09
		3	-.278	.118	.130	-.60	.05
		4	-.145	.163	.900	-.59	.30
		5	-1.445*	.434	.008	-2.63	-.26
	2	1	.373*	.102	.003	.09	.65

		3	.095	.146	.966	-.30	.49
		4	.229	.184	.726	-.27	.73
		5	-1.071	.442	.110	-2.28	.14
	3	1	.278	.118	.130	-.05	.60
		2	-.095	.146	.966	-.49	.30
		4	.133	.193	.959	-.39	.66
		5	-1.167	.446	.068	-2.39	.05
	4	1	.145	.163	.900	-.30	.59
		2	-.229	.184	.726	-.73	.27
		3	-.133	.193	.959	-.66	.39
		5	-1.300*	.460	.039	-2.56	-.04
	5	1	1.445*	.434	.008	.26	2.63
		2	1.071	.442	.110	-.14	2.28
		3	1.167	.446	.068	-.05	2.39
		4	1.300*	.460	.039	.04	2.56
Enough household knowledge	1	2	-.319*	.092	.005	-.57	-.07
		3	-.281	.107	.066	-.57	.01
		4	.086	.147	.977	-.31	.49
		5	-1.081*	.391	.046	-2.15	-.01
	2	1	.319*	.092	.005	.07	.57
		3	.038	.132	.998	-.32	.40
		4	.405	.166	.104	-.05	.86
		5	-.762	.398	.311	-1.85	.33
	3	1	.281	.107	.066	-.01	.57
		2	-.038	.132	.998	-.40	.32
		4	.367	.174	.218	-.11	.84
		5	-.800	.402	.271	-1.90	.30
	4	1	-.086	.147	.977	-.49	.31
		2	-.405	.166	.104	-.86	.05
		3	-.367	.174	.218	-.84	.11
		5	-1.167*	.414	.040	-2.30	-.03
	5	1	1.081*	.391	.046	.01	2.15
		2	.762	.398	.311	-.33	1.85
		3	.800	.402	.271	-.30	1.90
		4	1.167*	.414	.040	.03	2.30
First responders preparedness	1	2	.394*	.098	.001	.13	.66
		3	.427*	.113	.002	.12	.74
		4	.227	.156	.589	-.20	.65
		5	-.106	.415	.999	-1.24	1.03
	2	1	-.394*	.098	.001	-.66	-.13
		3	.033	.140	.999	-.35	.42
		4	-.167	.176	.878	-.65	.31
		5	-.500	.423	.762	-1.66	.66
	3	1	-.427*	.113	.002	-.74	-.12
		2	-.033	.140	.999	-.42	.35
		4	-.200	.185	.816	-.71	.31
		5	-.533	.427	.723	-1.70	.63
	4	1	-.227	.156	.589	-.65	.20
		2	.167	.176	.878	-.31	.65
		3	.200	.185	.816	-.31	.71
		5	-.333	.440	.943	-1.54	.87

	5	1	.106	.415	.999	-1.03	1.24
		2	.500	.423	.762	-.66	1.66
		3	.533	.427	.723	-.63	1.70
		4	.333	.440	.943	-.87	1.54
*. The mean difference is significant at the 0.05 level.							

Multiple Comparisons							
Tukey HSD							
Dependent Variable	(I) Age	(J) RD_godine_ispitanika_new	Mean Difference (I-J)	Std. Error	Sig.	95% Confidence Interval	
						Lower Bound	Upper Bound
Television	1	2	.392*	.112	.005	.08	.70
		3	.554*	.130	.000	.20	.91
		4	.121	.179	.962	-.37	.61
		5	-.513	.476	.819	-1.81	.79
	2	1	-.392*	.112	.005	-.70	-.08
		3	.162	.160	.851	-.28	.60
		4	-.271	.202	.663	-.82	.28
		5	-.905	.486	.338	-2.23	.42
	3	1	-.554*	.130	.000	-.91	-.20
		2	-.162	.160	.851	-.60	.28
		4	-.433	.212	.247	-1.01	.15
		5	-1.067	.490	.189	-2.41	.27
	4	1	-.121	.179	.962	-.61	.37
		2	.271	.202	.663	-.28	.82
		3	.433	.212	.247	-.15	1.01
		5	-.633	.505	.720	-2.01	.75
	5	1	.513	.476	.819	-.79	1.81
		2	.905	.486	.338	-.42	2.23
		3	1.067	.490	.189	-.27	2.41
		4	.633	.505	.720	-.75	2.01
Radio	1	2	.036	.102	.997	-.24	.32
		3	.417*	.119	.004	.09	.74
		4	-.383	.163	.130	-.83	.06
		5	-2.250*	.435	.000	-3.44	-1.06
	2	1	-.036	.102	.997	-.32	.24

		3	.381	.146	.070	-.02	.78
		4	-.419	.184	.153	-.92	.08
		5	-2.286*	.443	.000	-3.50	-1.08
	3	1	-.417*	.119	.004	-.74	-.09
		2	-.381	.146	.070	-.78	.02
		4	-.800*	.194	.000	-1.33	-.27
		5	-2.667*	.447	.000	-3.89	-1.45
	4	1	.383	.163	.130	-.06	.83
		2	.419	.184	.153	-.08	.92
		3	.800*	.194	.000	.27	1.33
		5	-1.867*	.461	.001	-3.13	-.61
	5	1	2.250*	.435	.000	1.06	3.44
		2	2.286*	.443	.000	1.08	3.50
		3	2.667*	.447	.000	1.45	3.89
		4	1.867*	.461	.001	.61	3.13
Newspaper	1	2	-.197	.126	.516	-.54	.15
		3	.350	.145	.114	-.05	.75
		4	.017	.200	1.000	-.53	.56
		5	-1.983*	.533	.002	-3.44	-.53
	2	1	.197	.126	.516	-.15	.54
		3	.548*	.179	.020	.06	1.04
		4	.214	.226	.877	-.40	.83
		5	-1.786*	.543	.009	-3.27	-.30
	3	1	-.350	.145	.114	-.75	.05
		2	-.548*	.179	.020	-1.04	-.06
		4	-.333	.237	.624	-.98	.31
		5	-2.333*	.548	.000	-3.83	-.84
	4	1	-.017	.200	1.000	-.56	.53
		2	-.214	.226	.877	-.83	.40
		3	.333	.237	.624	-.31	.98
		5	-2.000*	.565	.004	-3.54	-.46
	5	1	1.983*	.533	.002	.53	3.44
		2	1.786*	.543	.009	.30	3.27
		3	2.333*	.548	.000	.84	3.83
		4	2.000*	.565	.004	.46	3.54
Internet	1	2	-.257	.094	.050	-.52	.00
		3	-.238	.109	.187	-.54	.06
		4	.462*	.150	.018	.05	.87
		5	.195	.400	.989	-.90	1.29
	2	1	.257	.094	.050	.00	.52
		3	.019	.135	1.000	-.35	.39
		4	.719*	.169	.000	.26	1.18

		5	.452	.408	.801	-.66	1.57
	3	1	.238	.109	.187	-.06	.54
		2	-.019	.135	1.000	-.39	.35
		4	.700*	.178	.001	.21	1.19
		5	.433	.411	.830	-.69	1.56
	4	1	-.462*	.150	.018	-.87	-.05
		2	-.719*	.169	.000	-1.18	-.26
		3	-.700*	.178	.001	-1.19	-.21
		5	-.267	.424	.970	-1.43	.89
	5	1	-.195	.400	.989	-1.29	.90
		2	-.452	.408	.801	-1.57	.66
		3	-.433	.411	.830	-1.56	.69
		4	.267	.424	.970	-.89	1.43
Scientific journal	1	2	-.211	.139	.549	-.59	.17
		3	-.092	.161	.979	-.53	.35
		4	-.825*	.221	.002	-1.43	-.22
		5	-1.758*	.588	.024	-3.37	-.15
	2	1	.211	.139	.549	-.17	.59
		3	.119	.198	.975	-.42	.66
		4	-.614	.249	.099	-1.30	.07
		5	-1.548	.599	.074	-3.19	.09
	3	1	.092	.161	.979	-.35	.53
		2	-.119	.198	.975	-.66	.42
		4	-.733*	.262	.041	-1.45	-.02
		5	-1.667*	.605	.047	-3.32	-.01
	4	1	.825*	.221	.002	.22	1.43
		2	.614	.249	.099	-.07	1.30
		3	.733*	.262	.041	.02	1.45
		5	-.933	.623	.565	-2.64	.77
	5	1	1.758*	.588	.024	.15	3.37
		2	1.548	.599	.074	-.09	3.19
		3	1.667*	.605	.047	.01	3.32
		4	.933	.623	.565	-.77	2.64
Addressing of a statesman	1	2	.657*	.116	.000	.34	.97
		3	.571*	.134	.000	.20	.94
		4	.638*	.185	.005	.13	1.14
		5	-.462	.492	.882	-1.81	.88
	2	1	-.657*	.116	.000	-.97	-.34
		3	-.086	.166	.986	-.54	.37
		4	-.019	.209	1.000	-.59	.55
		5	-1.119	.502	.169	-2.49	.25
	3	1	-.571*	.134	.000	-.94	-.20

		2	.086	.166	.986	-.37	.54
		4	.067	.219	.998	-.53	.67
		5	-1.033	.506	.247	-2.42	.35
	4	1	-.638*	.185	.005	-1.14	-.13
		2	.019	.209	1.000	-.55	.59
		3	-.067	.219	.998	-.67	.53
		5	-1.100	.522	.217	-2.53	.33
	5	1	.462	.492	.882	-.88	1.81
		2	1.119	.502	.169	-.25	2.49
		3	1.033	.506	.247	-.35	2.42
		4	1.100	.522	.217	-.33	2.53
Addressing of an expert	1	2	.156	.081	.308	-.07	.38
		3	.261*	.094	.045	.00	.52
		4	.127	.129	.862	-.23	.48
		5	-.606	.345	.399	-1.55	.34
	2	1	-.156	.081	.308	-.38	.07
		3	.105	.116	.896	-.21	.42
		4	-.029	.146	1.000	-.43	.37
		5	-.762	.351	.192	-1.72	.20
	3	1	-.261*	.094	.045	-.52	.00
		2	-.105	.116	.896	-.42	.21
		4	-.133	.153	.908	-.55	.29
		5	-.867	.354	.104	-1.84	.10
	4	1	-.127	.129	.862	-.48	.23
		2	.029	.146	1.000	-.37	.43
		3	.133	.153	.908	-.29	.55
		5	-.733	.365	.263	-1.73	.26
	5	1	.606	.345	.399	-.34	1.55
		2	.762	.351	.192	-.20	1.72
		3	.867	.354	.104	-.10	1.84
		4	.733	.365	.263	-.26	1.73
The social network	1	2	-.017	.136	1.000	-.39	.35
		3	-.098	.157	.971	-.53	.33
		4	.435	.216	.260	-.16	1.03
		5	-1.398	.576	.109	-2.97	.18
	2	1	.017	.136	1.000	-.35	.39
		3	-.081	.194	.994	-.61	.45
		4	.452	.244	.343	-.21	1.12
		5	-1.381	.587	.130	-2.99	.22
	3	1	.098	.157	.971	-.33	.53
		2	.081	.194	.994	-.45	.61
		4	.533	.257	.230	-.17	1.23

		5	-1.300	.593	.183	-2.92	.32
	4	1	-.435	.216	.260	-1.03	.16
		2	-.452	.244	.343	-1.12	.21
		3	-.533	.257	.230	-1.23	.17
		5	-1.833*	.611	.023	-3.50	-.16
	5	1	1.398	.576	.109	-.18	2.97
		2	1.381	.587	.130	-.22	2.99
		3	1.300	.593	.183	-.32	2.92
		4	1.833*	.611	.023	.16	3.50
Family members	1	2	-.234	.119	.286	-.56	.09
		3	-.244	.138	.396	-.62	.13
		4	-.210	.190	.803	-.73	.31
		5	-1.377	.507	.052	-2.76	.01
	2	1	.234	.119	.286	-.09	.56
		3	-.010	.171	1.000	-.48	.46
		4	.024	.215	1.000	-.56	.61
		5	-1.143	.516	.176	-2.55	.27
	3	1	.244	.138	.396	-.13	.62
		2	.010	.171	1.000	-.46	.48
		4	.033	.226	1.000	-.58	.65
		5	-1.133	.521	.190	-2.56	.29
	4	1	.210	.190	.803	-.31	.73
		2	-.024	.215	1.000	-.61	.56
		3	-.033	.226	1.000	-.65	.58
		5	-1.167	.537	.191	-2.63	.30
	5	1	1.377	.507	.052	-.01	2.76
		2	1.143	.516	.176	-.27	2.55
		3	1.133	.521	.190	-.29	2.56
		4	1.167	.537	.191	-.30	2.63
Friends	1	2	-.577*	.117	.000	-.90	-.26
		3	-.496*	.135	.002	-.87	-.13
		4	-.163	.186	.905	-.67	.34
		5	-1.030	.496	.231	-2.38	.32
	2	1	.577*	.117	.000	.26	.90
		3	.081	.167	.989	-.37	.54
		4	.414	.210	.280	-.16	.99
		5	-.452	.505	.899	-1.83	.93
	3	1	.496*	.135	.002	.13	.87
		2	-.081	.167	.989	-.54	.37
		4	.333	.221	.556	-.27	.94
		5	-.533	.510	.834	-1.93	.86
	4	1	.163	.186	.905	-.34	.67

		2	-.414	.210	.280	-.99	.16
		3	-.333	.221	.556	-.94	.27
		5	-.867	.525	.466	-2.30	.57
	5	1	1.030	.496	.231	-.32	2.38
		2	.452	.505	.899	-.93	1.83
		3	.533	.510	.834	-.86	1.93
		4	.867	.525	.466	-.57	2.30
Local community	1	2	-.078	.119	.965	-.40	.25
		3	.008	.137	1.000	-.37	.38
		4	.074	.189	.995	-.44	.59
		5	-1.792*	.504	.004	-3.17	-.42
	2	1	.078	.119	.965	-.25	.40
		3	.086	.170	.987	-.38	.55
		4	.152	.213	.953	-.43	.74
		5	-1.714*	.513	.008	-3.12	-.31
	3	1	-.008	.137	1.000	-.38	.37
		2	-.086	.170	.987	-.55	.38
		4	.067	.224	.998	-.55	.68
		5	-1.800*	.518	.005	-3.22	-.38
	4	1	-.074	.189	.995	-.59	.44
		2	-.152	.213	.953	-.74	.43
		3	-.067	.224	.998	-.68	.55
		5	-1.867*	.534	.004	-3.33	-.41
	5	1	1.792*	.504	.004	.42	3.17
		2	1.714*	.513	.008	.31	3.12
		3	1.800*	.518	.005	.38	3.22
		4	1.867*	.534	.004	.41	3.33
Chosen physician	1	2	.147	.124	.759	-.19	.49
		3	.304	.144	.212	-.09	.70
		4	-.262	.197	.672	-.80	.28
		5	-2.496*	.526	.000	-3.93	-1.06
	2	1	-.147	.124	.759	-.49	.19
		3	.157	.177	.901	-.33	.64
		4	-.410	.223	.352	-1.02	.20
		5	-2.643*	.536	.000	-4.11	-1.18
	3	1	-.304	.144	.212	-.70	.09
		2	-.157	.177	.901	-.64	.33
		4	-.567	.234	.111	-1.21	.07
		5	-2.800*	.541	.000	-4.28	-1.32
	4	1	.262	.197	.672	-.28	.80
		2	.410	.223	.352	-.20	1.02
		3	.567	.234	.111	-.07	1.21

		5	-2.233*	.557	.001	-3.76	-.71
	5	1	2.496*	.526	.000	1.06	3.93
		2	2.643*	.536	.000	1.18	4.11
		3	2.800*	.541	.000	1.32	4.28
		4	2.233*	.557	.001	.71	3.76
Members of the ISS	1	2	.732*	.131	.000	.38	1.09
		3	.622*	.151	.000	.21	1.04
		4	-.078	.208	.996	-.64	.49
		5	-1.911*	.553	.005	-3.42	-.40
	2	1	-.732*	.131	.000	-1.09	-.38
		3	-.110	.186	.977	-.62	.40
		4	-.810*	.234	.005	-1.45	-.17
		5	-2.643*	.564	.000	-4.18	-1.10
	3	1	-.622*	.151	.000	-1.04	-.21
		2	.110	.186	.977	-.40	.62
		4	-.700*	.246	.037	-1.37	-.03
		5	-2.533*	.569	.000	-4.09	-.98
	4	1	.078	.208	.996	-.49	.64
		2	.810*	.234	.005	.17	1.45
		3	.700*	.246	.037	.03	1.37
		5	-1.833*	.587	.016	-3.44	-.23
	5	1	1.911*	.553	.005	.40	3.42
		2	2.643*	.564	.000	1.10	4.18
		3	2.533*	.569	.000	.98	4.09
		4	1.833*	.587	.016	.23	3.44
Non-government org.	1	2	.197	.104	.323	-.09	.48
		3	.278	.120	.144	-.05	.61
		4	.311	.166	.329	-.14	.76
		5	-2.589*	.441	.000	-3.80	-1.38
	2	1	-.197	.104	.323	-.48	.09
		3	.081	.149	.983	-.33	.49
		4	.114	.187	.973	-.40	.63
		5	-2.786*	.450	.000	-4.02	-1.56
	3	1	-.278	.120	.144	-.61	.05
		2	-.081	.149	.983	-.49	.33
		4	.033	.197	1.000	-.50	.57
		5	-2.867*	.454	.000	-4.11	-1.63
	4	1	-.311	.166	.329	-.76	.14
		2	-.114	.187	.973	-.63	.40
		3	-.033	.197	1.000	-.57	.50
		5	-2.900*	.468	.000	-4.18	-1.62
	5	1	2.589*	.441	.000	1.38	3.80

		2	2.786*	.450	.000	1.56	4.02
		3	2.867*	.454	.000	1.63	4.11
		4	2.900*	.468	.000	1.62	4.18
Educational institutions	1	2	.898*	.131	.000	.54	1.26
		3	.565*	.151	.002	.15	.98
		4	-.102	.208	.988	-.67	.47
		5	-1.602*	.554	.032	-3.12	-.09
	2	1	-.898*	.131	.000	-1.26	-.54
		3	-.333	.187	.382	-.84	.18
		4	-1.000*	.235	.000	-1.64	-.36
		5	-2.500*	.565	.000	-4.04	-.96
	3	1	-.565*	.151	.002	-.98	-.15
		2	.333	.187	.382	-.18	.84
		4	-.667	.247	.054	-1.34	.01
		5	-2.167*	.570	.001	-3.72	-.61
	4	1	.102	.208	.988	-.47	.67
		2	1.000*	.235	.000	.36	1.64
		3	.667	.247	.054	-.01	1.34
		5	-1.500	.587	.080	-3.11	.11
	5	1	1.602*	.554	.032	.09	3.12
		2	2.500*	.565	.000	.96	4.04
		3	2.167*	.570	.001	.61	3.72
		4	1.500	.587	.080	-.11	3.11
*. The mean difference is significant at the 0.05 level.							

**Multiple Comparisons**							
Tukey HSD							
Dependent Variable	(I) Age	(J) Age	Mean Difference (I-J)	Std. Error	Sig.	95% Confidence Interval	
						Lower Bound	Upper Bound
The likelihood of infect.	1	2	.394*	.108	.002	.10	.69
		3	.351*	.125	.039	.01	.69
		4	.085	.171	.988	-.38	.55
		5	-.915	.457	.265	-2.16	.33
	2	1	-.394*	.108	.002	-.69	-.10
		3	-.043	.154	.999	-.46	.38
		4	-.310	.193	.498	-.84	.22
		5	-1.310*	.465	.040	-2.58	-.04

	3	1	-.351*	.125	.039	-.69	-.01
		2	.043	.154	.999	-.38	.46
		4	-.267	.203	.684	-.82	.29
		5	-1.267	.470	.055	-2.55	.02
	4	1	-.085	.171	.988	-.55	.38
		2	.310	.193	.498	-.22	.84
		3	.267	.203	.684	-.29	.82
		5	-1.000	.484	.236	-2.32	.32
	5	1	.915	.457	.265	-.33	2.16
		2	1.310*	.465	.040	.04	2.58
		3	1.267	.470	.055	-.02	2.55
		4	1.000	.484	.236	-.32	2.32
Respiratory problems	1	2	-.319*	.105	.020	-.61	-.03
		3	-.353*	.121	.031	-.68	-.02
		4	-.119	.167	.953	-.58	.34
		5	-.653	.445	.585	-1.87	.56
	2	1	.319*	.105	.020	.03	.61
		3	-.033	.150	.999	-.44	.38
		4	.200	.188	.826	-.32	.72
		5	-.333	.454	.948	-1.57	.91
	3	1	.353*	.121	.031	.02	.68
		2	.033	.150	.999	-.38	.44
		4	.233	.198	.764	-.31	.77
		5	-.300	.458	.966	-1.55	.95
	4	1	.119	.167	.953	-.34	.58
		2	-.200	.188	.826	-.72	.32
		3	-.233	.198	.764	-.77	.31
		5	-.533	.472	.790	-1.82	.76
	5	1	.653	.445	.585	-.56	1.87
		2	.333	.454	.948	-.91	1.57
		3	.300	.458	.966	-.95	1.55
		4	.533	.472	.790	-.76	1.82
Most severe symptoms	1	2	-.167	.071	.133	-.36	.03
		3	.067	.082	.928	-.16	.29
		4	-.100	.113	.903	-.41	.21
		5	.500	.302	.462	-.32	1.32
	2	1	.167	.071	.133	-.03	.36
		3	.233	.102	.147	-.04	.51
		4	.067	.128	.985	-.28	.42

		5	.667	.308	.193	-.17	1.51
	3	1	-.067	.082	.928	-.29	.16
		2	-.233	.102	.147	-.51	.04
		4	-.167	.134	.728	-.53	.20
		5	.433	.310	.631	-.42	1.28
	4	1	.100	.113	.903	-.21	.41
		2	-.067	.128	.985	-.42	.28
		3	.167	.134	.728	-.20	.53
		5	.600	.320	.332	-.27	1.47
	5	1	-.500	.302	.462	-1.32	.32
		2	-.667	.308	.193	-1.51	.17
		3	-.433	.310	.631	-1.28	.42
		4	-.600	.320	.332	-1.47	.27
Serious health	1	2	.028	.100	.999	-.24	.30
		3	.228	.116	.279	-.09	.54
		4	.228	.159	.604	-.21	.66
		5	.195	.424	.991	-.96	1.35
	2	1	-.028	.100	.999	-.30	.24
		3	.200	.143	.626	-.19	.59
		4	.200	.179	.799	-.29	.69
		5	.167	.432	.995	-1.01	1.35
	3	1	-.228	.116	.279	-.54	.09
		2	-.200	.143	.626	-.59	.19
		4	.000	.189	1.000	-.52	.52
		5	-.033	.436	1.000	-1.22	1.16
	4	1	-.228	.159	.604	-.66	.21
		2	-.200	.179	.799	-.69	.29
		3	.000	.189	1.000	-.52	.52
		5	-.033	.449	1.000	-1.26	1.19
	5	1	-.195	.424	.991	-1.35	.96
		2	-.167	.432	.995	-1.35	1.01
		3	.033	.436	1.000	-1.16	1.22
		4	.033	.449	1.000	-1.19	1.26
Kindergarten or school	1	2	-.150	.071	.220	-.34	.04
		3	.208	.082	.086	-.02	.43
		4	-.026	.113	.999	-.33	.28
		5	.208	.302	.959	-.62	1.03
	2	1	.150	.071	.220	-.04	.34
		3	.357*	.102	.004	.08	.63

		4	.124	.128	.869	-.23	.47
		5	.357	.307	.773	-.48	1.20
	3	1	-.208	.082	.086	-.43	.02
		2	-.357*	.102	.004	-.63	-.08
		4	-.233	.134	.411	-.60	.13
		5	.000	.310	1.000	-.85	.85
	4	1	.026	.113	.999	-.28	.33
		2	-.124	.128	.869	-.47	.23
		3	.233	.134	.411	-.13	.60
		5	.233	.320	.950	-.64	1.11
	5	1	-.208	.302	.959	-1.03	.62
		2	-.357	.307	.773	-1.20	.48
		3	.000	.310	1.000	-.85	.85
		4	-.233	.320	.950	-1.11	.64
A place of greater risk	1	2	-.559*	.137	.000	-.93	-.19
		3	-1.369*	.158	.000	-1.80	-.94
		4	-1.369*	.218	.000	-1.96	-.77
		5	-1.869*	.580	.012	-3.45	-.28
	2	1	.559*	.137	.000	.19	.93
		3	-.810*	.195	.000	-1.34	-.28
		4	-.810*	.246	.009	-1.48	-.14
		5	-1.310	.592	.175	-2.93	.31
	3	1	1.369*	.158	.000	.94	1.80
		2	.810*	.195	.000	.28	1.34
		4	.000	.258	1.000	-.71	.71
		5	-.500	.597	.919	-2.13	1.13
	4	1	1.369*	.218	.000	.77	1.96
		2	.810*	.246	.009	.14	1.48
		3	.000	.258	1.000	-.71	.71
		5	-.500	.615	.927	-2.18	1.18
	5	1	1.869*	.580	.012	.28	3.45
		2	1.310	.592	.175	-.31	2.93
		3	.500	.597	.919	-1.13	2.13
		4	.500	.615	.927	-1.18	2.18
Losing my job	1	2	-.074	.144	.986	-.47	.32
		3	-.188	.166	.789	-.64	.27
		4	.579	.228	.084	-.05	1.20
		5	-.788	.609	.694	-2.45	.87
	2	1	.074	.144	.986	-.32	.47

		3	-.114	.205	.981	-.67	.45
		4	.652	.258	.085	-.05	1.36
		5	-.714	.620	.779	-2.41	.98
	3	1	.188	.166	.789	-.27	.64
		2	.114	.205	.981	-.45	.67
		4	.767*	.271	.038	.03	1.51
		5	-.600	.626	.873	-2.31	1.11
	4	1	-.579	.228	.084	-1.20	.05
		2	-.652	.258	.085	-1.36	.05
		3	-.767*	.271	.038	-1.51	-.03
		5	-1.367	.645	.213	-3.13	.40
	5	1	.788	.609	.694	-.87	2.45
		2	.714	.620	.779	-.98	2.41
		3	.600	.626	.873	-1.11	2.31
		4	1.367	.645	.213	-.40	3.13
Prevent behavior	1	2	-.306*	.074	.000	-.51	-.10
		3	-.301*	.085	.004	-.53	-.07
		4	-.234	.117	.266	-.55	.09
		5	-.068	.312	1.000	-.92	.79
	2	1	.306*	.074	.000	.10	.51
		3	.005	.105	1.000	-.28	.29
		4	.071	.132	.983	-.29	.43
		5	.238	.318	.945	-.63	1.11
	3	1	.301*	.085	.004	.07	.53
		2	-.005	.105	1.000	-.29	.28
		4	.067	.139	.989	-.31	.45
		5	.233	.321	.950	-.64	1.11
	4	1	.234	.117	.266	-.09	.55
		2	-.071	.132	.983	-.43	.29
		3	-.067	.139	.989	-.45	.31
		5	.167	.331	.987	-.74	1.07
	5	1	.068	.312	1.000	-.79	.92
		2	-.238	.318	.945	-1.11	.63
		3	-.233	.321	.950	-1.11	.64
		4	-.167	.331	.987	-1.07	.74
The responsibility	1	2	-.221*	.073	.020	-.42	-.02
		3	-.283*	.084	.007	-.51	-.05
		4	-.283	.116	.103	-.60	.03
		5	.017	.308	1.000	-.83	.86

	2	1	.221*	.073	.020	.02	.42
		3	-.062	.104	.976	-.35	.22
		4	-.062	.131	.990	-.42	.29
		5	.238	.314	.942	-.62	1.10
	3	1	.283*	.084	.007	.05	.51
		2	.062	.104	.976	-.22	.35
		4	.000	.137	1.000	-.38	.38
		5	.300	.317	.879	-.57	1.17
	4	1	.283	.116	.103	-.03	.60
		2	.062	.131	.990	-.29	.42
		3	.000	.137	1.000	-.38	.38
		5	.300	.327	.890	-.59	1.19
	5	1	-.017	.308	1.000	-.86	.83
		2	-.238	.314	.942	-1.10	.62
		3	-.300	.317	.879	-1.17	.57
		4	-.300	.327	.890	-1.19	.59
Respecting measures	1	2	.259	.128	.253	-.09	.61
		3	.031	.148	1.000	-.37	.43
		4	.131	.203	.968	-.42	.69
		5	-2.169*	.542	.001	-3.65	-.69
	2	1	-.259	.128	.253	-.61	.09
		3	-.229	.182	.720	-.73	.27
		4	-.129	.229	.981	-.76	.50
		5	-2.429*	.552	.000	-3.94	-.92
	3	1	-.031	.148	1.000	-.43	.37
		2	.229	.182	.720	-.27	.73
		4	.100	.241	.994	-.56	.76
		5	-2.200*	.557	.001	-3.72	-.68
	4	1	-.131	.203	.968	-.69	.42
		2	.129	.229	.981	-.50	.76
		3	-.100	.241	.994	-.76	.56
		5	-2.300*	.574	.001	-3.87	-.73
	5	1	2.169*	.542	.001	.69	3.65
		2	2.429*	.552	.000	.92	3.94
		3	2.200*	.557	.001	.68	3.72
		4	2.300*	.574	.001	.73	3.87
Information critically	1	2	-.079	.100	.933	-.35	.19
		3	-.236	.116	.248	-.55	.08
		4	.030	.159	1.000	-.40	.47

		5	-.936	.425	.178	-2.10	.22
	2	1	.079	.100	.933	-.19	.35
		3	-.157	.143	.807	-.55	.23
		4	.110	.180	.974	-.38	.60
		5	-.857	.433	.276	-2.04	.33
	3	1	.236	.116	.248	-.08	.55
		2	.157	.143	.807	-.23	.55
		4	.267	.189	.621	-.25	.78
		5	-.700	.437	.496	-1.89	.49
	4	1	-.030	.159	1.000	-.47	.40
		2	-.110	.180	.974	-.60	.38
		3	-.267	.189	.621	-.78	.25
		5	-.967	.450	.201	-2.20	.26
	5	1	.936	.425	.178	-.22	2.10
		2	.857	.433	.276	-.33	2.04
		3	.700	.437	.496	-.49	1.89
		4	.967	.450	.201	-.26	2.20
I'm afraid of health	1	2	-.007	.099	1.000	-.28	.26
		3	.202	.115	.399	-.11	.52
		4	.202	.158	.704	-.23	.63
		5	.136	.421	.998	-1.02	1.29
	2	1	.007	.099	1.000	-.26	.28
		3	.210	.142	.578	-.18	.60
		4	.210	.179	.766	-.28	.70
		5	.143	.429	.997	-1.03	1.32
	3	1	-.202	.115	.399	-.52	.11
		2	-.210	.142	.578	-.60	.18
		4	.000	.188	1.000	-.51	.51
		5	-.067	.433	1.000	-1.25	1.12
	4	1	-.202	.158	.704	-.63	.23
		2	-.210	.179	.766	-.70	.28
		3	.000	.188	1.000	-.51	.51
		5	-.067	.447	1.000	-1.29	1.15
	5	1	-.136	.421	.998	-1.29	1.02
		2	-.143	.429	.997	-1.32	1.03
		3	.067	.433	1.000	-1.12	1.25
		4	.067	.447	1.000	-1.15	1.29
Econ. consequences	1	2	-.116	.091	.706	-.36	.13
		3	-.292*	.105	.044	-.58	-.01

		4	-.025	.144	1.000	-.42	.37
		5	.742	.384	.302	-.31	1.79
	2	1	.116	.091	.706	-.13	.36
		3	-.176	.129	.652	-.53	.18
		4	.090	.163	.981	-.35	.54
		5	.857	.391	.184	-.21	1.93
	3	1	.292*	.105	.044	.01	.58
		2	.176	.129	.652	-.18	.53
		4	.267	.171	.524	-.20	.73
		5	1.033	.395	.068	-.05	2.11
	4	1	.025	.144	1.000	-.37	.42
		2	-.090	.163	.981	-.54	.35
		3	-.267	.171	.524	-.73	.20
		5	.767	.407	.327	-.35	1.88
	5	1	-.742	.384	.302	-1.79	.31
		2	-.857	.391	.184	-1.93	.21
		3	-1.033	.395	.068	-2.11	.05
		4	-.767	.407	.327	-1.88	.35
Fear of restrictions	1	2	.008	.127	1.000	-.34	.35
		3	.327	.147	.171	-.07	.73
		4	-.240	.202	.759	-.79	.31
		5	-1.373	.538	.081	-2.84	.10
	2	1	-.008	.127	1.000	-.35	.34
		3	.319	.181	.397	-.18	.81
		4	-.248	.228	.814	-.87	.38
		5	-1.381	.549	.088	-2.88	.12
	3	1	-.327	.147	.171	-.73	.07
		2	-.319	.181	.397	-.81	.18
		4	-.567	.240	.126	-1.22	.09
		5	-1.700*	.553	.019	-3.21	-.19
	4	1	.240	.202	.759	-.31	.79
		2	.248	.228	.814	-.38	.87
		3	.567	.240	.126	-.09	1.22
		5	-1.133	.570	.273	-2.69	.43
	5	1	1.373	.538	.081	-.10	2.84
		2	1.381	.549	.088	-.12	2.88
		3	1.700*	.553	.019	.19	3.21
		4	1.133	.570	.273	-.43	2.69

*. The mean difference is significant at the 0.05 level.

Multiple Comparisons							
Tukey HSD							
Dependent Variable	(I) Age	(J) Age	Mean Difference (I-J)	Std. Error	Sig.	95% Confidence Interval	
						Lower Bound	Upper Bound
I wash my hands with soap	1	2	.061	.053	.775	-.08	.21
		3	-.025	.061	.994	-.19	.14
		4	-.091	.084	.813	-.32	.14
		5	-.225	.224	.853	-.84	.39
	2	1	-.061	.053	.775	-.21	.08
		3	-.086	.075	.786	-.29	.12
		4	-.152	.095	.492	-.41	.11
		5	-.286	.228	.720	-.91	.34
	3	1	.025	.061	.994	-.14	.19
		2	.086	.075	.786	-.12	.29
		4	-.067	.100	.963	-.34	.21
		5	-.200	.230	.908	-.83	.43
	4	1	.091	.084	.813	-.14	.32
		2	.152	.095	.492	-.11	.41
		3	.067	.100	.963	-.21	.34
		5	-.133	.237	.980	-.78	.51
	5	1	.225	.224	.853	-.39	.84
		2	.286	.228	.720	-.34	.91
		3	.200	.230	.908	-.43	.83
		4	.133	.237	.980	-.51	.78
I'm disinfecting my hands	1	2	-.025	.085	.998	-.26	.21
		3	.008	.098	1.000	-.26	.28
		4	.141	.135	.835	-.23	.51
		5	-.525	.361	.591	-1.51	.46
	2	1	.025	.085	.998	-.21	.26
		3	.033	.121	.999	-.30	.37
		4	.167	.153	.811	-.25	.58
		5	-.500	.368	.653	-1.50	.50
	3	1	-.008	.098	1.000	-.28	.26
		2	-.033	.121	.999	-.37	.30
		4	.133	.161	.921	-.31	.57
		5	-.533	.371	.603	-1.55	.48
	4	1	-.141	.135	.835	-.51	.23
		2	-.167	.153	.811	-.58	.25

		3	-.133	.161	.921	-.57	.31
		5	-.667	.382	.407	-1.71	.38
	5	1	.525	.361	.591	-.46	1.51
		2	.500	.368	.653	-.50	1.50
		3	.533	.371	.603	-.48	1.55
		4	.667	.382	.407	-.38	1.71
I wear a protective mask	1	2	-.262	.164	.498	-.71	.19
		3	-.009	.189	1.000	-.53	.51
		4	-.209	.260	.929	-.92	.50
		5	-1.809	.694	.070	-3.70	.09
	2	1	.262	.164	.498	-.19	.71
		3	.252	.233	.816	-.39	.89
		4	.052	.294	1.000	-.75	.86
		5	-1.548	.707	.184	-3.48	.38
	3	1	.009	.189	1.000	-.51	.53
		2	-.252	.233	.816	-.89	.39
		4	-.200	.309	.967	-1.04	.64
		5	-1.800	.713	.086	-3.75	.15
	4	1	.209	.260	.929	-.50	.92
		2	-.052	.294	1.000	-.86	.75
		3	.200	.309	.967	-.64	1.04
		5	-1.600	.735	.190	-3.61	.41
	5	1	1.809	.694	.070	-.09	3.70
		2	1.548	.707	.184	-.38	3.48
		3	1.800	.713	.086	-.15	3.75
		4	1.600	.735	.190	-.41	3.61
I wear protective gloves	1	2	-.218	.161	.658	-.66	.22
		3	-.366	.186	.286	-.87	.14
		4	-.399	.256	.525	-1.10	.30
		5	-1.932*	.683	.038	-3.80	-.07
	2	1	.218	.161	.658	-.22	.66
		3	-.148	.230	.968	-.78	.48
		4	-.181	.289	.971	-.97	.61
		5	-1.714	.696	.100	-3.62	.19
	3	1	.366	.186	.286	-.14	.87
		2	.148	.230	.968	-.48	.78
		4	-.033	.304	1.000	-.86	.80
		5	-1.567	.702	.169	-3.49	.35
	4	1	.399	.256	.525	-.30	1.10
		2	.181	.289	.971	-.61	.97
		3	.033	.304	1.000	-.80	.86
		5	-1.533	.724	.213	-3.51	.44

	5	1	1.932*	.683	.038	.07	3.80
		2	1.714	.696	.100	-.19	3.62
		3	1.567	.702	.169	-.35	3.49
		4	1.533	.724	.213	-.44	3.51
I don't touch my face	1	2	.075	.113	.964	-.23	.38
		3	-.134	.131	.843	-.49	.22
		4	-.268	.180	.570	-.76	.22
		5	-.568	.480	.761	-1.88	.74
	2	1	-.075	.113	.964	-.38	.23
		3	-.210	.162	.693	-.65	.23
		4	-.343	.203	.442	-.90	.21
		5	-.643	.489	.682	-1.98	.69
	3	1	.134	.131	.843	-.22	.49
		2	.210	.162	.693	-.23	.65
		4	-.133	.214	.971	-.72	.45
		5	-.433	.493	.905	-1.78	.92
	4	1	.268	.180	.570	-.22	.76
		2	.343	.203	.442	-.21	.90
		3	.133	.214	.971	-.45	.72
		5	-.300	.509	.977	-1.69	1.09
	5	1	.568	.480	.761	-.74	1.88
		2	.643	.489	.682	-.69	1.98
		3	.433	.493	.905	-.92	1.78
		4	.300	.509	.977	-1.09	1.69
I don't shake hands with acquaintances	1	2	-.285*	.082	.005	-.51	-.06
		3	-.228	.095	.113	-.49	.03
		4	-.361*	.130	.044	-.72	-.01
		5	-.428	.346	.730	-1.37	.52
	2	1	.285*	.082	.005	.06	.51
		3	.057	.117	.988	-.26	.38
		4	-.076	.147	.985	-.48	.32
		5	-.143	.353	.994	-1.11	.82
	3	1	.228	.095	.113	-.03	.49
		2	-.057	.117	.988	-.38	.26
		4	-.133	.154	.910	-.55	.29
		5	-.200	.356	.980	-1.17	.77
	4	1	.361*	.130	.044	.01	.72
		2	.076	.147	.985	-.32	.48
		3	.133	.154	.910	-.29	.55
		5	-.067	.367	1.000	-1.07	.94
	5	1	.428	.346	.730	-.52	1.37
		2	.143	.353	.994	-.82	1.11

		3	.200	.356	.980	-.77	1.17
		4	.067	.367	1.000	-.94	1.07
I'm not hugging others	1	2	-.301	.113	.059	-.61	.01
		3	-.334	.130	.078	-.69	.02
		4	-.268	.179	.567	-.76	.22
		5	-.801	.478	.449	-2.11	.51
	2	1	.301	.113	.059	-.01	.61
		3	-.033	.161	1.000	-.47	.41
		4	.033	.202	1.000	-.52	.59
		5	-.500	.487	.843	-1.83	.83
	3	1	.334	.130	.078	-.02	.69
		2	.033	.161	1.000	-.41	.47
		4	.067	.213	.998	-.51	.65
		5	-.467	.491	.877	-1.81	.88
	4	1	.268	.179	.567	-.22	.76
		2	-.033	.202	1.000	-.59	.52
		3	-.067	.213	.998	-.65	.51
		5	-.533	.507	.831	-1.92	.85
	5	1	.801	.478	.449	-.51	2.11
		2	.500	.487	.843	-.83	1.83
		3	.467	.491	.877	-.88	1.81
		4	.533	.507	.831	-.85	1.92
I do not kiss others	1	2	-.448*	.117	.001	-.77	-.13
		3	-.472*	.136	.005	-.84	-.10
		4	-.272	.187	.592	-.78	.24
		5	-.805	.498	.487	-2.17	.56
	2	1	.448*	.117	.001	.13	.77
		3	-.024	.168	1.000	-.48	.43
		4	.176	.211	.920	-.40	.75
		5	-.357	.508	.956	-1.74	1.03
	3	1	.472*	.136	.005	.10	.84
		2	.024	.168	1.000	-.43	.48
		4	.200	.222	.896	-.41	.81
		5	-.333	.512	.966	-1.73	1.07
	4	1	.272	.187	.592	-.24	.78
		2	-.176	.211	.920	-.75	.40
		3	-.200	.222	.896	-.81	.41
		5	-.533	.528	.851	-1.98	.91
	5	1	.805	.498	.487	-.56	2.17
		2	.357	.508	.956	-1.03	1.74
		3	.333	.512	.966	-1.07	1.73
		4	.533	.528	.851	-.91	1.98

Maintaining recommended distance	1	2	-.560*	.106	.000	-.85	-.27
		3	-.546*	.123	.000	-.88	-.21
		4	-.746*	.168	.000	-1.21	-.29
		5	-1.013	.449	.160	-2.24	.21
	2	1	.560*	.106	.000	.27	.85
		3	.014	.151	1.000	-.40	.43
		4	-.186	.190	.866	-.71	.33
		5	-.452	.458	.861	-1.70	.80
	3	1	.546*	.123	.000	.21	.88
		2	-.014	.151	1.000	-.43	.40
		4	-.200	.200	.855	-.75	.35
		5	-.467	.462	.851	-1.73	.80
	4	1	.746*	.168	.000	.29	1.21
		2	.186	.190	.866	-.33	.71
		3	.200	.200	.855	-.35	.75
		5	-.267	.476	.981	-1.57	1.03
	5	1	1.013	.449	.160	-.21	2.24
		2	.452	.458	.861	-.80	1.70
		3	.467	.462	.851	-.80	1.73
		4	.267	.476	.981	-1.03	1.57
I respect movement restrictions	1	2	-.129*	.043	.024	-.25	-.01
		3	-.053	.050	.830	-.19	.08
		4	-.153	.068	.171	-.34	.03
		5	-.153	.183	.920	-.65	.35
	2	1	.129*	.043	.024	.01	.25
		3	.076	.061	.728	-.09	.24
		4	-.024	.077	.998	-.24	.19
		5	-.024	.186	1.000	-.53	.48
	3	1	.053	.050	.830	-.08	.19
		2	-.076	.061	.728	-.24	.09
		4	-.100	.081	.734	-.32	.12
		5	-.100	.188	.984	-.61	.41
	4	1	.153	.068	.171	-.03	.34
		2	.024	.077	.998	-.19	.24
		3	.100	.081	.734	-.12	.32
		5	.000	.194	1.000	-.53	.53
	5	1	.153	.183	.920	-.35	.65
		2	.024	.186	1.000	-.48	.53
		3	.100	.188	.984	-.41	.61
		4	.000	.194	1.000	-.53	.53
I avoid contacts with the elderly	1	2	-.198	.080	.095	-.42	.02
		3	-.031	.092	.997	-.28	.22

		4	.769*	.126	.000	.42	1.11
		5	-.364	.337	.817	-1.29	.56
	2	1	.198	.080	.095	-.02	.42
		3	.167	.114	.584	-.14	.48
		4	.967*	.143	.000	.58	1.36
		5	-.167	.344	.989	-1.11	.77
	3	1	.031	.092	.997	-.22	.28
		2	-.167	.114	.584	-.48	.14
		4	.800*	.150	.000	.39	1.21
		5	-.333	.347	.873	-1.28	.61
	4	1	-.769*	.126	.000	-1.11	-.42
		2	-.967*	.143	.000	-1.36	-.58
		3	-.800*	.150	.000	-1.21	-.39
		5	-1.133*	.358	.014	-2.11	-.16
	5	1	.364	.337	.817	-.56	1.29
		2	.167	.344	.989	-.77	1.11
		3	.333	.347	.873	-.61	1.28
		4	1.133*	.358	.014	.16	2.11
I don't meet with family members	1	2	-.298	.117	.081	-.62	.02
		3	-.089	.135	.966	-.46	.28
		4	.045	.186	.999	-.46	.55
		5	-.822	.496	.460	-2.18	.53
	2	1	.298	.117	.081	-.02	.62
		3	.210	.167	.718	-.25	.67
		4	.343	.210	.477	-.23	.92
		5	-.524	.505	.838	-1.90	.86
	3	1	.089	.135	.966	-.28	.46
		2	-.210	.167	.718	-.67	.25
		4	.133	.221	.974	-.47	.74
		5	-.733	.510	.603	-2.13	.66
	4	1	-.045	.186	.999	-.55	.46
		2	-.343	.210	.477	-.92	.23
		3	-.133	.221	.974	-.74	.47
		5	-.867	.525	.466	-2.30	.57
	5	1	.822	.496	.460	-.53	2.18
		2	.524	.505	.838	-.86	1.90
		3	.733	.510	.603	-.66	2.13
		4	.867	.525	.466	-.57	2.30
I use disinfectants for clothes and shoes	1	2	-.382*	.135	.039	-.75	-.01
		3	-.453*	.157	.032	-.88	-.03
		4	-.253	.215	.764	-.84	.33
		5	-1.453	.574	.084	-3.02	.12

	2	1	.382*	.135	.039	.01	.75
		3	-.071	.193	.996	-.60	.46
		4	.129	.243	.984	-.54	.79
		5	-1.071	.585	.356	-2.67	.53
	3	1	.453*	.157	.032	.03	.88
		2	.071	.193	.996	-.46	.60
		4	.200	.256	.936	-.50	.90
		5	-1.000	.590	.438	-2.61	.61
	4	1	.253	.215	.764	-.33	.84
		2	-.129	.243	.984	-.79	.54
		3	-.200	.256	.936	-.90	.50
		5	-1.200	.608	.280	-2.86	.46
	5	1	1.453	.574	.084	-.12	3.02
		2	1.071	.585	.356	-.53	2.67
		3	1.000	.590	.438	-.61	2.61
		4	1.200	.608	.280	-.46	2.86
Disinfection of pets paws	1	2	.270	.171	.515	-.20	.74
		3	-.488	.198	.101	-1.03	.05
		4	-.688	.272	.086	-1.43	.06
		5	-1.754	.727	.112	-3.74	.23
	2	1	-.270	.171	.515	-.74	.20
		3	-.757*	.245	.017	-1.43	-.09
		4	-.957*	.308	.016	-1.80	-.12
		5	-2.024	.741	.050	-4.05	.00
	3	1	.488	.198	.101	-.05	1.03
		2	.757*	.245	.017	.09	1.43
		4	-.200	.324	.972	-1.08	.68
		5	-1.267	.747	.437	-3.31	.78
	4	1	.688	.272	.086	-.06	1.43
		2	.957*	.308	.016	.12	1.80
		3	.200	.324	.972	-.68	1.08
		5	-1.067	.770	.638	-3.17	1.04
	5	1	1.754	.727	.112	-.23	3.74
		2	2.024	.741	.050	.00	4.05
		3	1.267	.747	.437	-.78	3.31
		4	1.067	.770	.638	-1.04	3.17
I have no contacts with pets	1	2	.437	.169	.073	-.02	.90
		3	-1.034*	.195	.000	-1.57	-.50
		4	.966*	.269	.003	.23	1.70
		5	-2.301*	.716	.012	-4.26	-.34
	2	1	-.437	.169	.073	-.90	.02
		3	-1.471*	.241	.000	-2.13	-.81

		4	.529	.303	.408	-.30	1.36
		5	-2.738*	.730	.002	-4.73	-.74
	3	1	1.034*	.195	.000	.50	1.57
		2	1.471*	.241	.000	.81	2.13
		4	2.000*	.319	.000	1.13	2.87
		5	-1.267	.737	.422	-3.28	.75
	4	1	-.966*	.269	.003	-1.70	-.23
		2	-.529	.303	.408	-1.36	.30
		3	-2.000*	.319	.000	-2.87	-1.13
		5	-3.267*	.759	.000	-5.34	-1.19
	5	1	2.301*	.716	.012	.34	4.26
		2	2.738*	.730	.002	.74	4.73
		3	1.267	.737	.422	-.75	3.28
		4	3.267*	.759	.000	1.19	5.34
Plan isolation household members	1	2	-.427*	.147	.031	-.83	-.03
		3	-.484*	.170	.036	-.95	-.02
		4	-.484	.234	.233	-1.12	.15
		5	-2.284*	.623	.002	-3.99	-.58
	2	1	.427*	.147	.031	.03	.83
		3	-.057	.210	.999	-.63	.52
		4	-.057	.264	1.000	-.78	.66
		5	-1.857*	.635	.029	-3.59	-.12
	3	1	.484*	.170	.036	.02	.95
		2	.057	.210	.999	-.52	.63
		4	.000	.277	1.000	-.76	.76
		5	-1.800*	.641	.040	-3.55	-.05
	4	1	.484	.234	.233	-.15	1.12
		2	.057	.264	1.000	-.66	.78
		3	.000	.277	1.000	-.76	.76
		5	-1.800	.660	.051	-3.60	.00
	5	1	2.284*	.623	.002	.58	3.99
		2	1.857*	.635	.029	.12	3.59
		3	1.800*	.641	.040	.05	3.55
		4	1.800	.660	.051	.00	3.60
Household nutrition plan	1	2	-.720*	.138	.000	-1.10	-.34
		3	-.772*	.160	.000	-1.21	-.34
		4	-.506	.219	.144	-1.10	.09
		5	-1.839*	.585	.015	-3.44	-.24
	2	1	.720*	.138	.000	.34	1.10
		3	-.052	.197	.999	-.59	.49
		4	.214	.248	.910	-.46	.89
		5	-1.119	.596	.330	-2.75	.51

	3	1	.772*	.160	.000	.34	1.21
		2	.052	.197	.999	-.49	.59
		4	.267	.260	.844	-.44	.98
		5	-1.067	.601	.389	-2.71	.58
	4	1	.506	.219	.144	-.09	1.10
		2	-.214	.248	.910	-.89	.46
		3	-.267	.260	.844	-.98	.44
		5	-1.333	.620	.199	-3.03	.36
	5	1	1.839*	.585	.015	.24	3.44
		2	1.119	.596	.330	-.51	2.75
		3	1.067	.601	.389	-.58	2.71
		4	1.333	.620	.199	-.36	3.03
I have groceries	1	2	-.357*	.116	.018	-.67	-.04
		3	-.176	.134	.681	-.54	.19
		4	-.276	.184	.561	-.78	.23
		5	-.809	.490	.464	-2.15	.53
	2	1	.357*	.116	.018	.04	.67
		3	.181	.165	.808	-.27	.63
		4	.081	.207	.995	-.49	.65
		5	-.452	.499	.895	-1.82	.91
	3	1	.176	.134	.681	-.19	.54
		2	-.181	.165	.808	-.63	.27
		4	-.100	.218	.991	-.70	.50
		5	-.633	.504	.718	-2.01	.74
	4	1	.276	.184	.561	-.23	.78
		2	-.081	.207	.995	-.65	.49
		3	.100	.218	.991	-.50	.70
		5	-.533	.519	.843	-1.95	.89
	5	1	.809	.490	.464	-.53	2.15
		2	.452	.499	.895	-.91	1.82
		3	.633	.504	.718	-.74	2.01
		4	.533	.519	.843	-.89	1.95
I have stock for a month	1	2	-.407*	.143	.036	-.80	-.02
		3	-.312	.166	.326	-.76	.14
		4	-.345	.227	.550	-.97	.28
		5	-1.479	.606	.106	-3.14	.18
	2	1	.407*	.143	.036	.02	.80
		3	.095	.204	.990	-.46	.65
		4	.062	.257	.999	-.64	.76
		5	-1.071	.618	.414	-2.76	.62
	3	1	.312	.166	.326	-.14	.76
		2	-.095	.204	.990	-.65	.46

		4	-.033	.270	1.000	-.77	.70
		5	-1.167	.624	.334	-2.87	.54
	4	1	.345	.227	.550	-.28	.97
		2	-.062	.257	.999	-.76	.64
		3	.033	.270	1.000	-.70	.77
		5	-1.133	.643	.396	-2.89	.62
	5	1	1.479	.606	.106	-.18	3.14
		2	1.071	.618	.414	-.62	2.76
		3	1.167	.624	.334	-.54	2.87
		4	1.133	.643	.396	-.62	2.89
*. The mean difference is significant at the 0.05 level.							

Gender – Anova tests

Post hoc tests are not performed for because there are fewer than three groups related to gender.

ANOVA						
		Sum of Squares	df	Mean Square	F	Sig.
Individual preparedness	Between Groups	.144	1	.144	.179	.673
	Within Groups	786.446	973	.808		
	Total	786.591	974			
Household preparedness	Between Groups	1.152	1	1.152	1.311	.252
	Within Groups	855.057	973	.879		
	Total	856.209	974			
Community preparedness	Between Groups	17.683	1	17.683	16.873	.000
	Within Groups	1019.708	973	1.048		
	Total	1037.391	974			
State preparedness	Between Groups	13.779	1	13.779	12.751	.000
	Within Groups	1051.451	973	1.081		
	Total	1065.231	974			
Enough personal knowledge	Between Groups	1.221	1	1.221	1.311	.252
	Within Groups	905.942	973	.931		
	Total	907.163	974			
Enough personal training	Between Groups	4.570	1	4.570	3.958	.047
	Within Groups	1123.467	973	1.155		
	Total	1128.037	974			
Enough food supplies	Between Groups	9.074	1	9.074	7.068	.008
	Within Groups	1249.136	973	1.284		

	Total	1258.209	974			
Enough of req. prot. equipment	Between Groups	4.188	1	4.188	2.706	.100
	Within Groups	1505.505	973	1.547		
	Total	1509.692	974			
Personal response plans	Between Groups	.821	1	.821	.716	.398
	Within Groups	1115.789	973	1.147		
	Total	1116.609	974			
Enough household knowledge	Between Groups	.901	1	.901	.971	.325
	Within Groups	902.736	973	.928		
	Total	903.637	974			
First responders preparedness	Between Groups	3.577	1	3.577	3.412	.065
	Within Groups	1019.986	973	1.048		
	Total	1023.563	974			
Television	Between Groups	53.134	1	53.134	39.906	.000
	Within Groups	1295.536	973	1.331		
	Total	1348.671	974			
Radio	Between Groups	.065	1	.065	.055	.814
	Within Groups	1142.243	973	1.174		
	Total	1142.308	974			
Newspaper	Between Groups	6.977	1	6.977	4.066	.044
	Within Groups	1669.571	973	1.716		
	Total	1676.548	974			
Internet	Between Groups	.088	1	.088	.090	.764
	Within Groups	945.365	973	.972		
	Total	945.452	974			
Scientific journal	Between Groups	.076	1	.076	.036	.849
	Within Groups	2044.207	973	2.101		
	Total	2044.283	974			
Local medical website	Between Groups	42.670	1	42.670	23.269	.000
	Within Groups	1784.247	973	1.834		
	Total	1826.917	974			
Addressing of a statesman	Between Groups	141.533	1	141.533	103.236	.000
	Within Groups	1333.950	973	1.371		
	Total	1475.483	974			
Addressing of an expert	Between Groups	35.976	1	35.976	53.105	.000
	Within Groups	659.157	973	.677		

	Total	695.132	974			
The social network	Between Groups	4.865	1	4.865	2.450	.118
	Within Groups	1932.083	973	1.986		
	Total	1936.948	974			
Family members	Between Groups	.385	1	.385	.249	.618
	Within Groups	1501.978	973	1.544		
	Total	1502.363	974			
Friends	Between Groups	1.824	1	1.824	1.208	.272
	Within Groups	1469.505	973	1.510		
	Total	1471.329	974			
Local community	Between Groups	.854	1	.854	.560	.454
	Within Groups	1482.955	973	1.524		
	Total	1483.809	974			
Chosen physician	Between Groups	1.377	1	1.377	.814	.367
	Within Groups	1645.393	973	1.691		
	Total	1646.769	974			
Members of the ISS	Between Groups	.355	1	.355	.184	.668
	Within Groups	1873.177	973	1.925		
	Total	1873.532	974			
Non-government org.	Between Groups	1.033	1	1.033	.853	.356
	Within Groups	1177.275	973	1.210		
	Total	1178.308	974			
The likelihood of infect.	Between Groups	.030	1	.030	.024	.877
	Within Groups	1232.961	973	1.267		
	Total	1232.991	974			
Respiratory problems	Between Groups	15.351	1	15.351	13.014	.000
	Within Groups	1147.725	973	1.180		
	Total	1163.077	974			
Most severe symptoms	Between Groups	1.682	1	1.682	3.089	.079
	Within Groups	529.789	973	.544		
	Total	531.471	974			
Serious health	Between Groups	5.108	1	5.108	4.797	.029
	Within Groups	1036.160	973	1.065		
	Total	1041.268	974			
Kindergarten or school	Between Groups	.002	1	.002	.004	.947
	Within Groups	531.690	973	.546		

	Total	531.692	974			
A place of greater risk	Between Groups	1.163	1	1.163	.518	.472
	Within Groups	2182.966	973	2.244		
	Total	2184.129	974			
Losing my job	Between Groups	4.663	1	4.663	2.105	.147
	Within Groups	2155.207	973	2.215		
	Total	2159.871	974			
Prevent behavior	Between Groups	.072	1	.072	.121	.728
	Within Groups	579.565	973	.596		
	Total	579.637	974			
The responsibility	Between Groups	.606	1	.606	1.053	.305
	Within Groups	560.163	973	.576		
	Total	560.769	974			
Respecting measures	Between Groups	.859	1	.859	.484	.487
	Within Groups	1728.489	973	1.776		
	Total	1729.348	974			
Information critically	Between Groups	.997	1	.997	.925	.336
	Within Groups	1049.003	973	1.078		
	Total	1050.000	974			
I'm afraid of health	Between Groups	20.991	1	20.991	20.248	.000
	Within Groups	1008.701	973	1.037		
	Total	1029.692	974			
Econ. consequences	Between Groups	21.190	1	21.190	24.505	.000
	Within Groups	841.370	973	.865		
	Total	862.560	974			
Fear of restrictions	Between Groups	.256	1	.256	.147	.701
	Within Groups	1694.513	973	1.742		
	Total	1694.769	974			
I wash my hands with soap	Between Groups	11.483	1	11.483	40.144	.000
	Within Groups	278.326	973	.286		
	Total	289.809	974			
I'm disinfecting my hands	Between Groups	29.707	1	29.707	39.958	.000
	Within Groups	723.376	973	.743		
	Total	753.083	974			
I wear a protective mask	Between Groups	39.554	1	39.554	13.927	.000
	Within Groups	2763.369	973	2.840		

	Total	2802.923	974			
I wear protective gloves	Between Groups	50.025	1	50.025	18.169	.000
	Within Groups	2679.015	973	2.753		
	Total	2729.040	974			
I don't touch my face	Between Groups	35.525	1	35.525	26.587	.000
	Within Groups	1300.112	973	1.336		
	Total	1335.637	974			
I don't shake hands with acquaintances	Between Groups	10.597	1	10.597	14.784	.000
	Within Groups	697.440	973	.717		
	Total	708.037	974			
I'm not hugging others	Between Groups	2.710	1	2.710	1.973	.160
	Within Groups	1336.453	973	1.374		
	Total	1339.163	974			
I do not kiss others	Between Groups	4.311	1	4.311	2.862	.091
	Within Groups	1465.652	973	1.506		
	Total	1469.963	974			
Maintaining recommended distance	Between Groups	.011	1	.011	.009	.926
	Within Groups	1234.826	973	1.269		
	Total	1234.837	974			
I respect movement restrictions	Between Groups	.022	1	.022	.107	.743
	Within Groups	195.209	973	.201		
	Total	195.231	974			
I avoid contacts with the elderly	Between Groups	8.625	1	8.625	12.333	.000
	Within Groups	680.452	973	.699		
	Total	689.077	974			
I don't meet with family members	Between Groups	53.768	1	53.768	37.974	.000
	Within Groups	1377.685	973	1.416		
	Total	1431.452	974			
I use disinfectants for clothes and shoes	Between Groups	20.698	1	20.698	10.486	.001
	Within Groups	1920.570	973	1.974		
	Total	1941.268	974			
Disinfection of pets paws	Between Groups	89.518	1	89.518	28.797	.000
	Within Groups	3024.630	973	3.109		
	Total	3114.148	974			
I have no contacts with pets	Between Groups	7.688	1	7.688	2.378	.123
	Within Groups	3146.004	973	3.233		

	Total	3153.692	974			
Plan isolation household members	Between Groups	.031	1	.031	.013	.909
	Within Groups	2306.701	973	2.371		
	Total	2306.732	974			
Household nutrition plan	Between Groups	12.156	1	12.156	5.707	.017
	Within Groups	2072.484	973	2.130		
	Total	2084.640	974			
I have groceries	Between Groups	2.838	1	2.838	1.970	.161
	Within Groups	1401.679	973	1.441		
	Total	1404.517	974			
I have stock for a month	Between Groups	.026	1	.026	.012	.915
	Within Groups	2159.827	973	2.220		
	Total	2159.852	974			
